# The global landscape of sequence diversity

**DOI:** 10.1186/gb-2007-8-11-r238

**Published:** 2007-11-08

**Authors:** José Manuel Peregrín-Álvarez, John Parkinson

**Affiliations:** 1 Molecular Structure and Function, Hospital for Sick Children, 555 University Avenue, Toronto, ON M5G 1X8, Canada; 2 Departments of Biochemistry and Molecular Genetics, 1 King's College Circle, University of Toronto, Toronto, ON M5S 1A1, Canada

## Abstract

**Background:**

Systematic comparisons between genomic sequence datasets have revealed a wide spectrum of sequence specificity from sequences that are highly conserved to those that are specific to individual species. Due to the limited number of fully sequenced eukaryotic genomes, analyses of this spectrum have largely focused on prokaryotes. Combining existing genomic datasets with the partial genomes of 193 eukaryotes derived from collections of expressed sequence tags, we performed a quantitative analysis of the sequence specificity spectrum to provide a global view of the origins and extent of sequence diversity across the three domains of life.

**Results:**

Comparisons with prokaryotic datasets reveal a greater genetic diversity within eukaryotes that may be related to differences in modes of genetic inheritance. Mapping this diversity within a phylogenetic framework revealed that the majority of sequences are either highly conserved or specific to the species or taxon from which they derive. Between these two extremes, several evolutionary landmarks consisting of large numbers of sequences conserved within specific taxonomic groups were identified. For example, 8% of sequences derived from metazoan species are specific and conserved within the metazoan lineage. Many of these sequences likely mediate metazoan specific functions, such as cell-cell communication and differentiation.

**Conclusion:**

Through the use of partial genome datasets, this study provides a unique perspective of sequence conservation across the three domains of life. The provision of taxon restricted sequences should prove valuable for future computational and biochemical analyses aimed at understanding evolutionary and functional relationships.

## Abstract 2

Comparison of genomic and EST sequences reveals a greater genetic diversity within eukaryotes than prokaryotes and enables identification of taxon-specific sequences.

## Background

Sequence space - the sum of all distinct protein and DNA sequences - is vast. A single copy of every possible 300 residue protein, for example, would fill several universes [1]. In consequence, the evolution of genes, which mainly occurs through duplication, divergence and recombination [2], has led to only a small sampling of the available space. Systematic comparisons of proteins and coding sequences from existing genome scale datasets from a wide variety of organisms [3] are beginning to yield insights into the generation and extent of sequence diversity across life [4-9]. In addition to the continued discovery of apparently novel genes and gene families with each new sampled organism, these studies are beginning to reveal a wide spectrum of sequence specificity. At one extreme, sequences may be highly conserved across many different species from several evolutionarily distant lineages. The identification of these conserved sequences, perhaps constrained through extensive interactions with several different protein partners (for example, histones [10]), can provide clues about the genome content of the last universal common ancestor [11]. At the other end of the spectrum of sequence specificity, sequences may be unique to a single species [12-14]. These so-called ORFan sequences are thought to represent sequences that are either remote homologs of known gene families, difficult to detect through current tools, or sequences that may have arisen *de novo *from non-coding sequences. However, it should be noted that many ORFans may simply arise as a consequence of incomplete sampling of sequence space. Further exploration of this space through additional sequencing is, therefore, expected to reduce their incidence [9].

While the exploration of this spectrum of sequence specificity is being usefully exploited to derive novel evolutionary and functional relationships, much of the focus has centered on sequences of prokaryotic origin. This is primarily due to the greater number of bacterial genomes that have been sequenced to date. However, the high incidence of lateral gene transfer (LGT) events in prokaryotes has resulted in the lack of a robustly defined phylogeny and, hence, studies of sequence diversity have largely focused on the identification and characterization of sequences at the two extremes of the spectrum [14-18]. On the other hand, while the taxonomic relationships in eukaryotes are more clearly defined, detailed systematic analyses of diversity within eukaryotes on the basis of fully sequenced genomes are precluded by the limited number and phylogenetic range of organisms that have been sequenced [19].

Aside from fully sequenced genomes, a large amount of sequence data has been, and continues to be, generated within the context of survey sequencing projects. Metagenomics projects, such as those exploring sequence diversity in the human gut or niches within the ocean, are continuing to expand the known repertoire of protein families [4,9,20]. However, due to the methods employed, these projects tend to focus on prokaryotes. Furthermore, the use of shotgun sequencing applied to heterogeneous samples leads to difficulties in assessing the taxonomic relationships within these datasets. More pertinently, over the past decade a plethora of sequencing projects has been initiated with the express aim of generating sequence data in the form of expressed sequence tags (ESTs) from eukaryotic taxa that have previously been neglected by genome sequencing initiatives (for example, [21-24]). As we have previously demonstrated, it is possible to use these datasets to identify non-redundant sets of genes associated with each species [25,26]. Due to the incomplete nature of these collections of genes, we term such collections 'partial genomes'. These datasets provide a tremendous source of eukaryotic sequence information from a diverse range of species with well defined taxonomic relationships and have recently been exploited to explore genetic diversity within, for example, Nematoda [24] and the Coleoptera [21]. In a previous study we collated and processed 1.2 million ESTs from 193 species of eukaryotes to create 546,451 putative gene sequences [26]. Here we use these data to supplement 741,098 protein sequences from 198 fully sequenced genome datasets to perform a systematic analysis of sequence diversity across the three domains of life. Uniquely, we place our findings in the context of previously defined taxonomic relationships to identify and characterize landmarks of sequence evolution within the tree of life. These evolutionary datasets are provided through a publicly accessible online resource [27].

## Results

### Sampling sequence space within the three domains of life

Previous studies of bacterial genomes have shown that as new genome sequences become available, there is an almost constant increase in new coding sequences discovered [17,28]. From the analysis of 1.28 million sequences (Table 1), we extend these studies to examine the extent to which sequence space has been sampled across the three domains of life (Additional data files 1-3). In the following, we quantify the accumulation of 'distinct' coding sequences and gene families with the addition of genome datasets across a broad set of different taxonomic groups. In the context of this study we define a sequence as 'distinct' if it does not possess significant sequence similarity, on the basis of exhaustive BLAST searches, to previously sampled sequences.

**Table 1:** Taxonomic distribution of genomic datasets used in this study

Set	Taxonomic group	No. of species	No. of sequences
Fully sequenced genomes	Archaea	19	42,079
	Bacteria	160	477,069
	Eukarya	19	221,950
	**Total**	**198**	**741,098**
			
Partial genomes	Protists	17	43,550
	Viridiplantae	76	221,896
	Fungi	27	62,528
	Arthropods	17	22,528
	Nematodes	31	95,341
	Lophotrochozoa	4	10,365
	Deuterostomes	21	90,243
	**Total**	**193**	**546,451**

**Figure 1:**
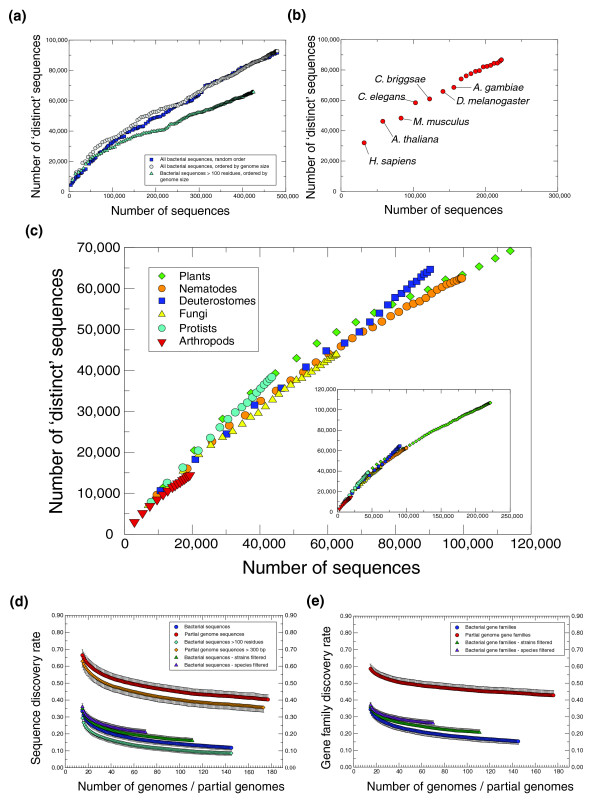
Sequence discovery rates across various taxonomic groups. **(a) **Discovery of 'distinct' sequences as a function of sampled bacterial genomes. Distinct sequences are defined as those that do not share significant sequence similarity with a sequence in a previously sampled genome. Each point represents the addition of a new genome, ordered either by the number of sequences (largest first) or by random. Two datasets are shown: one that considers all sequences; and one that considers only sequences that consist of more than 100 residues. **(b) **Discovery of distinct sequences in fully sequenced eukaryotic genomes. Genome addition was ordered by the number of sequences (largest first). Certain points are labeled to indicate the species added to show how the addition of closely related species influences the local gradient of the graph. **(c) **Rate of distinct sequence discovery within various taxonomic groupings of eukaryotic partial genomes. As before, each point represents the addition of a new partial genome (largest first), and color indicates the taxonomic group sampled. It should be noted that the classification of Protista as a group is historical and has recently been shown to consist of several paraphyletic taxa, many of which (including the species examined here) are considered basal to the root of Eukarya [29]. The inset graph provides an expanded display. **(d) **Rate of sequence discovery as a function of genomes sampled for both bacterial genomes and eukaryotic partial genomes. Each point represents the average and standard deviations of the rate of distinct sequence discovery over a sliding window representing the cumulative addition of 30 complete or partial genomes, obtained from 400 random orderings of genome addition (see Materials and methods for more details). The six data series include sequences from all bacterial and all partial genomes, bacterial sequences > 100 residues in length, partial genome sequences > 300 bp in length and two 'restricted' groups of bacterial sequences: those from a collection of genomes with only a single (largest) representative from each species ('strains filtered'); and those from a collection of genomes with only a single (again largest) representative from each genus ('species filtered'). **(e) **Rate of gene family discovery for partial and bacterial genomes. Gene families include singletons (families with only a single sequence representative) and were obtained with reference to the COGENT database for bacteria, or determined through an equivalent clustering procedure for partial genomes (see Materials and methods). As for (d), each point represents the average and standard deviations of the rate of gene family discovery over a sliding window representing the cumulative addition of 30 complete or partial genomes, obtained from 400 random orderings of genome addition (see Materials and methods for more details). Also shown are the gene family discovery rates for the two 'restricted' groups of bacterial sequences mentioned above.

Consistent with previous studies, we find an almost constant increase in the discovery of distinct sequences as new genomes are sequenced (Figure 1a, b) [6,17]. In bacteria, of 477,069 sequences (from 161 genomes sampled), 92,763 were defined as distinct (Figure 1a). This gives an 'overall sequence discovery rate' (OSDR) of 19.5%, compared with 39% for eukaryotes (86,665/221,948 for 19 genomes) and 37.8% for Archaea (15,903/42,079 for 19 genomes) (Table 2). From the bacterial datasets it is obvious that as more genomes are added, the rate of new sequence discovery decreases. Hence, the disparity in OSDR between the bacterial and the other two datasets may stem from the difference in the number of genomes sampled. For example, random samples of 19 bacterial genomes yields an OSDR of 40.3 ± 3.3% (*n *= 400), comparable to the archaeal and eukaryotic datasets. At this time, however, the limited number of genomes available for Archaea and Eukarya negates our ability to predict with any confidence the future trends associated with these datasets. Furthermore, at least for eukaryotes, the OSDR may be skewed by the close evolutionary relationships of some of the genomes sampled (for example, *Caenorhabditis briggsae *and *C. elegans*; *Mus musculus *and *Homo sapiens*; Figure 1b). For example, sequence similarity analyses of 16 highly conserved gene families found that sequences from the eukaryotic genomes tended to be more closely related than those from randomly selected sets of equivalent numbers of bacterial genomes (Additional data file 4). On the other hand, with sequence data from 193 different species of eukaryotes, partial genomes offer a depth and breadth of sampling that can be usefully exploited to examine sequence diversity in more detail (Figure 1c and Table 2). For the entire dataset we observe an almost constant (but decreasing) rate of new sequence discovery (OSDR = 53.7%). Interestingly, the rate varied between different taxonomic groups (Figure 1c). Plants had the lowest rate (OSDR = 48.3%), reflecting the close evolutionary relationships of species from this group (70/76 datasets were derived from Spermatophyta). Protists had the highest rate (OSDR = 88.1%), highlighting their huge diversity and an associated lack of sequence sampling for these organisms [29].

**Table 2:** Sequence and gene family discovery rates for various complete and partial genome datasets

		Sequence rate (%)^†^		Family rate (%)^†^	
			
Dataset*	No. of complete/partial genomes	OSDR	CSDR	*OGDR*	CGDR
CG Archaea	19	37.8	-	38.7	-
CG Bacteria	161	19.5	11.8 (± 1.5)	22.4	15.4 (± 1.8)
CG Bacteria strains filtered	127	28.4	15.9 (± 1.5)	26.6	20.6 (± 1.7)
CG Bacteria	127		13.4 (± 1.7)		17.0 (± 2.0)
CG Bacteria species filtered	86	23.2	20.9 (± 1.6)	31.5	26.1 (± 1.6)
CG Bacteria	86		16.3 (± 1.8)		19.9 (± 2.1)
CG Eukarya	19	39.0	-	30.8	-
PG All	193	53.7	40.3 (± 2.9)	47.7	42.8 (± 2.8)
PG Arthropods	16	74.7	-	66.4	-
PG Deuterostomes	21	71.7	-	60.8	-
PG Fungi	27	70.2	-	60.2	-
PG Nematodes	31	62.8	-	47.0	-
PG Protists	17	88.1	-	71.5	-
PG Viridiplantae	76	48.3	-	37.8	-
CG Bacteria sequences > 100 residues	161	-	8.6 (± 1.4)	-	-
PG Sequences > 300 bp	193	-	35.6 (± 2.8)	-	-

*CG, complete genome datasets; PG, partial genome datasets; 'strains filtered' indicate that only a single species representative was included in the analysis; 'species filtered' indicate that only a single genus representative was included in the analysis. ^†^OSDR, overall sequence discovery rate (the total number of distinct sequences/total number of sequences); CSDR, current sequence discovery rate (obtained from Figure 1d, e); OGDR, overall gene family discovery rate (total number of families/total number of sequences); CGDR, current gene family discovery rate (obtained from Figure 1d, e).

Since the rate of sequence discovery decreases as a function of accumulated genomes, we were interested in determining the 'current sequence discovery rate' (CSDR), here defined as the percentage of distinct sequences associated with the last genome added to the existing dataset. From Figure 1d we obtain CSDR values of 11.8% for the 161 bacterial genomes (consistent with previous estimates [17]) and 40.3% for the 193 eukaryotic partial genomes (Table 2). Together with the large difference in OSDR, these values suggest that the eukaryotic partial genome datasets are more genetically diverse than the bacterial datasets. Previously, it has been suggested that many apparently novel sequences may rather represent artifacts of short, potentially mis-annotated sequences. Therefore, while subsequent studies have shown that many short sequences do indeed encode functional proteins [14,17], it is possible that short sequences may be responsible for the observed increase in diversity associated with the partial genome datasets. We therefore repeated these analyses using only sequences greater than 100 residues in the bacterial datasets and 300 bp in the partial genome datasets (Figure 1a, d). Although we noted decreases in the rate of sequence discovery, excluding the shorter sequences resulted in similar trends to those observed in the full sequence datasets (CSDR = 8.6% for bacterial genomes and 35.6% for partial genomes; Table 2).

### Impact of sampling bias and genome duplication on genetic diversity

Rather than being randomly sampled, selection of organisms for genome sequencing projects have primarily been motivated by medical or economic concerns. This bias has resulted in the generation of sequences from many closely related strains of bacteria (for example, five strains of *Staphylococcus aureus *are represented in our dataset) that could affect sequence discovery rates (Additional data file 5). Recalculations of sequence discovery rates using only a single representative (largest) for each bacterial species (127 genomes total) or only a single representative (largest) for each bacterial genus (86 genomes total) increased CSDR by 2.5% and 4.6%, respectively (Figure 1e and Table 2). However, despite these increases, rates of sequence discovery are still considerably lower than those obtained for the partial genome datasets in which no genomes were removed.

In addition to sampling biases in bacteria, whole genome duplication events observed for many eukaryotic lineages could result in the retention of many replicates of similar genes and, thus, contribute to the higher sequence discovery rates observed in eukaryotes. We therefore repeated our analyses using gene families (Table 2 and Additional data files 2 and 3). For both bacterial and partial genome datasets, the 'current gene family discovery rate' (CGDR - similar to CSDR but applied to gene families) was slightly higher (15.4% and 42.8%, respectively) than the respective CSDRs (Figure 1e and Table 2). However, the large difference observed between the two datasets indicates that genome-specific duplication events do not have a major influence on sequence discovery rates. Furthermore, analyses of gene family discovery rates within different eukaryotic taxa revealed similar trends to those observed for sequence discovery rates (Additional data file 5).

Together these results suggest that the observed differences in sequence discovery rates between the various taxa are not simply due to sequencing biases or lineage specific duplications, but rather reflect genuine differences in sequence diversity.

### Sequence comparisons between the three domains of life

**Figure 2:**
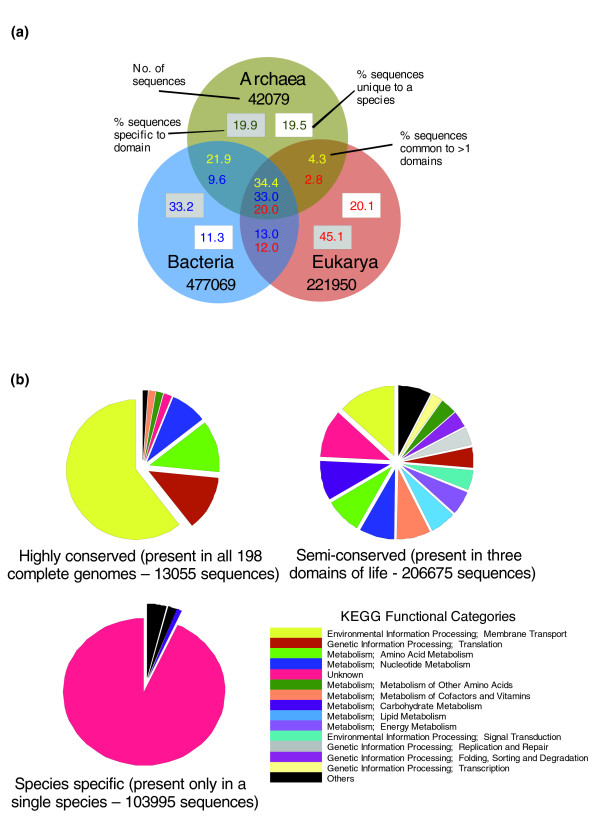
Taxonomic distribution and functional analysis of genes from fully sequenced genomes. On the basis of a raw BLAST score cutoff of 50, we determined the number of sequences with similarity of sequences derived from the three domains of life. **(a) **The Venn diagram shows the proportion of sequences associated with each group. Numbers in grey boxes show the proportion of sequences specific to their parent domain; numbers in white boxes show the proportion of sequences that are shared with one or more members of the same domain. The numbers in the overlapping regions of the diagram show the proportion of sequences shared between the overlapping domains: yellow, archaeal sequences; blue, bacteria; red, eukaryotes. **(b) **Pie charts showing the proportion of each functional category for three datasets of sequences: highly conserved sequences (with sequence similarity to every other complete genome dataset); semi-conserved sequences (with similarity to at least one species from each of the three domains of life); and sequences unique to a genome (possessing no similarity to any other genome dataset). Functional categories were assigned with reference to the KEGG database (see Materials and methods).

It is clear that sequencing of new genomes will continue to reveal a substantial fraction of previously unidentified sequence. We next wished to investigate how non-unique sequences are distributed across the various taxonomic groupings. In this section we use the fully sequenced genome datasets to examine the extent of sequence conservation between the three domains of life (Additional data file 2). Only 20% of eukaryotic sequences are conserved across all three domains (defined as sequences with sequence similarity to at least one bacterial, eukaryotic and archaeal genome), a much lower proportion than for both Archaea and Bacteria (33% and 34.4%, respectively). Conversely, eukaryotes had the highest percentage of domain specific sequences (65.2% compared with 39.4% and 44.5% for Archaea and Bacteria, respectively; Figure 2a). Consistent with our earlier findings, Bacteria possess proportionately fewer (11.3%) species-specific sequences than Eukarya and Archaea (20.1% and 19.5%, respectively).

Within the set of sequences common to all three domains, we may expect to find a core set of 'promiscuous' sequences common to all 198 complete genomes. Previous estimates suggest that there may be as few as 34-80 such genes per genome [15,16,30]. Our analyses identified 13,055 sequences (representing 2% of all sequences from the complete genomes) possessing significant sequence similarity to a sequence from each of the 198 complete genomes. Compared with other less well conserved sequences and consistent with previous findings, these promiscuous sequences are associated with a limited number of basic biological processes, including transcription, translation and metabolism (Figure 2b).

Although we might expect to find similar numbers of promiscuous sequences in each genome, there was considerable variation: from 15 in the nanoarchaeotan *Nanoarchaeum equitans *to 208 in the alphaproteobacterium *Sinorhizobium meliloti *(mean = 64, standard deviation = 33.7). This variation could indicate species-specific expansions associated with one or more of these core genes. Using the COGENT database [31], the 13,055 sequences could be classified into 74 distinct gene families (Additional data file 2). The numbers of gene families per genome (mean = 19.5, standard deviation = 2.6) varied from 13 for *Cryptosporidium parvum *(derived from 16 sequences) to 28 for *Saccharomyces cerevisiae *and *Homo sapiens *(59 and 150 sequences, respectively).

**Figure 3:**
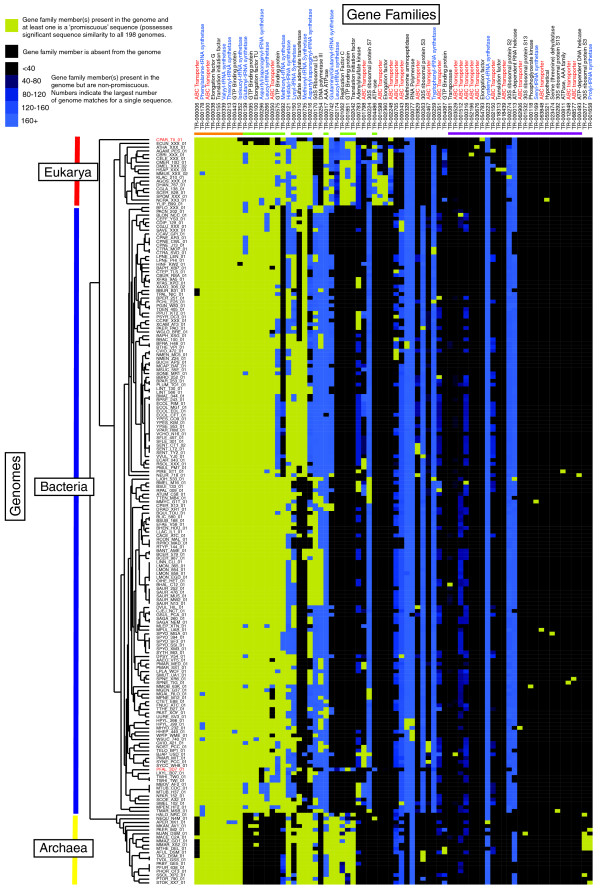
Phylogenetic profile of 74 gene families derived from 'promiscuous' sequences. We identified 13,055 sequences from the complete genome datasets as possessing significant sequence similarity to each of the 198 complete genomes. Gene family assignments obtained from the COGENT database were used to group these promiscuous sequences into 74 gene families. Annotations associated with the gene families show the high incidence of tRNA synthetases (blue text) and ABC transporters (red text). Phylogenetic profiles of each gene family were constructed from the presence or absence of promiscuous sequences in each genome. Two dimensional hierarchical clustering was performed on the profiles using average linkage on the basis of their Spearman rank correlation coefficients. Colored boxes indicate: presence of a promiscuous sequence in the genome (yellow); presence of a non-promiscuous sequence in the genome (blue, shaded according to the number of genomes with which it shares a sequence similarity match - in cases of more than one family member in a genome, the member with the highest number of matches was used); or absence of any family member in the genome (black box). Although the first nine gene families (indicated by the orange bar) contain representatives from the majority of genomes, the remaining gene families demonstrate various levels of specificity. For example, an additional 17 families (light green bars) are common to at least 50% of the eukaryotic genomes while 25 families possessed promiscuous sequences from only a single genome (purple bar). This specificity has led to a clear grouping of genomes into the three domains of life (as indicated on the left of the figure) with the exceptions of *Cryptosporidium parvum *(placed by itself outside the main group of eukaryotes) and *Plasmodium falciparum*, which has been grouped with two strains of *Tropheryma whipplei *and *Leifsonia xyli*. Both species are members of the Apicomplexa, a group of related protist parasites and appear to lack representative sequences from several of the 17 gene families that help define the other eukaryotes as a single group.

The large variation in numbers of promiscuous sequences per genome compared to gene families suggests that, in certain lineages, gene families have undergone significant expansions. For example, of the 208 promiscuous sequences identified in *S. meliloti*, 166 were associated with a single family of ABC transporters. The identification of 74 distinct families with an average of only about 20 families per genome indicates that the Markov clustering (MCL) process used by COGENT may be separating otherwise related sequences into distinct subfamilies on the basis of specialized sequence features. To investigate this further we examined the incidence of other non-promiscuous (that is, with sequence similarity matches to < 198 genomes) members of these 74 families and applied two dimensional clustering to group gene family profiles on the basis of membership of promiscuous sequences (Figure 3). Four groups of families could be identified: those containing promiscuous sequences from a majority of genomes from each of the three domains of life; those containing promiscuous sequences restricted to one or two domains; those containing promiscuous sequences from a limited number of genomes but many non-promiscuous sequences from many other sequences (for example, TR-000223 and TR-000013); and those containing examples of promiscuous (and non-promiscuous) sequences from only a limited number of genomes

The families that contain promiscuous sequences from a majority of genomes from each of the three domains of life include tRNA synthetases (TR-000178, TR000339, TR-000213 and TR-00352), ABC transporters (TR-00006, and TR-000000), elongation factors (TR-000038), translation initiation factors (TR-000155) and GTP binding proteins (TR-000443). These groups may be indicative of a high level of sequence integrity associated with coupling nucleotide binding activity required for their respective functionalities.

Of the families containing promiscuous sequences restricted to one or two domains, 17 are common to at least 50% of the eukaryotic species, 11 are common to at least 50% of Archaeal species, and 9 are common to at least 50% of the bacterial species. These families represent taxa specific subgroups. For example, there are two distinct families of aspartyl, glutaminyl and leucyl synthetases. One set (TR-000216, TR-000742 and TR-002174) is represented in Archaea and Eukarya, while the other (TR-000296, TR-000139 and TR-000266) is represented in Bacteria and Eukarya.

The families containing promiscuous sequences from a limited number of genomes but many non-promiscuous sequences from many other sequences (for example, TR-000223 and TR-000013) may indicate potential gene fusion events or incorrect gene models in which the promiscuous sequences are associated with additional sequence not found in the other members of the family.

Most of the families containing examples of promiscuous (and non-promiscuous) sequences from only a limited number of genomes are representative of sequences that are related to others in the promiscuous sequence dataset (note, for example, the many instances of families of ABC transporters) but which the MCL algorithm has presumably assigned to different families on the basis of distinctive sequence features. Alternatively, promiscuous sequences in these families may possess sequence similarity to sequences outside the set of 13,055 'core' sequences. For example, BLAST analyses of promiscuous sequences derived from *Escherichia coli *reveal that the genes *RBG2*, *RFC2*, *RIX7 *and *RFC3 *do not have significant sequence similarity to any of the 59 promiscuous sequences identified in *S. cerevisiae *(data not shown).

These analyses confirm that COGENT has grouped a number of promiscuous sequences into families on the basis of either domain or species-specific adaptations (groups 2 and 4). Interestingly, there are few examples of families containing promiscuous sequences that are representative of adaptations associated with intermediate taxonomic groups of bacteria (for example, the proteobacteria or spirochaetes). However, further investigations are required to determine if this is biologically meaningful or simply an artifact associated with the sequence clustering algorithm.

### Quantifying sequence diversity within a phylogenetic framework

#### Prokaryotes

**Figure 4:**
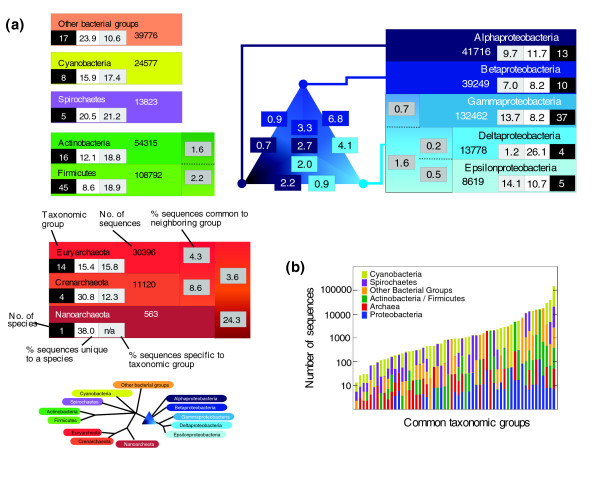
Taxonomic distribution of sequences from prokaryotes. **(a) **On the basis of its phylogenetic profile, each sequence is assigned to a single evolutionary group within their domain. A schematic detailing the phylogenetic relationships of the defined prokaryotic groups is provided in the lower left of the figure. For each taxonomic group the numbers represent: number of genomes analyzed (white text on black); percentage of sequences that are species-specific (black text on white); percentage of sequences that are taxon specific - that is, share sequence similarity only with a sequence(s) from a species from the same taxon (light gray background); and the total number of sequences. Numbers in dark gray boxes indicate the percentage of sequences with similarity to sequence(s) from the neighboring taxon, but not to any other taxon, and may thus represent lineage specific sequences. The numbers in the blue triangle represents the percentage of sequences from each of the three major groups of proteobacteria (alpha, beta and gamma/delta/epsilon) with sequence similarity to each of the other proteobacterial groups). The numbers in the middle of the triangle indicate the percentage of genes from each group (alpha, beta and gamma/delta/epsilon top to bottom) that have sequence similarity to both of the other two groups. **(b) **Bar chart showing the distribution of sequences with sequence similarity to sequences from other bacterial groups, ordered by frequency. Each bar is colored by the groups represented; for example, the first bar from the left indicates the number of sequences from spirochaetes, cyanobacteria and 'other bacterial groups' that have significant sequence similarity to a sequence in each of the other two groups. The largest group, on the right, consists of 145,647 sequences that have similarity to all six prokaryotic groups.

Dividing the prokaryotic genomes into 13 distinct taxonomic groupings (with reference to the National Center for Biotechnology Information's (NCBI) taxonomy resource [32]), comprehensive BLAST comparisons were used to explore sequence diversity within a detailed evolutionary framework (Figure 4). The combined number of taxon-specific (sequences sharing homology only with sequences from at least one other species in the same taxon) and species-specific sequences varied between the 13 taxa from 15.2% (Betaproteobacteria) to 43.1% (Crenarchaeota) with a mean of 30.1%. Taxa with fewer species tended to have a greater number of species-specific sequences. Furthermore, while it might be expected that genomes containing fewer sequences are enriched for more highly conserved sequences (and hence contain fewer species-specific sequences), statistically significant correlation between genome size and the number of species-specific sequences was observed only for the bacterial subdivisions Cyanobacteria and Others (Additional data file 6).

Within the three main proteobacterial divisions (Alphaproteobacteria, Betaproteobacteria and Gamma/Delta/Epsilonproteobacteria) 2-3% of their sequences were common (found in at least one species from each of the three main divisions) and specific to proteobacteria (likely representing core proteobacterial genes). Furthermore, a greater fraction of Betaproteobacterial (6.8%) and Gamma/Delta/Epsilonproteobacterial (4.1%) sequences shared significant similarity with sequences from the other group, compared with the Alphaproteobacteria. Even considering the different sizes of the datasets, these results suggest a closer evolutionary relationship between these first two groups consistent with previous findings [28].

Within Archaea, a large fraction of sequences was found to be common and specific to the various archaeal groups. For example, 8.6% of sequences associated with Crenarchaeota are specific and common across the Euryacheaota/Crenarchaeota lineage, while 24.3% of Nanoarchaeota genes share sequence similarity only with other Archaea. This suggests a common core of archaeal specific sequences and demonstrates the divergence between archaea and bacteria.

Due to the lack of a robustly defined bacterial phylogeny, rather than attempt to map the remaining sequences common across deeper taxonomic groups, we analyzed the occurrence of sequences with similarity to sequences from one or more additional taxa (Figure 4b). The largest group of sequences (145,647; 31% of the prokaryotic sequences analyzed in this study) was found to be common across all six prokaryotic groups, representing either a core set of housekeeping genes that have arisen through a common ancestor or sequences that may be highly prone to LGT [18,33-35]. The next largest category (37,700; 8%) consist of sequences that are common to all five categories of bacteria but absent in Archaea. These might represent common ancestral bacterial sequences that have been lost within Archaea and are not readily acquired by these organisms through LGT.

#### Eukaryotes

**Figure 5:**
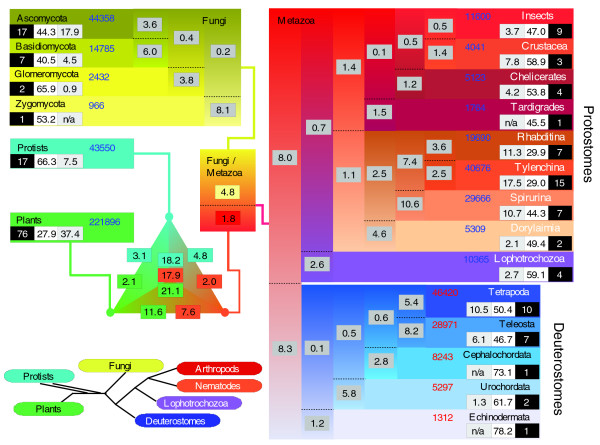
Taxonomic distribution of sequences from eukaryotic partial genomes. As for Figure 4a, this graphic presents the distribution of partial genome sequences associated with 20 eukaryotic taxa. For each taxon, the three numbers in boxes represent: number of species in group (white numerals on black background); percentage of sequences that are species-specific (that is, do not share any sequence similarity with any other species; black numerals on white background); percentage of sequences that are group specific (that is, share sequence similarity only with one or more sequences from a species in the same taxon (light gray background). The numbers of sequences in each group are given in blue (orange for deuterostomes). Numbers in dark gray boxes indicate the percentage of sequences from each group that have sequence similarity to a gene from the corresponding group. Note that this figure does not attempt to resolve the root of eukaryotes but, as for Figure 4a, has a triangular graphic to represent connections between the three major taxonomic groups - protists, plants and fungi/metazoa. Similarly, as for Figure 4a, this graphic does not provide explicit information on relative times of divergence and care must be taken in comparing numbers between different branches. For example, one study based on molecular clock analysis has estimated the time of divergence of ascomycetes and basidiomycetes to range from 850 to 1,100 Mya, similar to that of protostomes and deuterostomes (880-1,080 Mya) [37]. In this figure, the former might appear to be a relatively recent split while the latter (due to its more basal position within the tree) might appear to be more ancient.

Due to the limited number and lack of diversity associated with complete genomes, we chose to exploit the large number of partial genomes to perform a similar mapping of eukaryotic sequences within a phylogenetic framework. Partial genomes were divided into 20 distinct taxa and comprehensive BLAST analyses were again used to compare sequences within a phylogenetic framework (Figure 5; see Materials and methods for further descriptions of taxonomic groups and their relationships). While the taxa used in this study are typical of studies of molecular evolution in eukaryotes, it should be appreciated that there is considerable variation in the number of species and sequences associated with each taxon. In addition, while ideally we would like to place these analyses in the context of relative times of divergence, there is a great deal of uncertainty concerning predictions of evolutionary divergence events. For example, estimated divergence times between protostomes and deuterostomes range from 570-1,100 million years (Mya) [36-39] and for chelicerates and pancrustacea (which include insects and crustaceans) from 540-770 Mya [36,39].

Consequently, the cladograms presented in Figures 4a and 5 do not provide explicit information on relative times of divergence and caution should, therefore, be exercised in interpreting comparisons between the various branches.

Consistent with findings from the fully sequenced eukaryotes, the partial genome datasets contain a larger proportion (approximately 40-70%) of taxon- and species-specific sequences than the prokaryotic datasets. As might be expected, taxa with larger numbers of species contained the highest proportion of conserved taxon-specific sequences (that is, sequences specific to a taxon but common to > 1 species): plants (37.4%, 76 species); ascomycetes (17.9%, 17 species) and tylenchids (17.5%, 15 species). Comparisons between taxa revealed several branches that contain a relatively large proportion of common sequences. Within the nematodes, for example, 10.6% of the 29,666 spirurid sequences have similarity only to rhabditid and/or tylenchid sequences, while reciprocally, 7.4% of the 60,366 rhabditid and tylenchid sequences are also common only to spirurids (the difference in percentages is likely due to the different sizes of the respective datasets). Similarly, 6% of the 14,785 basidiomycete sequences and 3.6% of the 44,358 ascomycete sequences are specific to basidiomycetes and ascomycetes, while 5.4% of 46,420 tetrapod sequences and 8.2% of 28,971 teleost sequences are common and specific to tetrapods and teleosts. These sequences potentially represent core sets of essential nematode, fungi and vertebrate specific genes that arose relatively early in their respective lineages.

Interestingly, despite comparable sequence and gene family discovery rates (Table 2), the arthropod taxa displayed much lower proportions of panarthropod core sequences than the nematode, fungal and deuterostome lineages. For example, only 0.5% of 11,600 insect sequences and 1.4% of 4,041 crustacean sequences are specific to pancrustacea. Although this might indicate a relatively rapid divergence of the three lineages after their ancestral split from the nematodes, these findings might also be readily explained by the lower number of available sequences associated with these taxa. Additional sequencing within the arthropods may, therefore, increase the number of such common sequences with a corresponding decrease in the proportion of species and taxon-specific sequences associated with these groups.

Many sequences are also associated with deeper taxonomic splits within Eukarya. Of particular note, approximately 8% of metazoan sequences are common and specific to the protostomes and deuterostomes. These likely represent sequences involved in providing basic multicellular functionality (for example, cell-cell communication and differentiation). Additionally, 1.8% of metazoan sequences and 4.8% of fungal sequences are common to the fungi/metazoan divide. Again, these sequences may represent specific adaptations adopted by the early ancestors of the opistokonts. Since the root of the tree connecting protists, plants and fungi/metazoa has not been well defined, we analyzed each combination separately. Approximately 18-21% of eukaryotic sequences are common to each of the three major eukaryotic taxonomic groups. These sequences are expected to be involved in basic housekeeping functions, such as DNA processing and cellular metabolism.

Comparisons with the prokaryotic datasets revealed that sequences furthest from the root of eukaryotes were less likely to share similarity with a prokaryotic sequence. For example, 58.8% (60,485) of the 102,868 core eukaryotic sequences, 29.4% (1,637) of the 5,573 Fungi-Metazoa specific sequences, and only 14.2% (2,196) of the 15,486 Metazoa specific sequences shared similarity with a prokaryotic sequence. Furthermore, for the majority (135 of 193) of the species-specific datasets, less than 2% of their sequences had significant matches to a prokaryotic sequence. The incidence of a small fraction of these sequences sharing similarity with prokaryotic sequences may reflect a low incidence sequence acquisition through LGT [40].

While the use of partial genomes offers a breadth and depth of sequence sampling unrivalled by full genomes, potential drawbacks of these datasets have been documented [14,17,41]. Indeed, we note that in comparing sequence length with conservation, longer sequences tend to be more highly conserved (Additional data file 7). However, previous reports supporting the legitimacy of short sequences [14,41], together with the consistency in the proportion of species-specific sequences with the fully sequenced eukaryotic datasets, highlights the usefulness of sequences derived from partial genomes as surrogates for those derived from complete genomes.

### Functional analysis of highly conserved eukaryotic sequences

**Figure 6:**
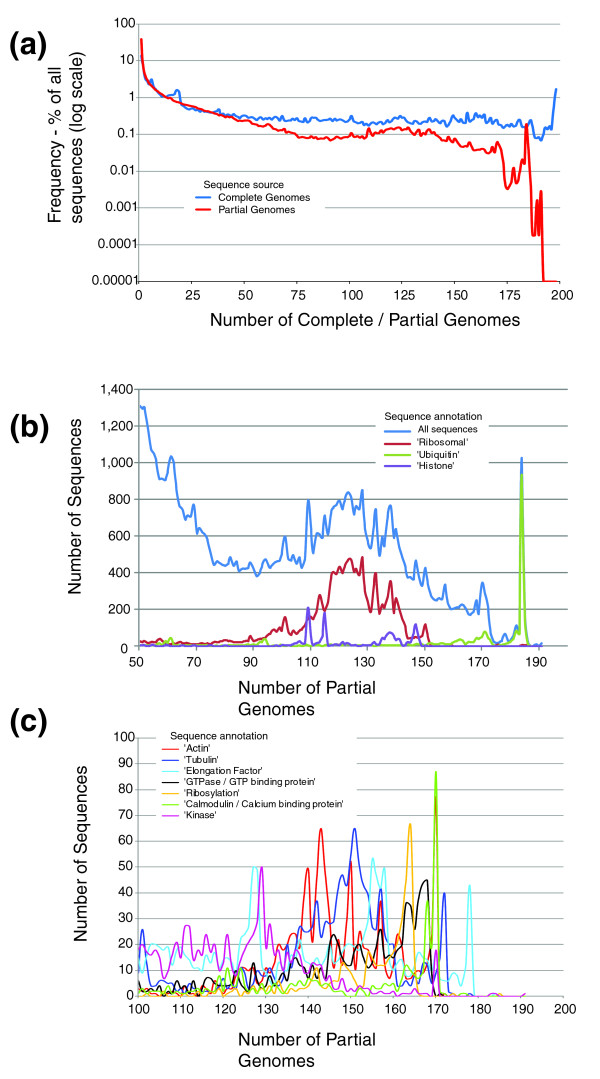
Distribution of conservation of sequences from full and partial genomes and functional characterization. **(a) **The frequency of sequences from full and partial genomes with significant sequence similarity to other full or partial genomes. Most sequences are associated with only a limited number of genomes; however, two peaks on the respective graphs indicate that there is a large proportion of sequences from full and partial genomes that have similarity to sequences from 198 and 185 genomes and partial genomes, respectively. **(b, c) **The number of partial genome sequences with specific BLAST annotations that are conserved across either 50-191 (b) or 100-191 (c) other partial genomes (see Materials and methods for more details).

The sequence datasets reported here are provided as a community resource through interactive images available online [27]. To demonstrate the utility of these datasets, we undertook a functional analysis of the more highly conserved sequences from the eukaryotic partial genome datasets (Figure 6). Comparing the frequency of sequences with similarity to sequences from other genomes (Figure 6a) reveals that sequences from partial genomes display an atypical abundance ('hump') of sequences with similarities to between 110 and 140 other partial genomes, compared with sequences derived from the full genome datasets. Analysis of BLAST annotations derived with reference to the non-redundant protein database revealed that this hump is associated with an abundance of 'ribosomal' proteins (Figure 6b), indicative of both their conserved nature and the large number of such sequences produced in EST sequencing projects. The peak at 182 genomes was associated with sequences associated with the term 'ubiquitin' while other annotations for abundant conserved sequences in these datasets include 'histone', 'cyclophilin', 'elongation factor', 'actin' and 'tubulin' (Figure 6c). These all represent classes of genes commonly associated with EST sequencing projects due to their high expression levels.

## Discussion

Recently there has been considerable interest in exploring the generation and extent of genetic diversity [4,5,20]. Due to the lack of fully sequenced genomes available for eukaryotes, such studies have tended to focus on prokaryotes. Here we have supplemented 198 fully sequenced genomes (19 from eukaryotes) with 193 EST derived datasets (partial genomes) to undertake a comprehensive global analysis of sequence diversity. Our results indicate that eukaryotic (partial genome) datasets tend to possess a higher proportion of taxon- and species-specific sequences in addition to demonstrating a higher rate of novel sequence discovery than prokaryotes. While this reduction in diversity may be associated with biases in the choice of bacterial genomes for sequencing (for example, due to medical and/or economical interests), such biases could only partially account for the observed differences. It is also interesting to note that a recent metagenomics project, focusing on the collation of global ocean samples and hence not subject to the same sampling biases, reported that 14% of sequence reads obtained from a single site were unique to that site [9,42]. Despite methodological differences, the consistency in these rates provides an additional level of support that biases in genome sampling have not adversely influenced our results.

A major driving force of sequence diversity is thought to involve the duplication of genes followed by their subsequent divergence [2,12]. The observed differences between the bacterial and partial genome datasets may, therefore, be driven by factors such as generation times and metabolic rates [43]. However, another potentially important factor is the role played by LGT and its ability to transfer new genes (for example, created through duplication and divergence) across species [18,33-35,44]. Recent interest in LGT events has led to the concept of a microbial pan-genome consisting of a global pool of genes that are continuously being exchanged within and between prokaryotic species [45,46]. This constant exchange would obviously decrease the number of species-specific genes relative to those species that mainly rely on vertical transmission of genetic information (that is, eukaryotes). Intriguingly, if LGT is a major source of gene acquisition, the identification of 'core' sequences limited to specific prokaryotic taxa (for example, Proteobacteria) may indicate sets of sequences not readily transferred through LGT. The provision of these sequence datasets will, therefore, facilitate future studies exploring the relationship between their sequence and/or functional properties and their restriction to specific lineages.

Focusing on eukaryotes, by mapping their sequences onto a phylogenetic framework, we identified a widely populated spectrum of sequence specificity. At one extreme approximately 20% of eukaryotic sequences are highly conserved and may represent ancestral eukaryotic genes under significant selective constraints. At the other extreme, from 40-60% of sequences are specific to individual or closely related species. Such sequences represent genes that are either under reduced selective constraints, provide newly acquired functionality (that is, neo- or sub-functionalization [47]) or are simply redundant and in the process of being lost. Between these two extremes several regions ('landmarks') within the phylogenetic landscape were identified as demonstrating high levels of sequence conservation (for example, the 8% of metazoan sequences that are specific and conserved across protostomes and deuterostomes). In addition to ancestral genes that may have been lost in other lineages, these sequences represent newly evolved genes subject, as they arise, to selective constraints restricting further diversification. An intriguing question concerns whether these landmarks represent 'bursts' of concerted gene innovations. For example, sequences mediating cell adhesion or cell-cell communication would promote a multi-cellular lifestyle that may have resulted in the rapid generation of metazoan specific sequences) [48]. As for the highly conserved sequences, subsequent diversification of these sequences may be limited by these altered constraints. Alternatively, depending upon the relative times of divergence, these landmarks may simply reflect extended periods of evolution allowing the continued accumulation of sequences prior to a divergence event. For example, the relatively high proportion of metazoan specific sequences (8%) may simply reflect an extended period, from the divergence of fungi from metazoan to the divergence of protostomes and deuterostomes (which one estimate puts at 500 Mya [37]). Relative divergence times might also account for the relatively low numbers of sequences shared across the three major groups of arthropods. Molecular clocks estimate the time of divergence of insects and crustacea to be approximately 650 Mya and that of panarthropods from chelicerates as approximately 720 Mya [39]. Hence, it may not be surprising that during the 70 Mya between these two splits, only 0.5% of insect sequences and 1.4% of crustacean sequences were generated and remain common and specific to these two taxa (compare with the 300 Mya between the time of divergence of the tetrapods and teleosts and their earlier divergence from the cephalochordates [49]). On the other hand, as noted earlier, factors such as generation time and metabolic rates in addition to significant changes in population size could also play a role in the observed increases in rates of sequence innovation [43,50,51].

With current ambiguities in the timing of divergence events [52], interpretation of these data would greatly benefit from the availability of a fully resolved and robustly timed phylogeny. Conversely, these data may be usefully combined with additional experimental and theoretical studies to unravel the relative influence of these various factors on the generation of sequence diversity within the Eukarya [53,54].

## Conclusion

The collation and comparison of EST based datasets from 193 species provides the first detailed analysis of sequence conservation across Eukarya and highlights significant differences from prokaryotes. In particular, we find that eukaryotes have a much higher incidence of novel sequences than prokaryotes, which may be related to the lower incidence of LGT events. Placing these sequences within a phylogenetic framework provides a detailed map of the origins and extent of sequence diversity. It further allows the identification of sequences specific and conserved to distinct taxonomic groups that are likely to be associated with novel taxon specific innovations. The provision of taxon specific sequences should thus prove valuable for additional computational and biochemical analyses aimed at understanding evolutionary and functional relationships.

## Materials and methods

### Sequence data

The predicted protein sequences of 198 complete genomes used in this study were obtained from the COGENT database [31]. Sequences associated with 193 partial genomes were obtained from the PartigeneDB database [26]. A list of all species datasets is given in Additional data files 2 and 3. A summary of the taxonomic groups represented in this study is shown in Table 1. Detailed taxonomic relationships of the organisms are presented in Additional data file 1. Species were assigned to each group on the basis of taxonomic information derived from the NCBI TaxBrowser resource [55]. Due to ambiguity concerning the phylogenetic relationships of bacteria [56], no attempt is made to provide an evolutionary perspective beyond the major bacterial groups (proteobacteria, actinobacteria/firmicutes, cyanobacteria, spirochaetes and 'other bacteria' - encompassing species that do not fall in one of the aforementioned groups). Archaeal relationships were inferred with reference to [57]. Eukaryotic relationships were inferred from a number of previously published studies as follows: while the relationships between Fungi, Metazoa and Viridiplantae have been well established, protests are thought to derive from several paraphyletic groups arising at (or close to) the root of the three other major eukaryotic groups [29]. Due to the limited number of partial genomes from this group and to simplify analyses, the 17 protist partial genomes were collated into a single group. For fungi (four taxonomic groups - Ascomycota, Basidiomycota, Glomeromycota and Zygomycota), nematodes (four taxa - Rhabditina, Tylenchina, Spirurina and Dorylaimia) and arthropods/tardigrades (four taxa - Hexapoda, Crustacea, Chelicerata and Tardigrada) the taxonomic divisions and phylogenetic relationships were defined according to previous, established studies [58-60]. Deuterostomes may be readily categorised into five well-defined taxa - Tetrapoda, Teleosta, Cephalochordata, Urochordata and Echinodermata. However, the phylogenetic relationships of the cephalochordates and urochordates with respect to the other three groups remain unclear. For the purposes of our analyses, we assumed relationships based on previously published studies of ribosomal RNA analyses [61,62]. Finally, there has been much debate concerning the grouping of the major metazoan clades [63-65]. We have chosen to follow the scheme proposed by a recent study performed by Philippe and co-workers [64]. In their analysis of a large dataset of 146 genes derived from a diverse set of 35 animals, they provide strong support for arthropods and nematodes belonging to the protostome group Ecdysozoa. The protostomes (which also include Lophotrochozoans such as mollusks and platyhelminths) then form a group distinct from the deuterostomes, which include the chordates and hemichordates (the so-called Lophotrochozoa-Ecdysozoa-Deuterostomia (L-E-D) hypothesis [66]).

### BLAST analyses

Given the scale of sequence similarity searches, BLAST [67] provides the most practical tool for performing these types of analyses. Unlike more sensitive algorithms such as PSI-BLAST, it is both fast and easily automated, allowing the comparison of the hundreds of datasets considered here. For each sequence from each complete and partial genome, a series of BLASTs were performed. The predicted proteomes from each complete genome were compared to each other using BLASTP, while sequences from partial genomes were compared using TBLASTX. Raw bit scores were extracted and stored within an in-house PostgreSQL based database [68]. This results in the creation of a phylogenetic profile [69] for each sequence from which its relationship to various taxonomic groups can be derived. Significant matches were defined as those having a raw BLAST score of greater or equal to 50 (equivalent to a typical E-score of about 10^-5^). Previous studies have concluded that the choice of threshold does not significantly affect qualitative findings in these types of analyses [15,17]. We therefore chose an intermediate threshold that minimizes erroneous matches while maximizing the possibility of identifying related sequences. A full list of species, their taxonomic group, numbers of sequences, fraction of species-specific sequences and fraction of sequences specific to a limited number of taxonomic groups are provided in Additional data files 2 and 3. For the analyses of sequence discovery rates, as genomes are included in a cumulative sequence ensemble (that is, sampled), we identify 'distinct' sequences as those that do not possess significant sequence similarity to sequences from genomes that have already been sampled. A sliding window of 30 genomes was then used to obtain the gradient of the number of distinct sequences compared against the number of sequences that have been sampled. Due to the use of this sliding window, for each dataset, values were not obtained for the first 15 or last 15 genome additions. Four hundred random orderings of genome additions were generated to derive the average and standard deviation of sequence discovery rates for each dataset.

### Gene family analyses

Gene family predictions for the 198 complete genome datasets obtained from COGENT have previously been calculated through the use of the TribeMCL algorithm [70]. In order to obtain the predictions for the partial genome datasets, which consist of DNA as opposed to protein sequences, it was first necessary to calibrate the parameters (inflation values and E-value cutoffs) used by the TribeMCL algorithm to maintain consistency with the COGENT analyses. We therefore obtained the cDNA and associated peptide sequences for *C. elegans *from Wormbase [71] (Wormpep version 160). After clustering these peptide sequences by TribeMCL using similar parameters used to construct the COGENT gene family predictions (E-value cutoff = 0.001; inflation value = 1.86), a comprehensive scan of parameters was performed for clustering the cDNA sequences. An E-value cutoff of 0.001 and an inflation value of 1.635 were found to provide the most similar number of gene families in the cDNA based families compared with the peptide based families (11,020 versus 11,018, respectively). These parameters were used to cluster the 546,451 partial genome sequences and derive a single set of gene family predictions that encompass all of the partial genomes. Gene family discovery rates were obtained as for the sequence discovery rates - as genomes are sampled, we identify new gene families (including singletons) as those that do not include sequence members from genomes that have already been sampled. Again, we used a sliding window of 30 genomes to obtain the gradient of the number of gene families compared against the total number of sampled sequences. Furthermore, four hundred random orderings of genome additions were generated to derive the average and standard deviation of gene family discovery rates for each dataset.

### Functional characterization

Two sets of functional annotations were used in this study. In the first set, broad functional characterizations were obtained for sequences in both the complete and partial genome datasets with reference to the Kyoto Encyclopedia of Genes and Genomes (KEGG) database (release 32.0; 748,177 protein sequences from 206 genomes) [72]. All sequences were assigned to a functional category according to the sequence in KEGG with the highest sequence similarity (BLAST raw score cut-off of ≥ 50). Sequences that did not have significant sequence similarity to a protein in KEGG with a previously assigned functional category were characterized as 'Unknown'. The second set of annotations used in this study, providing more detail on specific gene functions, was applied only to sequences from the partial genome datasets. In brief, each sequence was subjected to a BLAST search against the protein non-redundant database (2.5 million sequences). Up to five matches with E-values < 10^-5 ^were extracted. Meaningful annotations were then extracted from the descriptions of the first 'hit' that did not contain 'hypothetical', 'unknown' or 'unnamed'.

## Abbreviations

CGDR, current gene family discovery rate; CSDR, current sequence discovery rate; EST, expressed sequence tag; KEGG, Kyoto Encyclopedia of Genes and Genomes; LGT, lateral gene transfer; MCL, Markov clustering; Mya, millions of years; NCBI, National Center for Biotechnology Information; OGDR, overall gene family discovery rate; OSDR, overall sequence discovery rate.

## Authors' contributions

JP and JMP-A conceived and designed the experiments. JMP-A collated sequence datasets, designed and performed the cross-species and gene family analyses and helped draft the manuscript. JP undertook the functional characterization analyses, wrote the manuscript and supervised the project. Both authors read and approved the final manuscript.

## Additional data files

The following additional data are available with the online version of this paper. Additional data file 1 is a figure showing the phylogenetic relationships of the taxonomic groups discussed in the main text. Additional data files 2 and 3 are tables listing the species used in the study together with a detailed breakdown of the taxonomic relationships of their sequences. Additional data file 4 is a figure comparing sequence similarity relationships between members of 16 highly conserved gene families for eukaryotes and bacteria. Additional data file 5 is a figure showing rates of sequence and gene family discovery in different sets of complete and partial genomes. Additional data file 6 is a figure showing the relationship between genome size and number of species-specific sequences. Additional data file 7 is a figure showing the relationship between the sequence conservation and length.

## Supplementary Material

Additional data file 1 Phylogenetic relationships of the taxonomic groups discussed in the main text. Click here for file

Additional data file 2 Species used in the study together with a detailed breakdown of the taxonomic relationships of their sequences. Click here for file

Additional data file 3 Species used in the study together with a detailed breakdown of the taxonomic relationships of their sequences. Click here for file

Additional data file 4 Comparison of sequence similarity relationships between members of 16 highly conserved gene families for eukaryotes and bacteria. Click here for file

Additional data file 5 Rates of sequence and gene family discovery in different sets of complete and partial genomes. Click here for file

Additional data file 6 Relationship between genome size and number of species-specific sequences. Click here for file

Additional data file 7 Relationship between sequence conservation and length. Click here for file
