# Supplementary material for: The global landscape of sequence diversity
Source: Genome Biol. 2007 Nov 8;8(11):R238. doi: 10.1186/gb-2007-8-11-r238 (PMC2258180; doi:10.1186/gb-2007-8-11-r238)
Supplement: Additional data file 1 — Phylogenetic relationships of the taxonomic groups discussed in the main text. [file gb-2007-8-11-r238-S1.pdf]

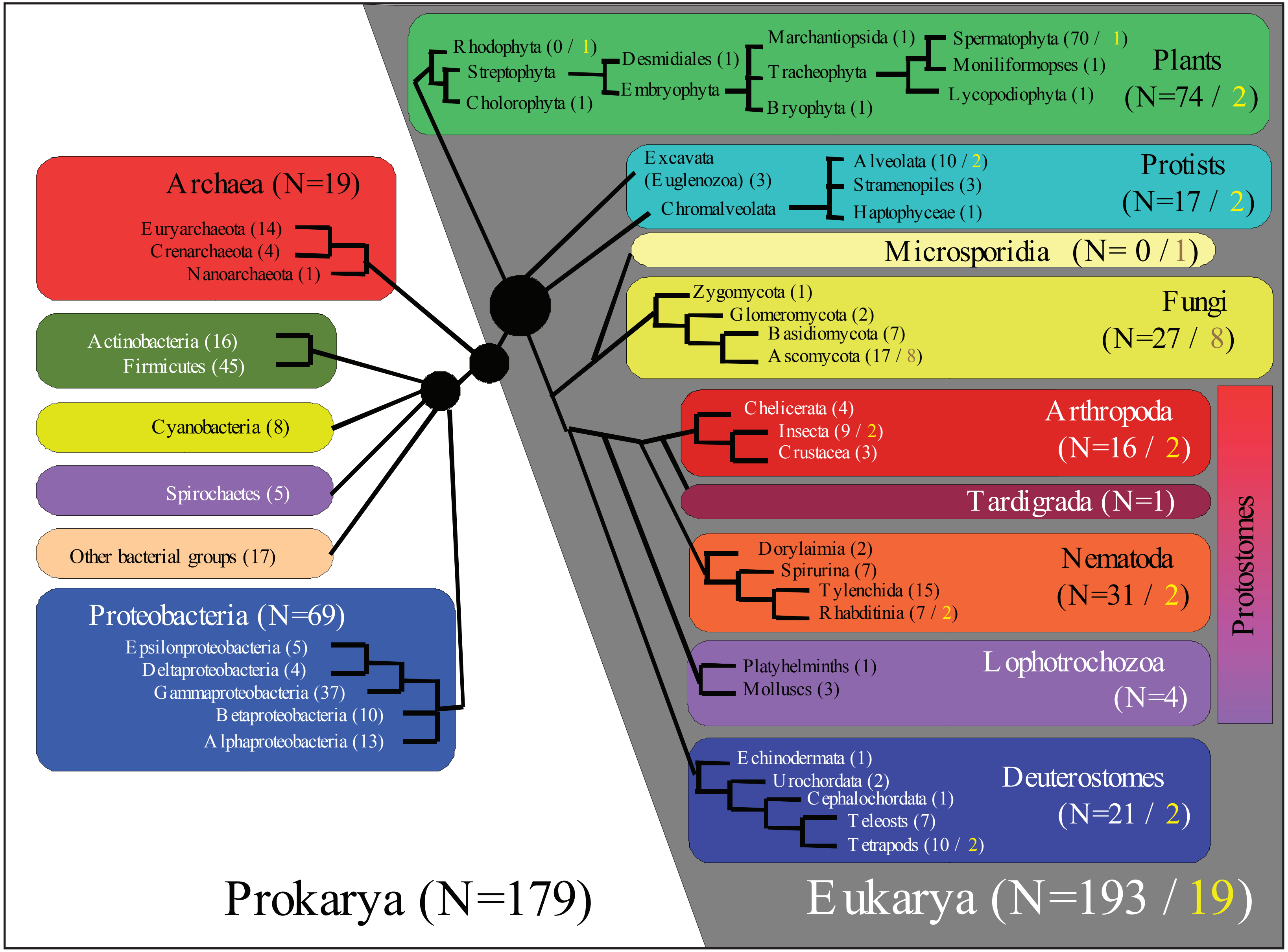

Figure S1 Taxonomic organization of the groups analysed in this study.
The tree shows the assumed evolutionary relationships used in the study. Organisms were split into two major groups: Prokaryotes (white background) and Eukaryotes (black background). The large black circles indicate major partitions where evolutionary relationships remain unresolved. These include the division between: Archaea, Bacteria and Eukarya; the five bacterial phyla – Proteobacteria, Actinobacteria/Firmicutes, Cyanobacteria, Spirochaetes and other bacterial groups; and the three major eukaryotic groups – Plants, Protists and Fungi/Metazoa. Note that 'Protists' are an artificial group since they contain the polyphyletic Excavates and Chromalveolates. The numbers for the prokaryotes indicate the number of full genomes used in this study associated with each group. The two numbers for the eukaryotic groups indicate the number of partial and full genomes respectively, used in this study. Phylogenetic relationships were derived as stated in Methods.
