# Supplementary material for: The global landscape of sequence diversity
Source: Genome Biol. 2007 Nov 8;8(11):R238. doi: 10.1186/gb-2007-8-11-r238 (PMC2258180; doi:10.1186/gb-2007-8-11-r238)
Supplement: Additional data file 2 — Species used in the study together with a detailed breakdown of the taxonomic relationships of their sequences. [file gb-2007-8-11-r238-S2.doc]

**Table S1.** List of full sequenced genomes analysed in the study obtained from the COGENT database. Species are ordered by major taxonomic groups (Archaea; Bacteria; Eukaryota). For each species the number of sequences associated with the genome are given, together with the percentage of genes shared between the major taxonomic groupings (according to BLAST similarity scores – see methods). A = Archaea; B = Bacteria; E = Eukarya, hence 21.34% of sequences associated with *Aeropyrum pernix* have significant similarity to sequences from all three domains of life; while 10.36% of sequences share similarity to other Archaea and Bacteria but not to Eukarya. Note in some cases, there are no matches to other members of the same domain – e.g. 0.97% of *Aeropyrum pernix* sequences shared similarity with one or more sequences from Bacteria but not to another Archaea. SS (%) and SS (#) indicates the percentage and numbers of species specific sequences i.e. sequences which did not share similarity with a sequence from any other genome, respectively. UB (#) indicates the number of “ubiquitous sequences” i.e. those with significant sequence similarity to sequences in every other fully sequenced genome. GF of SS (#) and GF of UB (#) indicate the number of gene families associated with the species specific and ubiquitous sequences respectively as determined through the COGENT database. SS Dup (%) and UB Dup (%) indicate the frequency of putative duplication events in the species specific and ubiquitous sequences respectively. These were calculated by comparing the number of sequences and the number of gene families for each dataset using the following: SS Dup(%) = 100 x (1 – (GF of SS(#) / SS(#))) and UB Dup(%) = 100 x (1 – (GF of UB(#) / UB(#))) respectively.

| *Taxon* | *Domain* | *Species* | *COGENES ID* | *Genes* | *ABE%* | *BE%* | *AB%* | *AE%* | *A%* | *B%* | *E%* | *SS (%)* | *SS (#)* | *GF of SS (#)* | *SS Dup (%)* | *UB (#)* | *GF of UB (#)* | *UB Dup (%)* |
| --- | --- | --- | --- | --- | --- | --- | --- | --- | --- | --- | --- | --- | --- | --- | --- | --- | --- | --- |
| *Crenarchaeota* | Archaea | *Aeropyrum pernix* | APER-XK1-01 | 2694 | 21.34 | 0.30 | 10.36 | 2.90 | 9.17 | 0.97 | 0.04 | 54.94 | 1480 | 1476 | 0.27 | 47 | 17 | 63.83 |
| *Crenarchaeota* | Archaea | *Pyrobaculum aerophilum* | PAER-IM2-01 | 2605 | 26.18 | 0.19 | 15.39 | 2.96 | 11.79 | 1.27 | 0.04 | 42.19 | 1097 | 938 | 14.49 | 44 | 17 | 61.36 |
| *Crenarchaeota* | Archaea | *Sulfolobus solfataricus* | SSOL-XP2-01 | 2995 | 33.86 | 0.23 | 19.77 | 3.04 | 30.75 | 0.80 | 0.00 | 11.55 | 346 | 237 | 31.50 | 56 | 19 | 66.07 |
| *Crenarchaeota* | Archaea | *Sulfolobus tokodaii* | STOK-XX7-01 | 2826 | 31.63 | 0.14 | 16.84 | 3.11 | 30.15 | 0.35 | 0.04 | 17.73 | 501 | 470 | 6.19 | 49 | 20 | 59.18 |
| *Euryarchaeota* | Archaea | *Archaeoglobus fulgidus* | AFUL-DSM-01 | 2409 | 34.25 | 0.25 | 23.37 | 3.53 | 15.15 | 2.74 | 0.17 | 20.55 | 495 | 474 | 4.24 | 56 | 21 | 62.50 |
| *Euryarchaeota* | Archaea | *Halobacterium sp.* | HALO-NRC-01 | 2605 | 28.56 | 2.15 | 15.66 | 3.07 | 8.83 | 5.91 | 0.19 | 35.62 | 928 | 807 | 13.04 | 41 | 17 | 58.54 |
| *Euryarchaeota* | Archaea | *Methanobacterium thermoautotrophicum* | MTHE-DEL-01 | 1871 | 34.05 | 0.37 | 24.37 | 4.22 | 16.09 | 2.19 | 0.00 | 18.71 | 350 | 333 | 4.86 | 34 | 21 | 38.24 |
| *Euryarchaeota* | Archaea | *Methanococcus jannaschii* | MJAN-DSM-01 | 1773 | 32.32 | 0.28 | 23.58 | 4.29 | 23.58 | 1.18 | 0.11 | 14.66 | 260 | 249 | 4.23 | 36 | 21 | 41.67 |
| *Euryarchaeota* | Archaea | *Methanococcus maripaludis* | MMAR-XS2-01 | 1722 | 37.80 | 0.87 | 24.68 | 4.36 | 20.91 | 2.38 | 0.06 | 8.94 | 154 | 148 | 3.90 | 38 | 21 | 44.74 |
| *Euryarchaeota* | Archaea | *Methanopyrus kandleri* | MKAN-AV1-01 | 1687 | 28.63 | 0.12 | 19.50 | 4.80 | 15.65 | 1.54 | 0.12 | 29.64 | 500 | 462 | 7.60 | 27 | 18 | 33.33 |
| *Euryarchaeota* | Archaea | *Methanosarcina acetivorans* | MACE-C2A-01 | 4528 | 31.03 | 0.57 | 26.68 | 1.90 | 20.25 | 3.07 | 0.13 | 16.36 | 741 | 681 | 8.10 | 102 | 23 | 77.45 |
| *Euryarchaeota* | Archaea | *Methanosarcina mazei* | MMAZ-GO1-01 | 3371 | 33.61 | 0.27 | 28.36 | 2.67 | 25.22 | 1.96 | 0.15 | 7.77 | 262 | 249 | 4.96 | 63 | 21 | 66.67 |
| *Euryarchaeota* | Archaea | *Picrophilus torridus* | PTOR-790-01 | 1535 | 46.91 | 0.26 | 18.44 | 4.56 | 19.48 | 0.85 | 0.13 | 9.38 | 143 | 140 | 2.10 | 39 | 17 | 56.41 |
| *Euryarchaeota* | Archaea | *Pyrococcus abyssi* | PABY-GE5-01 | 1765 | 41.02 | 0.06 | 24.76 | 4.65 | 26.40 | 0.57 | 0.00 | 2.55 | 45 | 45 | 0.00 | 46 | 18 | 60.87 |
| *Euryarchaeota* | Archaea | *Pyrococcus furiosus* | PFUR-638-01 | 2065 | 37.29 | 0.15 | 24.16 | 4.36 | 24.84 | 0.82 | 0.00 | 8.38 | 173 | 169 | 2.31 | 62 | 18 | 70.97 |
| *Euryarchaeota* | Archaea | *Pyrococcus horikoshii* | PHOR-OT3-01 | 2061 | 32.46 | 0.10 | 19.99 | 3.93 | 23.68 | 0.34 | 0.00 | 19.51 | 402 | 401 | 0.25 | 47 | 18 | 61.70 |
| *Euryarchaeota* | Archaea | *Thermoplasma acidophilum* | TACI-DSM-01 | 1478 | 45.20 | 0.07 | 21.31 | 5.01 | 21.99 | 0.68 | 0.00 | 5.75 | 85 | 84 | 1.18 | 41 | 18 | 56.10 |
| *Euryarchaeota* | Archaea | *Thermoplasma volcanium* | TVOL-GSS-01 | 1526 | 43.38 | 0.07 | 20.64 | 4.91 | 22.41 | 0.79 | 0.00 | 7.80 | 119 | 117 | 1.68 | 35 | 18 | 48.57 |
| *Nanoarcheota* | Archaea | *Nanoarchaeum equitans* | NEQU-N4M-01 | 563 | 27.89 | 0.18 | 9.24 | 11.72 | 12.61 | 0.53 | 0.18 | 37.66 | 212 | 212 | 0.00 | 15 | 15 | 0.00 |
|  |  |  |  |  |  |  |  |  |  |  |  |  |  |  |  |  |  |  |
| *Alphaproteobacteria* | Bacteria | *Agrobacterium tumefaciens* | ATUM-C58-01 | 5299 | 36.99 | 11.53 | 10.87 | 0.00 | 0.04 | 30.01 | 0.00 | 10.57 | 560 | 546 | 2.50 | 198 | 22 | 88.89 |
| *Alphaproteobacteria* | Bacteria | *Bartonella henselae* | BHEN-HOU-01 | 1488 | 33.06 | 15.26 | 6.79 | 0.00 | 0.00 | 35.01 | 0.00 | 9.88 | 147 | 96 | 34.69 | 47 | 19 | 59.57 |
| *Alphaproteobacteria* | Bacteria | *Bartonella quintana* | BQUI-TOU-01 | 1142 | 39.75 | 18.48 | 7.18 | 0.00 | 0.00 | 33.19 | 0.00 | 1.40 | 16 | 16 | 0.00 | 44 | 18 | 59.09 |
| *Alphaproteobacteria* | Bacteria | *Bradyrhizobium japonicum* | BJAP-USD-01 | 8317 | 31.54 | 11.43 | 11.69 | 0.01 | 0.05 | 30.26 | 0.07 | 14.95 | 1243 | 1194 | 3.94 | 181 | 21 | 88.40 |
| *Alphaproteobacteria* | Bacteria | *Brucella melitensis* | BMEL-M16-01 | 3198 | 36.71 | 13.20 | 10.23 | 0.00 | 0.00 | 36.68 | 0.00 | 3.19 | 102 | 101 | 0.98 | 104 | 22 | 78.85 |
| *Alphaproteobacteria* | Bacteria | *Brucella suis* | BSUI-133-01 | 3293 | 34.10 | 12.12 | 9.35 | 0.00 | 0.00 | 33.19 | 0.00 | 11.24 | 370 | 354 | 4.32 | 98 | 21 | 78.57 |
| *Alphaproteobacteria* | Bacteria | *Caulobacter crescentus* | CCRE-XXX-01 | 3737 | 31.95 | 14.29 | 7.14 | 0.00 | 0.00 | 28.63 | 0.05 | 17.93 | 670 | 658 | 1.79 | 49 | 18 | 63.27 |
| *Alphaproteobacteria* | Bacteria | *Rhodopseudomonas palustris* | RPAL-009-01 | 4814 | 33.82 | 12.07 | 12.09 | 0.00 | 0.12 | 32.57 | 0.02 | 9.31 | 448 | 433 | 3.35 | 113 | 21 | 81.42 |
| *Alphaproteobacteria* | Bacteria | *Rickettsia conorii* | RCON-MAL-01 | 1374 | 25.55 | 15.65 | 5.68 | 0.00 | 0.00 | 27.07 | 0.07 | 25.98 | 357 | 350 | 1.96 | 30 | 18 | 40.00 |
| *Alphaproteobacteria* | Bacteria | *Rickettsia prowazekii* | RPRO-MAD-01 | 834 | 41.01 | 22.30 | 6.47 | 0.00 | 0.00 | 28.66 | 0.12 | 1.44 | 12 | 12 | 0.00 | 28 | 18 | 35.71 |
| *Alphaproteobacteria* | Bacteria | *Rickettsia typhi* | RTYP-144-01 | 838 | 39.26 | 22.79 | 6.56 | 0.00 | 0.00 | 28.52 | 0.00 | 2.86 | 24 | 23 | 4.17 | 29 | 18 | 37.93 |
| *Alphaproteobacteria* | Bacteria | *Sinorhizobium meliloti* | SMEL-102-01 | 6206 | 35.19 | 11.02 | 12.12 | 0.00 | 0.06 | 32.05 | 0.08 | 9.47 | 588 | 557 | 5.27 | 208 | 23 | 88.94 |
| *Alphaproteobacteria* | Bacteria | *Wolbachia pipientis* | WPIP-WME-01 | 1176 | 30.70 | 13.10 | 9.18 | 0.00 | 0.09 | 16.50 | 0.94 | 29.51 | 347 | 287 | 17.29 | 30 | 19 | 36.67 |
| *Betaproteobacteria* | Bacteria | *Bordetella bronchiseptica* | BBRO-252-01 | 4994 | 37.18 | 15.04 | 11.43 | 0.02 | 0.00 | 33.56 | 0.00 | 2.76 | 138 | 132 | 4.35 | 141 | 21 | 85.11 |
| *Betaproteobacteria* | Bacteria | *Bordetella parapertussis* | BPAR-253-01 | 4185 | 37.90 | 15.77 | 12.31 | 0.00 | 0.02 | 33.45 | 0.00 | 0.55 | 23 | 23 | 0.00 | 124 | 20 | 83.87 |
| *Betaproteobacteria* | Bacteria | *Bordetella pertussis* | BPER-251-01 | 3447 | 42.15 | 14.88 | 11.46 | 0.00 | 0.00 | 31.13 | 0.00 | 0.38 | 13 | 13 | 0.00 | 85 | 20 | 76.47 |
| *Betaproteobacteria* | Bacteria | *Burkholderia mallei* | BMAL-344-01 | 4764 | 33.69 | 12.13 | 10.10 | 0.00 | 0.00 | 33.19 | 0.06 | 10.83 | 516 | 515 | 0.19 | 75 | 18 | 76.00 |
| *Betaproteobacteria* | Bacteria | *Burkholderia pseudomallei* | BPSE-243-01 | 5729 | 32.71 | 12.67 | 9.76 | 0.00 | 0.05 | 36.73 | 0.07 | 8.01 | 459 | 451 | 1.74 | 92 | 18 | 80.43 |
| *Betaproteobacteria* | Bacteria | *Chromobacterium violaceum* | CVIO-472-01 | 4407 | 31.25 | 13.84 | 9.03 | 0.02 | 0.07 | 29.93 | 0.02 | 15.84 | 698 | 680 | 2.58 | 69 | 19 | 72.46 |
| *Betaproteobacteria* | Bacteria | *Neisseria meningitidis* | NMEN-Z24-01 | 2065 | 31.19 | 13.08 | 7.75 | 0.00 | 0.00 | 38.89 | 0.00 | 9.10 | 188 | 185 | 1.60 | 39 | 20 | 48.72 |
| *Betaproteobacteria* | Bacteria | *Neisseria meningitidis* | NMEN-MC5-01 | 2081 | 31.72 | 12.73 | 7.11 | 0.00 | 0.05 | 37.63 | 0.00 | 10.76 | 223 | 212 | 4.93 | 40 | 19 | 52.50 |
| *Betaproteobacteria* | Bacteria | *Nitrosomonas europaea* | NEUR-718-01 | 2461 | 32.18 | 16.17 | 10.32 | 0.00 | 0.12 | 31.57 | 0.08 | 9.55 | 235 | 231 | 1.70 | 51 | 21 | 58.82 |
| *Betaproteobacteria* | Bacteria | *Ralstonia solanacearum* | RSOL-XXX-01 | 5116 | 30.12 | 13.02 | 9.79 | 0.00 | 0.08 | 32.41 | 0.06 | 14.52 | 743 | 695 | 6.46 | 79 | 19 | 75.95 |
| *Gammaproteobacteria* | Bacteria | *Blochmannia floridanus* | BFLO-XXX-01 | 583 | 50.60 | 23.67 | 5.15 | 0.00 | 0.00 | 20.24 | 0.00 | 0.34 | 2 | 2 | 0.00 | 22 | 19 | 13.64 |
| *Gammaproteobacteria* | Bacteria | *Buchnera aphidicola* | BAPH-XBP-01 | 504 | 50.00 | 22.82 | 5.95 | 0.00 | 0.00 | 20.24 | 0.00 | 0.99 | 5 | 5 | 0.00 | 20 | 17 | 15.00 |
| *Gammaproteobacteria* | Bacteria | *Buchnera aphidicola* | BAPH-XSG-01 | 545 | 49.72 | 23.49 | 6.06 | 0.00 | 0.00 | 20.00 | 0.00 | 0.73 | 4 | 4 | 0.00 | 22 | 19 | 13.64 |
| *Gammaproteobacteria* | Bacteria | *Buchnera sp.* | BUCH-APS-01 | 575 | 49.91 | 22.09 | 5.74 | 0.00 | 0.00 | 21.91 | 0.00 | 0.35 | 2 | 2 | 0.00 | 22 | 18 | 18.18 |
| *Gammaproteobacteria* | Bacteria | *Coxiella burnetii* | CBUR-RSA-01 | 2045 | 29.83 | 12.22 | 6.60 | 0.00 | 0.15 | 18.44 | 0.49 | 32.27 | 660 | 635 | 3.79 | 36 | 18 | 50.00 |
| *Gammaproteobacteria* | Bacteria | *Erwinia carotovora subsp. atroseptica* | ECAR-043-01 | 4472 | 34.21 | 13.60 | 9.39 | 0.00 | 0.04 | 34.28 | 0.02 | 8.45 | 378 | 356 | 5.82 | 121 | 20 | 83.47 |
| *Gammaproteobacteria* | Bacteria | *Escherichia coli* | ECOL-CFT-01 | 5379 | 28.26 | 12.33 | 9.05 | 0.00 | 0.02 | 37.07 | 0.02 | 13.26 | 709 | 672 | 5.22 | 91 | 19 | 79.12 |
| *Gammaproteobacteria* | Bacteria | *Escherichia coli* | ECOL-MG1-01 | 4290 | 34.66 | 14.22 | 10.47 | 0.00 | 0.00 | 38.97 | 0.00 | 1.68 | 68 | 66 | 2.94 | 85 | 19 | 77.65 |
| *Gammaproteobacteria* | Bacteria | *Escherichia coli 0157:H7* | ECOL-RIM-01 | 5447 | 27.68 | 13.42 | 8.54 | 0.00 | 0.00 | 46.59 | 0.00 | 3.76 | 200 | 171 | 14.50 | 89 | 19 | 78.65 |
| *Gammaproteobacteria* | Bacteria | *Escherichia coli O157:H7* | ECOL-EDL-01 | 5349 | 28.10 | 13.42 | 8.66 | 0.00 | 0.00 | 47.19 | 0.00 | 2.64 | 131 | 114 | 12.98 | 88 | 19 | 78.41 |
| *Gammaproteobacteria* | Bacteria | *Haemophilus influenzae* | HINF-KW2-01 | 1707 | 38.25 | 15.70 | 9.14 | 0.00 | 0.00 | 32.51 | 0.00 | 4.39 | 75 | 75 | 0.00 | 50 | 17 | 66.00 |
| *Gammaproteobacteria* | Bacteria | *Legionella pneumophila* | LPNE-LEN-01 | 2934 | 32.07 | 15.75 | 6.51 | 0.00 | 0.00 | 40.01 | 0.07 | 5.59 | 164 | 162 | 1.22 | 47 | 20 | 57.45 |
| *Gammaproteobacteria* | Bacteria | *Legionella pneumophila* | LPNE-PHI-01 | 2942 | 32.43 | 15.94 | 6.22 | 0.00 | 0.03 | 40.62 | 0.00 | 4.76 | 140 | 134 | 4.29 | 49 | 19 | 61.22 |
| *Gammaproteobacteria* | Bacteria | *Mannheimia succiniciproducens* | MSUC-55E-01 | 2384 | 33.85 | 13.97 | 10.49 | 0.00 | 0.00 | 27.14 | 0.00 | 14.56 | 347 | 338 | 2.59 | 57 | 19 | 66.67 |
| *Gammaproteobacteria* | Bacteria | *Methylococcus capsulatus (Bath)* | MCAP-BAT-01 | 2959 | 32.14 | 13.75 | 12.10 | 0.00 | 0.24 | 27.10 | 0.14 | 14.53 | 430 | 420 | 2.33 | 51 | 18 | 64.71 |
| *Gammaproteobacteria* | Bacteria | *Pasteurella multocida* | PMUL-PM7-01 | 2014 | 37.93 | 15.09 | 11.17 | 0.00 | 0.00 | 31.03 | 0.00 | 4.77 | 96 | 94 | 2.08 | 67 | 19 | 71.64 |
| *Gammaproteobacteria* | Bacteria | *Photorhabdus luminescens* | PLUM-TO1-01 | 4683 | 27.72 | 11.36 | 7.99 | 0.04 | 0.02 | 38.24 | 0.15 | 14.48 | 678 | 566 | 16.52 | 82 | 20 | 75.61 |
| *Gammaproteobacteria* | Bacteria | *Pseudomonas aeruginosa* | PAER-PAO-01 | 5570 | 34.38 | 14.42 | 9.66 | 0.00 | 0.05 | 34.15 | 0.04 | 7.31 | 407 | 402 | 1.23 | 78 | 20 | 74.36 |
| *Gammaproteobacteria* | Bacteria | *Pseudomonas putida* | PPUT-KT2-01 | 5387 | 34.21 | 13.44 | 9.58 | 0.00 | 0.04 | 33.67 | 0.02 | 9.04 | 487 | 465 | 4.52 | 90 | 20 | 77.78 |
| *Gammaproteobacteria* | Bacteria | *Pseudomonas syringae pv. tomato* | PSYR-DC3-01 | 5471 | 31.77 | 12.76 | 11.77 | 0.00 | 0.05 | 32.54 | 0.07 | 11.04 | 604 | 573 | 5.13 | 112 | 20 | 82.14 |
| *Gammaproteobacteria* | Bacteria | *Salmonella enterica* | SENT-TY2-01 | 4323 | 32.15 | 12.47 | 9.90 | 0.00 | 0.00 | 45.04 | 0.00 | 0.44 | 16 | 14 | 12.50 | 71 | 20 | 71.83 |
| *Gammaproteobacteria* | Bacteria | *Salmonella enterica serovar Typhi* | SENT-CT1-02 | 4767 | 29.62 | 11.62 | 9.57 | 0.00 | 0.02 | 44.77 | 0.00 | 4.41 | 205 | 199 | 2.93 | 71 | 20 | 71.83 |
| *Gammaproteobacteria* | Bacteria | *Salmonella enterica serovar Typhimurium* | SENT-LT2-01 | 4553 | 32.66 | 13.13 | 9.84 | 0.00 | 0.02 | 41.60 | 0.00 | 2.75 | 122 | 118 | 3.28 | 72 | 20 | 72.22 |
| *Gammaproteobacteria* | Bacteria | *Shewanella oneidensis* | SONE-MR1-01 | 4867 | 25.66 | 14.55 | 9.80 | 0.04 | 0.14 | 27.33 | 0.12 | 22.35 | 1088 | 1028 | 5.51 | 58 | 17 | 70.69 |
| *Gammaproteobacteria* | Bacteria | *Shigella flexneri* | SFLE-457-01 | 4068 | 31.24 | 15.66 | 12.44 | 0.00 | 0.00 | 40.00 | 0.00 | 0.66 | 24 | 22 | 8.33 | 77 | 19 | 75.32 |
| *Gammaproteobacteria* | Bacteria | *Shigella flexneri (serotype 2a)* | SFLE-301-01 | 4452 | 29.04 | 16.11 | 11.93 | 0.00 | 0.00 | 41.42 | 0.00 | 1.50 | 63 | 56 | 11.11 | 77 | 19 | 75.32 |
| *Gammaproteobacteria* | Bacteria | *Vibrio cholerae* | VCHO-N16-01 | 3835 | 31.60 | 14.86 | 8.21 | 0.00 | 0.00 | 32.18 | 0.00 | 13.14 | 504 | 463 | 8.13 | 79 | 19 | 75.95 |
| *Gammaproteobacteria* | Bacteria | *Vibrio parahaemolyticus* | VPAR-RIM-01 | 4832 | 30.86 | 14.09 | 8.84 | 0.00 | 0.04 | 32.10 | 0.02 | 14.05 | 678 | 646 | 4.72 | 92 | 19 | 79.35 |
| *Gammaproteobacteria* | Bacteria | *Vibrio vulnificus* | VVUL-YJ0-01 | 5028 | 29.53 | 13.68 | 8.17 | 0.00 | 0.04 | 32.84 | 0.08 | 15.65 | 786 | 705 | 10.31 | 91 | 20 | 78.02 |
| *Gammaproteobacteria* | Bacteria | *Wigglesworthia glossinidia brevipalpis* | WGLO-BRE-01 | 611 | 44.84 | 25.86 | 4.58 | 0.00 | 0.00 | 22.26 | 0.00 | 2.45 | 15 | 15 | 0.00 | 19 | 15 | 21.05 |
| *Gammaproteobacteria* | Bacteria | *Xanthomonas axonopodis pv. citri* | XAXO-306-02 | 4427 | 28.60 | 16.67 | 6.69 | 0.00 | 0.00 | 40.32 | 0.05 | 7.68 | 340 | 332 | 2.35 | 42 | 18 | 57.14 |
| *Gammaproteobacteria* | Bacteria | *Xanthomonas campestris pv. campestris* | XCAM-AT3-01 | 4181 | 29.85 | 16.72 | 7.32 | 0.00 | 0.02 | 41.40 | 0.02 | 4.66 | 195 | 181 | 7.18 | 41 | 17 | 58.54 |
| *Gammaproteobacteria* | Bacteria | *Xylella fastidiosa* | XFAS-9A5-01 | 2830 | 23.78 | 11.66 | 4.81 | 0.00 | 0.00 | 37.70 | 0.00 | 22.05 | 624 | 599 | 4.01 | 37 | 19 | 48.65 |
| *Gammaproteobacteria* | Bacteria | *Xylella fastidiosa* | XFAS-XPD-01 | 2036 | 31.39 | 15.67 | 6.24 | 0.00 | 0.00 | 44.74 | 0.00 | 1.96 | 40 | 31 | 22.50 | 36 | 19 | 47.22 |
| *Gammaproteobacteria* | Bacteria | *Yersinia pestis* | YPES-CO9-01 | 4093 | 30.30 | 13.41 | 12.53 | 0.00 | 0.00 | 41.46 | 0.00 | 2.30 | 93 | 93 | 0.00 | 103 | 19 | 81.55 |
| *Gammaproteobacteria* | Bacteria | *Yersinia pestis* | YPES-KIM-01 | 4281 | 29.29 | 12.80 | 11.38 | 0.00 | 0.00 | 39.15 | 0.00 | 7.38 | 312 | 311 | 0.32 | 107 | 19 | 82.24 |
| *Gammaproteobacteria* | Bacteria | *Yersinia pseudotuberculosis* | YPSE-953-01 | 4038 | 32.32 | 14.07 | 8.92 | 0.00 | 0.00 | 41.85 | 0.02 | 2.82 | 113 | 112 | 0.89 | 111 | 19 | 82.88 |
| *Deltaproteobacteria* | Bacteria | *Bdellovibrio bacteriovorus* | BBAC-100-01 | 3583 | 27.35 | 11.36 | 7.56 | 0.06 | 0.08 | 18.84 | 0.45 | 34.30 | 1228 | 1203 | 2.04 | 66 | 21 | 68.18 |
| *Deltaproteobacteria* | Bacteria | *Desulfotalea psychrophila* | DPSY-V54-01 | 3236 | 32.20 | 10.26 | 14.62 | 0.03 | 0.37 | 21.45 | 0.12 | 20.95 | 678 | 654 | 3.54 | 68 | 20 | 70.59 |
| *Deltaproteobacteria* | Bacteria | *Desulfovibrio vulgaris* | DVUL-HIL-01 | 3514 | 29.23 | 8.76 | 13.92 | 0.00 | 0.26 | 19.55 | 0.00 | 28.29 | 994 | 961 | 3.32 | 59 | 19 | 67.80 |
| *Deltaproteobacteria* | Bacteria | *Geobacter sulfurreducens* | GSUL-PCA-01 | 3445 | 35.88 | 9.70 | 14.95 | 0.00 | 0.35 | 19.54 | 0.09 | 19.51 | 671 | 637 | 5.07 | 60 | 18 | 70.00 |
| *Epsilonproteobacteria* | Bacteria | *Campylobacter jejuni* | CJEJ-NCT-01 | 1634 | 36.60 | 12.67 | 11.14 | 0.00 | 0.06 | 27.60 | 0.31 | 11.63 | 189 | 177 | 6.35 | 42 | 18 | 57.14 |
| *Epsilonproteobacteria* | Bacteria | *Helicobacter hepaticus* | HHEP-449-01 | 1875 | 29.65 | 11.68 | 9.76 | 0.00 | 0.16 | 27.73 | 0.21 | 20.80 | 390 | 372 | 4.62 | 31 | 19 | 38.71 |
| *Epsilonproteobacteria* | Bacteria | *Helicobacter pylori* | HPYL-J99-01 | 1491 | 30.65 | 13.35 | 9.52 | 0.00 | 0.00 | 43.39 | 0.07 | 3.02 | 44 | 44 | 0.00 | 32 | 20 | 37.50 |
| *Epsilonproteobacteria* | Bacteria | *Helicobacter pylori* | HPYL-266-01 | 1575 | 29.27 | 12.63 | 9.33 | 0.00 | 0.00 | 42.98 | 0.13 | 5.65 | 87 | 85 | 2.30 | 32 | 21 | 34.38 |
| *Epsilonproteobacteria* | Bacteria | *Wolinella succinogenes strain DSM 1740* | WSUC-740-01 | 2044 | 36.84 | 12.28 | 13.99 | 0.00 | 0.15 | 27.15 | 0.05 | 9.54 | 195 | 195 | 0.00 | 49 | 19 | 61.22 |
| *Cyanobacteria* | Bacteria | *Anabaena sp.* | NOST-PCC-01 | 6129 | 29.17 | 11.81 | 9.72 | 0.03 | 0.21 | 25.91 | 0.16 | 22.97 | 1407 | 1296 | 7.89 | 107 | 22 | 79.44 |
| *Cyanobacteria* | Bacteria | *Gloeobacter violaceus* | GVIO-421-01 | 4430 | 28.89 | 13.39 | 8.78 | 0.07 | 0.27 | 28.87 | 0.18 | 19.55 | 866 | 790 | 8.78 | 67 | 19 | 71.64 |
| *Cyanobacteria* | Bacteria | *Prochlorococcus marinus* | PMAR-MED-01 | 1712 | 36.45 | 17.93 | 7.54 | 0.00 | 0.00 | 29.15 | 0.06 | 8.88 | 152 | 147 | 3.29 | 44 | 21 | 52.27 |
| *Cyanobacteria* | Bacteria | *Prochlorococcus marinus* | PMAR-SS1-01 | 1882 | 32.68 | 17.00 | 7.28 | 0.00 | 0.16 | 29.49 | 0.11 | 13.28 | 301 | 247 | 17.94 | 49 | 17 | 65.31 |
| *Cyanobacteria* | Bacteria | *Prochlorococcus marinus* | PMAR-MIT-01 | 2265 | 30.64 | 17.00 | 7.37 | 0.00 | 0.09 | 31.57 | 0.04 | 13.29 | 250 | 233 | 6.80 | 41 | 20 | 51.22 |
| *Cyanobacteria* | Bacteria | *Synechococcus sp.* | SYCC-WH8-01 | 2517 | 28.96 | 16.41 | 7.19 | 0.00 | 0.12 | 33.02 | 0.04 | 14.26 | 359 | 338 | 5.85 | 44 | 18 | 59.09 |
| *Cyanobacteria* | Bacteria | *Synechocystis sp.* | SYNE-PCC-01 | 3167 | 34.13 | 15.28 | 11.59 | 0.00 | 0.16 | 29.14 | 0.13 | 9.57 | 303 | 295 | 2.64 | 64 | 18 | 71.88 |
| *Cyanobacteria* | Bacteria | *Thermosynechococcus elongatus* | TELO-BP1-01 | 2475 | 37.54 | 16.20 | 10.79 | 0.00 | 0.04 | 26.14 | 0.08 | 9.21 | 228 | 228 | 0.00 | 53 | 20 | 62.26 |
| *Actinobacteridae* | *Bacteria* | *Bifidobacterium longum* | BLON-NCC-01 | *1729* | *37.77* | *13.36* | *11.28* | *0.00* | *0.17* | *###* | *0.06* | *14.23* | *245* | *245* | *0.00* | *54* | *17* | *68.52* |
| *Actinobacteridae* | Bacteria | *Corynebacterium diphtheriae* | CDIP-129-01 | 2272 | 32.66 | 10.61 | 10.26 | 0.00 | 0.00 | 33.63 | 0.04 | 12.81 | 291 | 283 | 2.75 | 62 | 18 | 70.97 |
| *Actinobacteridae* | Bacteria | *Corynebacterium efficiens* | CEFF-YS3-01 | 2950 | 31.05 | 9.80 | 11.19 | 0.00 | 0.20 | 36.20 | 0.07 | 11.49 | 339 | 320 | 5.60 | 58 | 18 | 68.97 |
| *Actinobacteridae* | Bacteria | *Corynebacterium glutamicum* | CGLU-XXX-01 | 3040 | 32.30 | 9.87 | 9.51 | 0.00 | 0.03 | 34.87 | 0.03 | 13.39 | 406 | 385 | 5.17 | 74 | 19 | 74.32 |
| *Actinobacteridae* | Bacteria | *Leifsonia xyli subsp. xyli* | LXYL-B07-01 | 2030 | 31.77 | 10.64 | 8.62 | 0.00 | 0.05 | 29.70 | 0.20 | 19.01 | 383 | 370 | 3.39 | 43 | 18 | 58.14 |
| *Actinobacteridae* | Bacteria | *Mycobacterium bovis* | MBOV-AF2-01 | 3920 | 31.38 | 9.64 | 9.16 | 0.00 | 0.00 | 49.44 | 0.00 | 0.38 | 15 | 15 | 0.00 | 38 | 15 | 60.53 |
| *Actinobacteridae* | Bacteria | *Mycobacterium leprae* | MLEP-XTN-01 | 1605 | 37.20 | 12.59 | 7.17 | 0.06 | 0.00 | 35.70 | 0.00 | 7.29 | 117 | 117 | 0.00 | 26 | 16 | 38.46 |
| *Actinobacteridae* | Bacteria | *Mycobacterium tuberculosis* | MTUB-H37-01 | 3924 | 31.37 | 9.89 | 9.23 | 0.00 | 0.00 | 49.11 | 0.00 | 0.41 | 16 | 14 | 12.50 | 39 | 15 | 61.54 |
| *Actinobacteridae* | Bacteria | *Mycobacterium tuberculosis* | MTUB-CDC-01 | 4203 | 29.00 | 8.71 | 8.40 | 0.00 | 0.00 | 44.68 | 0.00 | 9.21 | 387 | 382 | 1.29 | 38 | 15 | 60.53 |
| *Actinobacteridae* | Bacteria | *Nocardia farcinica* | NFAR-152-01 | 5936 | 27.31 | 8.54 | 9.42 | 0.02 | 0.03 | 32.88 | 0.03 | 21.77 | 1292 | 1247 | 3.48 | 75 | 18 | 76.00 |
| *Actinobacteridae* | Bacteria | *Propionibacterium acnes* | PACN-202-01 | 2297 | 36.40 | 11.14 | 9.45 | 0.00 | 0.04 | 25.86 | 0.04 | 17.07 | 392 | 383 | 2.30 | 66 | 19 | 71.21 |
| *Actinobacteridae* | Bacteria | *Streptomyces avermitilis* | SAVE-XXX-01 | 7671 | 28.41 | 9.75 | 9.52 | 0.01 | 0.14 | 39.51 | 0.08 | 12.58 | 965 | 928 | 3.83 | 125 | 19 | 84.80 |
| *Actinobacteridae* | Bacteria | *Streptomyces coelicolor* | SCOE-A32-01 | 7810 | 27.73 | 8.90 | 10.79 | 0.00 | 0.05 | 40.01 | 0.04 | 12.47 | 974 | 931 | 4.41 | 114 | 19 | 83.33 |
| *Actinobacteridae* | Bacteria | *Symbiobacterium thermophilum* | SYTH-863-01 | 3337 | 34.07 | 7.49 | 15.19 | 0.00 | 0.27 | 22.00 | 0.06 | 20.92 | 698 | 657 | 5.87 | 95 | 18 | 81.05 |
| *Actinobacteridae* | Bacteria | *Tropheryma whipplei* | TWHI-TW0-01 | 783 | 42.15 | 14.05 | 9.07 | 0.00 | 0.00 | 31.80 | 0.00 | 2.94 | 23 | 23 | 0.00 | 31 | 15 | 51.61 |
| *Actinobacteridae* | Bacteria | *Tropheryma whipplei* | TWHI-TWI-01 | 808 | 41.09 | 13.49 | 8.91 | 0.00 | 0.00 | 31.19 | 0.00 | 5.32 | 43 | 43 | 0.00 | 31 | 15 | 51.61 |
| *Firmicutes* | Bacteria | *Bacillus anthracis* | BANT-AME-01 | 5311 | 30.94 | 7.53 | 11.69 | 0.00 | 0.02 | 43.01 | 0.02 | 6.80 | 361 | 345 | 4.43 | 125 | 22 | 82.40 |
| *Firmicutes* | Bacteria | *Bacillus cereus* | BCER-579-01 | 5255 | 32.54 | 7.94 | 11.72 | 0.00 | 0.00 | 42.09 | 0.13 | 5.58 | 293 | 281 | 4.10 | 122 | 22 | 81.97 |
| *Firmicutes* | Bacteria | *Bacillus cereus* | BCER-987-01 | 5603 | 30.86 | 7.39 | 11.76 | 0.00 | 0.02 | 39.69 | 0.02 | 10.26 | 575 | 558 | 2.96 | 131 | 21 | 83.97 |
| *Firmicutes* | Bacteria | *Bacillus halodurans* | BHAL-C12-01 | 4066 | 34.75 | 8.78 | 13.85 | 0.00 | 0.02 | 29.46 | 0.05 | 13.08 | 522 | 504 | 3.45 | 103 | 21 | 79.61 |
| *Firmicutes* | Bacteria | *Bacillus licheniformis* | BLIC-580-01 | 4161 | 35.88 | 9.44 | 11.42 | 0.02 | 0.07 | 35.78 | 0.00 | 7.38 | 307 | 296 | 3.58 | 100 | 20 | 80.00 |
| *Firmicutes* | Bacteria | *Bacillus subtilis* | BSUB-168-01 | 4093 | 35.96 | 9.36 | 11.26 | 0.00 | 0.10 | 34.67 | 0.00 | 8.65 | 354 | 344 | 2.82 | 92 | 20 | 78.26 |
| *Firmicutes* | Bacteria | *Clostridium acetobutylicum* | CACE-ATC-01 | 3916 | 32.61 | 9.68 | 13.76 | 0.03 | 0.36 | 25.72 | 0.33 | 17.52 | 686 | 660 | 3.79 | 106 | 20 | 81.13 |
| *Firmicutes* | Bacteria | *Clostridium perfringens* | CPER-X13-01 | 2723 | 35.03 | 11.31 | 13.00 | 0.04 | 0.22 | 26.26 | 0.37 | 13.77 | 375 | 371 | 1.07 | 80 | 21 | 73.75 |
| *Firmicutes* | Bacteria | *Clostridium tetani* | CTET-E88-01 | 2432 | 36.43 | 10.07 | 15.83 | 0.00 | 0.25 | 27.10 | 0.29 | 10.03 | 244 | 240 | 1.64 | 85 | 19 | 77.65 |
| *Firmicutes* | Bacteria | *Enterococcus faecalis* | EFAE-V58-01 | 3113 | 29.78 | 8.83 | 9.28 | 0.00 | 0.03 | 32.44 | 0.10 | 19.53 | 608 | 566 | 6.91 | 94 | 21 | 77.66 |
| *Firmicutes* | Bacteria | *Lactobacillus johnsonii* | LJOH-533-01 | 1821 | 34.43 | 11.75 | 8.79 | 0.05 | 0.16 | 31.63 | 0.00 | 13.18 | 240 | 234 | 2.50 | 88 | 25 | 71.59 |
| *Firmicutes* | Bacteria | *Lactobacillus plantarum* | LPLA-WCF-01 | 3009 | 34.76 | 9.47 | 10.40 | 0.00 | 0.07 | 30.08 | 0.10 | 15.12 | 455 | 428 | 5.93 | 87 | 19 | 78.16 |
| *Firmicutes* | Bacteria | *Lactococcus lactis* | LLAC-IL1-01 | 2266 | 35.35 | 11.39 | 9.66 | 0.00 | 0.04 | 28.24 | 0.13 | 15.18 | 344 | 314 | 8.72 | 69 | 20 | 71.01 |
| *Firmicutes* | Bacteria | *Listeria innocua* | LINN-CLI-01 | 2968 | 34.54 | 10.01 | 10.82 | 0.00 | 0.00 | 41.37 | 0.00 | 3.27 | 97 | 80 | 17.53 | 87 | 21 | 75.86 |
| *Firmicutes* | Bacteria | *Listeria monocytogenes* | LMON-365-01 | 2809 | 36.90 | 10.81 | 10.49 | 0.00 | 0.00 | 39.67 | 0.04 | 2.09 | 59 | 59 | 0.00 | 90 | 21 | 76.67 |
| *Firmicutes* | Bacteria | *Listeria monocytogenes* | LMON-854-01 | 2961 | 35.28 | 10.39 | 10.39 | 0.00 | 0.03 | 41.37 | 0.00 | 2.52 | 75 | 73 | 2.67 | 90 | 21 | 76.67 |
| *Firmicutes* | Bacteria | *Listeria monocytogenes* | LMON-858-01 | 3097 | 33.32 | 10.07 | 10.26 | 0.00 | 0.06 | 41.07 | 0.03 | 5.18 | 161 | 161 | 0.00 | 89 | 21 | 76.40 |
| *Firmicutes* | Bacteria | *Listeria monocytogenes* | LMON-EGD-01 | 2846 | 37.07 | 10.96 | 10.93 | 0.00 | 0.00 | 40.20 | 0.00 | 0.84 | 24 | 24 | 0.00 | 88 | 21 | 76.14 |
| *Firmicutes* | Bacteria | *Mycoplasma gallisepticum* | MGAL-RLO-01 | 726 | 33.20 | 14.05 | 5.79 | 0.00 | 0.00 | 28.93 | 1.24 | 16.80 | 122 | 87 | 28.69 | 34 | 16 | 52.94 |
| *Firmicutes* | Bacteria | *Mycoplasma genitalium* | MGEN-G37-01 | 479 | 39.46 | 16.08 | 4.59 | 0.00 | 0.00 | 39.46 | 0.00 | 0.42 | 2 | 2 | 0.00 | 27 | 17 | 37.04 |
| *Firmicutes* | Bacteria | *Mycoplasma hyopneumoniae* | MHYO-232-01 | 691 | 31.69 | 12.16 | 5.64 | 0.00 | 0.00 | 24.31 | 1.30 | 24.89 | 172 | 143 | 16.86 | 44 | 20 | 54.55 |
| *Firmicutes* | Bacteria | *Mycoplasma mobile* | MMOB-63K-01 | 635 | 37.95 | 16.38 | 5.67 | 0.00 | 0.16 | 24.88 | 0.79 | 14.17 | 90 | 81 | 10.00 | 26 | 16 | 38.46 |
| *Firmicutes* | Bacteria | *Mycoplasma mycoides subsp. mycoides SC strain PG1* | MMYC-G1T-01 | 1016 | 27.36 | 15.65 | 8.86 | 0.00 | 0.30 | 21.75 | 0.89 | 25.20 | 256 | 222 | 13.28 | 36 | 22 | 38.89 |
| *Firmicutes* | Bacteria | *Mycoplasma penetrans* | MPEN-HF2-01 | 1037 | 28.16 | 14.95 | 7.43 | 0.00 | 0.00 | 19.86 | 2.51 | 27.10 | 281 | 187 | 33.45 | 38 | 15 | 60.53 |
| *Firmicutes* | Bacteria | *Mycoplasma pneumoniae* | MPNE-M12-01 | 689 | 28.74 | 11.76 | 5.66 | 0.00 | 0.29 | 42.24 | 0.15 | 11.18 | 77 | 40 | 48.05 | 29 | 18 | 37.93 |
| *Firmicutes* | Bacteria | *Mycoplasma pulmonis* | MPUL-UAB-01 | 782 | 33.63 | 16.11 | 4.73 | 0.00 | 0.00 | 27.11 | 0.26 | 18.16 | 142 | 136 | 4.23 | 32 | 18 | 43.75 |
| *Firmicutes* | Bacteria | *Oceanobacillus iheyensis* | OIHE-HET-01 | 3496 | 37.41 | 8.72 | 12.59 | 0.00 | 0.11 | 31.15 | 0.03 | 9.98 | 349 | 346 | 0.86 | 103 | 21 | 79.61 |
| *Firmicutes* | Bacteria | *Phytoplasma asteris* | PAST-XOY-01 | 754 | 28.12 | 12.20 | 3.85 | 0.00 | 0.00 | 19.63 | 0.80 | 35.41 | 267 | 167 | 37.45 | 27 | 18 | 33.33 |
| *Firmicutes* | Bacteria | *Staphylococcus aureus* | SAUR-252-01 | 2656 | 35.09 | 10.47 | 10.92 | 0.00 | 0.00 | 40.78 | 0.04 | 2.71 | 71 | 70 | 1.41 | 73 | 20 | 72.60 |
| *Firmicutes* | Bacteria | *Staphylococcus aureus* | SAUR-476-01 | 2579 | 35.91 | 10.35 | 10.82 | 0.00 | 0.00 | 41.95 | 0.00 | 0.97 | 24 | 23 | 4.17 | 71 | 20 | 71.83 |
| *Firmicutes* | Bacteria | *Staphylococcus aureus MRSA* | SAUR-MW2-01 | 2658 | 35.13 | 10.12 | 10.91 | 0.00 | 0.00 | 43.02 | 0.04 | 0.79 | 21 | 21 | 0.00 | 73 | 20 | 72.60 |
| *Firmicutes* | Bacteria | *Staphylococcus aureus MRSA* | SAUR-N13-01 | 2623 | 35.90 | 10.25 | 11.89 | 0.00 | 0.00 | 41.77 | 0.04 | 0.15 | 4 | 4 | 0.00 | 72 | 20 | 72.22 |
| *Firmicutes* | Bacteria | *Staphylococcus aureus VRSA* | SAUR-MU5-01 | 2748 | 34.32 | 10.04 | 11.39 | 0.00 | 0.00 | 42.43 | 0.00 | 1.82 | 48 | 47 | 2.08 | 71 | 20 | 71.83 |
| *Firmicutes* | Bacteria | *Streptococcus agalactiae* | SAGA-260-01 | 2144 | 32.56 | 11.29 | 10.07 | 0.00 | 0.05 | 38.43 | 0.00 | 7.60 | 163 | 158 | 3.07 | 80 | 19 | 76.25 |
| *Firmicutes* | Bacteria | *Streptococcus agalactiae* | SAGA-NEM-01 | 2094 | 33.86 | 11.51 | 10.70 | 0.00 | 0.14 | 36.77 | 0.05 | 6.97 | 146 | 80 | 45.21 | 83 | 19 | 77.11 |
| *Firmicutes* | Bacteria | *Streptococcus mutans* | SMUT-UA1-01 | 1960 | 34.64 | 11.22 | 11.68 | 0.00 | 0.00 | 31.28 | 0.05 | 11.12 | 218 | 212 | 2.75 | 90 | 21 | 76.67 |
| *Firmicutes* | Bacteria | *Streptococcus pneumoniae* | SPNE-TIG-01 | 2140 | 31.78 | 10.05 | 9.95 | 0.00 | 0.05 | 39.86 | 0.00 | 8.32 | 178 | 172 | 3.37 | 86 | 22 | 74.42 |
| *Firmicutes* | Bacteria | *Streptococcus pneumoniae* | SPNE-XR6-01 | 2043 | 33.87 | 10.67 | 10.13 | 0.00 | 0.00 | 42.14 | 0.00 | 3.18 | 65 | 65 | 0.00 | 89 | 22 | 75.28 |
| *Firmicutes* | Bacteria | *Streptococcus pyogenes* | SPYO-394-01 | 1886 | 30.65 | 11.29 | 9.07 | 0.00 | 0.00 | 45.86 | 0.00 | 3.13 | 59 | 55 | 6.78 | 60 | 17 | 71.67 |
| *Firmicutes* | Bacteria | *Streptococcus pyogenes M1* | SPYO-SF3-01 | 1696 | 33.90 | 11.38 | 9.96 | 0.00 | 0.00 | 43.16 | 0.06 | 1.53 | 26 | 26 | 0.00 | 61 | 17 | 72.13 |
| *Firmicutes* | Bacteria | *Streptococcus pyogenes M18* | SPYO-MGA-01 | 1845 | 31.38 | 11.11 | 8.83 | 0.00 | 0.05 | 46.45 | 0.00 | 2.17 | 40 | 38 | 5.00 | 61 | 18 | 70.49 |
| *Firmicutes* | Bacteria | *Streptococcus pyogenes M3* | SPYO-SSI-01 | 1861 | 30.74 | 10.85 | 9.03 | 0.00 | 0.00 | 48.15 | 0.00 | 1.24 | 23 | 22 | 4.35 | 60 | 17 | 71.67 |
| *Firmicutes* | Bacteria | *Streptococcus pyogenes M3* | SPYO-XM3-01 | 1865 | 30.99 | 11.21 | 9.12 | 0.00 | 0.00 | 47.94 | 0.00 | 0.75 | 14 | 14 | 0.00 | 61 | 17 | 72.13 |
| *Firmicutes* | Bacteria | *Thermoanaerobacter tengcongensis* | TTEN-MB4-01 | 2588 | 36.48 | 8.50 | 16.42 | 0.00 | 0.54 | 22.30 | 0.00 | 15.77 | 408 | 384 | 5.88 | 82 | 22 | 73.17 |
| *Firmicutes* | Bacteria | *Ureaplasma urealyticum* | UURE-SV3-01 | 613 | 29.69 | 18.92 | 7.18 | 0.00 | 0.00 | 23.16 | 1.14 | 19.90 | 122 | 116 | 4.92 | 25 | 17 | 32.00 |
| *Spirochaetes* | Bacteria | *Borrelia burgdorferi* | BBUR-B31-01 | 1639 | 18.06 | 8.60 | 5.98 | 0.18 | 0.18 | 14.09 | 1.10 | 51.80 | 849 | 467 | 44.99 | 27 | 18 | 33.33 |
| *Spirochaetes* | Bacteria | *Leptospira interrogans* | LINT-130-01 | 3660 | 26.61 | 10.33 | 9.10 | 0.00 | 0.03 | 50.46 | 0.03 | 3.44 | 126 | 125 | 0.79 | 47 | 21 | 55.32 |
| *Spirochaetes* | Bacteria | *Leptospira interrogans* | LINT-566-01 | 4727 | 21.01 | 8.23 | 7.70 | 0.00 | 0.00 | 42.35 | 0.02 | 20.69 | 978 | 915 | 6.44 | 46 | 21 | 54.35 |
| *Spirochaetes* | Bacteria | *Treponema denticola* | TDEN-405-01 | 2767 | 26.85 | 7.91 | 11.10 | 0.00 | 0.36 | 27.32 | 0.14 | 26.31 | 728 | 673 | 7.55 | 129 | 18 | 86.05 |
| *Spirochaetes* | Bacteria | *Treponema pallidum* | TPAL-NIC-01 | 1030 | 27.86 | 13.98 | 7.48 | 0.00 | 0.00 | 37.96 | 0.10 | 12.62 | 130 | 122 | 6.15 | 27 | 16 | 40.74 |
| *others* | Bacteria | *Aquifex aeolicus* | AAEO-VF5-01 | 1553 | 43.46 | 10.82 | 14.29 | 0.06 | 2.19 | 15.33 | 0.00 | 13.84 | 215 | 209 | 2.79 | 31 | 19 | 38.71 |
| *others* | Bacteria | *Bacteroides fragilis* | BFRA-H46-01 | 4625 | 24.35 | 10.12 | 9.34 | 0.02 | 0.15 | 33.25 | 0.06 | 22.70 | 1050 | 996 | 5.14 | 61 | 22 | 63.93 |
| *others* | Bacteria | *Bacteroides thetaiotaomicron* | BTHE-VPI-01 | 4778 | 25.37 | 13.23 | 10.03 | 0.04 | 0.10 | 36.56 | 0.21 | 14.46 | 691 | 644 | 6.80 | 56 | 22 | 60.71 |
| *others* | Bacteria | *Chlamydia pneumoniae* | CPNE-AR3-01 | 1119 | 28.60 | 14.48 | 5.36 | 0.00 | 0.00 | 44.77 | 0.00 | 6.79 | 76 | 76 | 0.00 | 24 | 16 | 33.33 |
| *others* | Bacteria | *Chlamydia pneumoniae* | CPNE-CWL-01 | 1052 | 30.51 | 15.30 | 5.70 | 0.00 | 0.00 | 48.38 | 0.00 | 0.10 | 1 | 1 | 0.00 | 24 | 16 | 33.33 |
| *others* | Bacteria | *Chlamydia pneumoniae* | CPNE-J13-01 | 1070 | 30.00 | 15.61 | 5.61 | 0.00 | 0.00 | 48.41 | 0.00 | 0.37 | 4 | 4 | 0.00 | 24 | 16 | 33.33 |
| *others* | Bacteria | *Chlamydia trachomatis* | CTRA-MOP-01 | 921 | 33.66 | 15.53 | 6.08 | 0.00 | 0.00 | 39.85 | 0.00 | 4.89 | 45 | 45 | 0.00 | 23 | 17 | 26.09 |
| *others* | Bacteria | *Chlamydia trachomatis* | CTRA-SVD-01 | 894 | 34.34 | 16.11 | 5.26 | 0.00 | 0.00 | 41.39 | 0.00 | 2.91 | 26 | 26 | 0.00 | 24 | 17 | 29.17 |
| *others* | Bacteria | *Chlamydophila caviae* | CCAV-GPI-01 | 1005 | 32.34 | 15.22 | 6.07 | 0.00 | 0.00 | 38.81 | 0.10 | 7.46 | 75 | 72 | 4.00 | 26 | 17 | 34.62 |
| *others* | Bacteria | *Chlorobium tepidum* | CTEP-TLS-01 | 2252 | 34.55 | 9.59 | 12.74 | 0.00 | 0.44 | 15.72 | 0.13 | 26.82 | 604 | 595 | 1.49 | 45 | 19 | 57.78 |
| *others* | Bacteria | *Deinococcus radiodurans* | DRAD-XR1-01 | 3116 | 31.16 | 9.21 | 12.55 | 0.00 | 0.26 | 22.43 | 0.06 | 24.33 | 758 | 746 | 1.58 | 61 | 21 | 65.57 |
| *others* | Bacteria | *Fusobacterium nucleatum* | FNUC-ATC-01 | 2067 | 34.01 | 10.84 | 13.21 | 0.00 | 0.00 | 22.50 | 0.29 | 19.16 | 396 | 363 | 8.33 | 58 | 18 | 68.97 |
| *others* | Bacteria | *Parachlamydia sp.* | PCHL-E25-01 | 2031 | 28.02 | 13.93 | 6.35 | 0.00 | 0.10 | 19.10 | 0.98 | 31.51 | 640 | 590 | 7.81 | 40 | 19 | 52.50 |
| *others* | Bacteria | *Pirellula sp.* | PIRE-ST1-01 | 7325 | 19.34 | 6.89 | 5.91 | 0.07 | 0.33 | 14.12 | 0.41 | 52.93 | 3877 | 3585 | 7.53 | 61 | 20 | 67.21 |
| *others* | Bacteria | *Porphyromonas gingivalis* | PGIN-W83-01 | 1909 | 29.33 | 12.26 | 10.95 | 0.00 | 0.26 | 27.50 | 0.10 | 19.59 | 373 | 336 | 9.92 | 37 | 18 | 51.35 |
| *others* | Bacteria | *Thermotoga maritima* | TMAR-MSB-01 | 1849 | 42.89 | 9.09 | 17.79 | 0.00 | 1.24 | 13.63 | 0.11 | 15.25 | 282 | 272 | 3.55 | 78 | 21 | 73.08 |
| *others* | Bacteria | *Thermus thermophilus* | TTHE-B27-01 | 2210 | 36.56 | 8.10 | 15.88 | 0.05 | 0.54 | 20.63 | 0.05 | 18.19 | 402 | 386 | 3.98 | 45 | 19 | 57.78 |
|  |  |  |  |  |  |  |  |  |  |  |  |  |  |  |  |  |  |  |
| *Plantae (Spermatophyta)* | Eukaryota | *Arabidopsis thaliana* | ATHA-XXX-01 | 25761 | 19.93 | 12.38 | 0.64 | 1.39 | 0.12 | 3.08 | 23.48 | 38.99 | 10037 | 5043 | 49.76 | 132 | 27 | 79.55 |
| *Plantae (Rhodophyta)* | Eukaryota | *Cyanidioschyzon merolae* | CMER-10D-01 | 4772 | 26.89 | 15.38 | 0.65 | 3.54 | 0.08 | 3.31 | 26.13 | 24.02 | 1145 | 1118 | 2.36 | 55 | 24 | 56.36 |
| *Protist* | Eukaryota | *Cryptosporidium parvum* | CPAR-TII-01 | 3396 | 14.87 | 8.33 | 0.03 | 4.27 | 0.12 | 0.85 | 35.92 | 35.60 | 1209 | 1194 | 1.24 | 16 | 13 | 18.75 |
| *Protist* | Eukaryota | *Plasmodium falciparum* | PFAL-3D7-01 | 5295 | 16.73 | 11.73 | 0.23 | 3.17 | 0.19 | 1.91 | 25.87 | 40.17 | 2126 | 1775 | 16.51 | 28 | 17 | 39.29 |
| *Microsporidia* | Eukaryota | *Encephalitozoon cuniculi* | ECUN-XXX-01 | 1996 | 19.19 | 5.01 | 0.00 | 6.56 | 0.10 | 0.50 | 26.15 | 42.48 | 848 | 677 | 20.17 | 24 | 17 | 29.17 |
| *Fungi* | Eukaryota | *Ashbya gossypii* | AGOS-XXX-01 | 4726 | 23.87 | 11.60 | 0.02 | 3.24 | 0.00 | 0.04 | 57.34 | 3.89 | 183 | 182 | 0.55 | 43 | 25 | 41.86 |
| *Fungi* | Eukaryota | *Candida glabrata* | CGLA-138-01 | 5272 | 24.20 | 12.20 | 0.02 | 3.05 | 0.00 | 0.02 | 54.51 | 5.99 | 316 | 277 | 12.34 | 52 | 26 | 50.00 |
| *Fungi* | Eukaryota | *Debaryomyces hansenii CBS767* | DHAN-767-01 | 6896 | 23.68 | 12.02 | 0.06 | 2.70 | 0.09 | 0.46 | 41.98 | 19.01 | 1309 | 1124 | 14.13 | 59 | 26 | 55.93 |
| *Fungi* | Eukaryota | *Kluyveromyces lactis* | KLAC-210-01 | 5331 | 24.25 | 11.61 | 0.00 | 2.96 | 0.23 | 0.15 | 52.90 | 7.90 | 421 | 385 | 8.55 | 50 | 27 | 46.00 |
| *Fungi* | Eukaryota | *Neurospora crassa* | NCRA-XX3-01 | 10082 | 17.56 | 9.98 | 0.25 | 1.59 | 0.04 | 1.88 | 26.46 | 42.24 | 4191 | 4106 | 2.03 | 56 | 27 | 51.79 |
| *Fungi* | Eukaryota | *Saccharomyces cerevisiae* | SCER-S28-01 | 6357 | 22.64 | 10.96 | 0.02 | 3.21 | 0.00 | 0.03 | 49.14 | 14.00 | 888 | 782 | 11.94 | 59 | 28 | 52.54 |
| *Fungi* | Eukaryota | *Schizosaccharomyces pombe* | SPOM-XXX-01 | 4945 | 27.52 | 12.25 | 0.16 | 4.15 | 0.04 | 0.22 | 42.24 | 13.41 | 661 | 624 | 5.60 | 54 | 26 | 51.85 |
| *Fungi* | Eukaryota | *Yarrowia lipolytica* | YLIP-B99-01 | 6666 | 23.93 | 12.08 | 0.09 | 2.43 | 0.02 | 0.24 | 40.47 | 20.75 | 1381 | 1247 | 9.70 | 56 | 25 | 55.36 |
| *Metazoa (Arthropoda)* | Eukaryota | *Anopheles gambiae* | AGAM-PES-01 | 15101 | 16.69 | 12.69 | 0.77 | 1.40 | 0.01 | 1.65 | 51.65 | 15.14 | 2280 | 1704 | 25.26 | 86 | 25 | 70.93 |
| *Metazoa (Arthropoda)* | Eukaryota | *Drosophila melanogaster* | DMEL-XXX-02 | 18484 | 18.94 | 12.39 | 0.00 | 1.78 | 0.00 | 0.05 | 48.23 | 18.62 | 3436 | 2907 | 15.40 | 121 | 27 | 77.69 |
| *Metazoa (Nematoda)* | Eukaryota | *Caenorhabditis briggsae* | CBRI-XXX-01 | 19507 | 12.33 | 9.63 | 0.02 | 1.31 | 0.01 | 0.03 | 64.42 | 12.25 | 2386 | 1589 | 33.40 | 80 | 24 | 70.00 |
| *Metazoa (Nematoda)* | Eukaryota | *Caenorhabditis elegans* | CELE-XXX-01 | 19957 | 12.92 | 9.04 | 0.02 | 1.32 | 0.01 | 0.01 | 66.88 | 9.81 | 1950 | 1633 | 16.26 | 75 | 24 | 68.00 |
| *Metazoa (Deuterostoma)* | Eukaryota | *Homo sapiens* | HSAP-XXX-03 | 32035 | 15.36 | 12.08 | 0.00 | 1.77 | 0.00 | 0.09 | 59.98 | 10.71 | 3424 | 2372 | 30.72 | 150 | 28 | 81.33 |
| *Metazoa (Deuterostoma)* | Eukaryota | *Mus musculus* | MMUS-XXX-02 | 25371 | 14.99 | 11.98 | 0.01 | 2.35 | 0.00 | 0.02 | 62.90 | 7.75 | 1964 | 1564 | 20.37 | 93 | 24 | 74.19 |
