# Supplementary material for: The global landscape of sequence diversity
Source: Genome Biol. 2007 Nov 8;8(11):R238. doi: 10.1186/gb-2007-8-11-r238 (PMC2258180; doi:10.1186/gb-2007-8-11-r238)
Supplement: Additional data file 3 — Species used in the study together with a detailed breakdown of the taxonomic relationships of their sequences. [file gb-2007-8-11-r238-S3.doc]

**Table S2.** List of partial genomes analysed in the study obtained from the PartiGeneDB database. Species are ordered by major taxonomic groups: Protists; Plants; Fungi; Nematodes; Arthropods and Deuterostomes. For each species the number of sequences associated with the partial genome are given, together with the percentage of genes shared between the major taxonomic groupings (according to BLAST similarity scores – see methods). P = Protists; F=Fungi; M=Metazoa (incl. Nematodes, Arthropods and Deuterostomes); V= Plants (Viridiplantae). *Sequences* indicates the number of distinct sequences, created through clustering expressed sequence tags, associated with each partial genome. *GF* indicates the number of gene families associated with each partial genomes (see methods for details of how gene families were calculated). *SS (%)* and *SS (#)* indicate the percentage and number of species specific sequences i.e. sequences which did not share similarity with a sequence from any other partial genome. *GF of SS (#)* indicates the number of gene families associated with the species specific sequences as determined through our gene family analysis (see methods). *All Dup (%)* and *SS Dup (%)* indicates the frequency of putative duplication events in all and species specific sequences respectively. These were calculated by comparing the number of sequences and the number of gene families using the following: All Dup(%) = 100 x (1 – (*GF* / *Sequences*)); *SS Dup (%)* = 100 x (1 – (*GF of SS(#)* / *SS(# in GF)*)).

| *Protist Species* | *Taxa* | *Species Identifier* | *Sequences* | *GF* | *PFMV* | *PFM* | *PFV* | *PMV* | *FMV* | *PF* | *PM* | *PV* | *FM* | *FV* | *MV* | *P* | *F* | *M* | *V* | *SS (%)* | *SS (#)* | *GF of SS (#)* | *All Dup (%)* | *SS Dup (%)* |
| --- | --- | --- | --- | --- | --- | --- | --- | --- | --- | --- | --- | --- | --- | --- | --- | --- | --- | --- | --- | --- | --- | --- | --- | --- |
| *Alexandrium tamarense* | *Alveolata* | ATD | 3562 | 3075 | 9.9 | 0.2 | 0.6 | 1.4 | 1.7 | 0.1 | 0.5 | 1.9 | 0.3 | 0.4 | 1.2 | 7.3 | 0.5 | 1.5 | 2.4 | 69.8 | 2486 | 2318 | 13.6 | 6.8 |
| *Amphidinium carterae* | *Alveolata* | ACD | 2157 | 1924 | 12.0 | 0.4 | 0.9 | 1.6 | 1.9 | 0.2 | 1.0 | 2.3 | 0.6 | 0.6 | 1.8 | 8.9 | 0.4 | 2.4 | 2.8 | 62.3 | 1343 | 1283 | 10.8 | 4.5 |
| *Gregarina niphandrodes* | *Alveolata* | GNC | 588 | 445 | 21.4 | 0.7 | 0.5 | 1.2 | 1.2 | 0.3 | 0.0 | 0.3 | 0.3 | 0.3 | 1.4 | 1.5 | 1.0 | 0.9 | 1.2 | 67.7 | 398 | 326 | 24.3 | 18.1 |
| *Lingulodinium polyedrum* | *Alveolata* | LPC | 1030 | 896 | 7.4 | 0.4 | 0.1 | 0.8 | 0.4 | 0.2 | 0.4 | 1.8 | 0.5 | 0.3 | 0.6 | 7.7 | 0.4 | 1.4 | 1.3 | 76.5 | 787 | 735 | 12.9 | 6.6 |
| *Neospora hughesi* | *Alveolata* | NHC | 780 | 699 | 11.2 | 0.3 | 0.1 | 1.0 | 0.4 | 0.1 | 0.4 | 0.3 | 0.1 | 0.3 | 0.3 | 5.3 | 0.1 | 0.5 | 0.6 | 79.1 | 617 | 560 | 10.4 | 9.2 |
| *Plasmodium berghei* | *Alveolata* | PBC | 4740 | 3926 | 12.4 | 0.3 | 0.2 | 1.3 | 0.9 | 0.2 | 0.9 | 0.5 | 0.6 | 0.2 | 0.7 | 22.2 | 0.2 | 4.4 | 0.4 | 54.6 | 2589 | 2441 | 17.2 | 5.7 |
| *Plasmodium yoelii* | *Alveolata* | PYC | 7913 | 6402 | 7.9 | 0.3 | 0.2 | 1.4 | 1.0 | 0.2 | 0.6 | 0.4 | 0.3 | 0.1 | 0.7 | 13.0 | 0.1 | 0.8 | 0.7 | 72.3 | 5700 | 4920 | 18.9 | 13.7 |
| *Sarcocystis neurona* | *Alveolata* | SNC | 2371 | 1950 | 11.6 | 0.3 | 0.2 | 1.5 | 0.6 | 0.0 | 0.7 | 0.5 | 0.2 | 0.0 | 1.1 | 2.5 | 0.1 | 4.2 | 0.3 | 76.1 | 1804 | 1556 | 17.8 | 13.7 |
| *Tetrahymena thermophila* | *Alveolata* | TTF | 4596 | 2335 | 22.0 | 0.5 | 0.3 | 1.6 | 4.5 | 0.0 | 0.7 | 0.4 | 1.0 | 0.3 | 4.0 | 0.5 | 0.4 | 10.0 | 7.2 | 46.5 | 2137 | 1349 | 49.2 | 36.9 |
| *Theileria parva* | *Alveolata* | TPC | 1967 | 1741 | 23.4 | 0.5 | 0.2 | 2.7 | 3.3 | 0.2 | 1.1 | 0.9 | 0.7 | 0.4 | 1.7 | 6.0 | 0.3 | 1.9 | 1.4 | 55.5 | 1088 | 1020 | 11.4 | 6.3 |
| *Leishmania major* | *Euglenozoa* | LMC | 1271 | 1040 | 7.2 | 0.3 | 0.0 | 0.2 | 0.4 | 0.2 | 0.6 | 0.3 | 0.0 | 0.1 | 0.3 | 3.7 | 0.2 | 0.5 | 0.4 | 85.6 | 1087 | 931 | 18.1 | 14.4 |
| *Trypanosoma brucei rhodesiense* | *Euglenozoa* | TBD | 2824 | 2510 | 8.4 | 0.1 | 0.1 | 0.7 | 0.9 | 0.1 | 0.3 | 0.0 | 0.3 | 0.1 | 0.7 | 2.2 | 0.3 | 1.2 | 0.6 | 83.9 | 2368 | 2162 | 11.1 | 8.7 |
| *Trypanosoma carassii* | *Euglenozoa* | TCD | 1167 | 888 | 12.8 | 0.3 | 0.3 | 0.9 | 0.5 | 0.0 | 0.4 | 0.3 | 0.2 | 0.1 | 0.3 | 3.9 | 0.1 | 0.5 | 0.6 | 78.8 | 916 | 724 | 23.6 | 21.0 |
| *Emiliania huxleyi* | *Haptophyceae* | EHC | 1506 | 1423 | 10.0 | 0.4 | 0.3 | 1.0 | 2.2 | 0.1 | 0.7 | 0.8 | 0.5 | 0.1 | 1.6 | 1.7 | 0.7 | 1.7 | 2.5 | 75.9 | 1142 | 1089 | 5.4 | 4.6 |
| *Fragilariopsis cylindrus* | *Stramenopiles* | FCC | 909 | 871 | 8.6 | 0.6 | 0.6 | 0.4 | 0.7 | 0.2 | 0.3 | 2.2 | 0.4 | 0.2 | 0.6 | 6.6 | 0.2 | 0.8 | 2.1 | 75.6 | 685 | 668 | 4.0 | 2.5 |
| *Phaeodactylum tricornutum* | *Stramenopiles* | PTF | 4567 | 3907 | 12.7 | 0.4 | 1.0 | 1.8 | 3.4 | 0.3 | 1.1 | 2.1 | 0.6 | 1.2 | 2.5 | 3.4 | 0.8 | 1.7 | 4.3 | 62.9 | 2869 | 2578 | 14.4 | 10.1 |
| *Phytophthora infestans* | *Stramenopiles* | PIC | 1602 | 1468 | 24.5 | 0.7 | 0.9 | 1.4 | 5.1 | 0.3 | 0.7 | 0.6 | 0.7 | 1.1 | 2.7 | 1.3 | 1.4 | 2.4 | 3.8 | 52.6 | 842 | 796 | 8.4 | 5.5 |
|  |  |  |  |  |  |  |  |  |  |  |  |  |  |  |  |  |  |  |  |  |  |  |  |  |
| *Plant Species* | *Taxa* | *Species Identifier* | *Genes* | *GF* | *PFMV* | *PFM* | *PFV* | *PMV* | *FMV* | *PF* | *PM* | *PV* | *FM* | *FV* | *MV* | *P* | *F* | *M* | *V* | *SS (%)* | *SS (#)* | *GF of SS (#)* | *All Dup (%)* | *SS Dup (%)* |
| *Ceratodon purpureus* | *Bryophyta* | CPD | 856 | 783 | 20.0 | 0.0 | 0.4 | 2.0 | 2.8 | 0.0 | 0.0 | 1.6 | 0.0 | 1.4 | 2.8 | 0.2 | 0.1 | 0.5 | 19.9 | 48.4 | 414 | 410 | 8.5 | 1.0 |
| *Dunaliella salina* | *Chlorophyta* | DSD | 1434 | 1329 | 19.4 | 0.1 | 1.0 | 1.7 | 2.4 | 0.0 | 0.3 | 2.6 | 0.4 | 1.0 | 2.4 | 0.7 | 0.1 | 1.1 | 9.8 | 57.0 | 818 | 791 | 7.3 | 3.3 |
| *Closterium peracerosum-strigosum-littorale complex* | *Desmidiales* | CPF | 750 | 693 | 26.9 | 0.3 | 1.2 | 2.1 | 2.8 | 0.0 | 0.3 | 3.3 | 0.0 | 1.2 | 3.6 | 0.4 | 0.0 | 0.4 | 21.1 | 36.4 | 273 | 254 | 7.6 | 7.0 |
| *Marchantia polymorpha* | *Marchantiopsida* | MPD | 1043 | 987 | 17.8 | 0.0 | 0.4 | 1.9 | 2.4 | 0.0 | 0.0 | 2.0 | 0.0 | 1.4 | 3.6 | 0.0 | 0.4 | 0.4 | 16.0 | 53.6 | 557 | 547 | 5.2 | 1.8 |
| *Selaginella lepidophylla* | *Lycopodiophyta* | SLC | 882 | 829 | 20.0 | 0.0 | 1.1 | 2.5 | 5.3 | 0.0 | 0.1 | 3.5 | 0.2 | 2.6 | 5.2 | 0.2 | 0.1 | 0.5 | 34.8 | 23.8 | 210 | 205 | 6.0 | 2.4 |
| *Ceratopteris richardii* | *Moniliformopses* | CRC | 2978 | 2279 | 28.6 | 0.0 | 1.1 | 2.6 | 5.0 | 0.0 | 0.0 | 1.9 | 0.1 | 2.5 | 5.2 | 0.1 | 0.6 | 0.3 | 26.6 | 25.2 | 751 | 704 | 23.5 | 6.3 |
| *Acorus americanus* | *Spermatophyta* | AAF | 1971 | 1751 | 24.2 | 0.0 | 1.0 | 3.4 | 4.5 | 0.0 | 0.0 | 2.2 | 0.1 | 2.3 | 5.7 | 0.1 | 0.1 | 0.2 | 36.8 | 19.4 | 382 | 374 | 11.2 | 2.1 |
| *Aegilops speltoides* | *Spermatophyta* | ASE | 2710 | 2282 | 21.4 | 0.0 | 0.8 | 2.4 | 4.6 | 0.0 | 0.0 | 2.3 | 0.1 | 1.9 | 4.8 | 0.0 | 0.0 | 0.1 | 41.0 | 20.4 | 552 | 536 | 15.8 | 2.9 |
| *Allium cepa* | *Spermatophyta* | ACF | 11409 | 7610 | 17.8 | 0.0 | 1.1 | 2.9 | 5.3 | 0.0 | 0.1 | 2.6 | 0.1 | 2.7 | 6.9 | 0.0 | 0.2 | 0.3 | 36.8 | 23.1 | 2637 | 2425 | 33.3 | 8.0 |
| *Amborella trichopoda* | *Spermatophyta* | ATC | 4742 | 4162 | 15.5 | 0.1 | 0.9 | 2.3 | 4.7 | 0.0 | 0.0 | 2.1 | 0.0 | 2.0 | 5.0 | 0.0 | 0.0 | 0.2 | 33.9 | 33.3 | 1578 | 1511 | 12.2 | 4.2 |
| *Antirrhinum majus* | *Spermatophyta* | AMD | 2185 | 1698 | 23.9 | 0.0 | 1.4 | 2.7 | 5.3 | 0.0 | 0.0 | 3.1 | 0.0 | 2.9 | 5.5 | 0.0 | 0.0 | 0.0 | 41.7 | 13.3 | 291 | 276 | 22.3 | 5.2 |
| *Arachis hypogaea* | *Spermatophyta* | AHC | 1026 | 895 | 19.5 | 0.0 | 0.7 | 2.5 | 3.4 | 0.0 | 0.0 | 2.0 | 0.0 | 2.4 | 4.7 | 0.0 | 0.0 | 0.3 | 40.4 | 24.1 | 247 | 231 | 12.8 | 6.5 |
| *Avicennia marina* | *Spermatophyta* | AMC | 1311 | 1183 | 17.6 | 0.0 | 0.8 | 2.7 | 5.1 | 0.0 | 0.1 | 1.9 | 0.0 | 1.8 | 5.0 | 0.0 | 0.0 | 0.0 | 43.8 | 21.1 | 276 | 270 | 9.8 | 2.2 |
| *Betula pendula* | *Spermatophyta* | BPC | 1822 | 1477 | 22.2 | 0.1 | 1.3 | 0.9 | 3.5 | 0.1 | 0.0 | 1.0 | 1.1 | 2.4 | 4.1 | 0.0 | 10.6 | 0.1 | 21.8 | 30.8 | 560 | 516 | 18.9 | 7.9 |
| *Brassica rapa* | *Spermatophyta* | BRE | 4431 | 3437 | 19.9 | 0.0 | 0.7 | 2.4 | 3.5 | 0.0 | 0.0 | 1.7 | 0.0 | 2.1 | 5.0 | 0.0 | 0.0 | 0.1 | 38.8 | 25.7 | 1138 | 1088 | 22.4 | 4.4 |
| *Citrus reticulata* | *Spermatophyta* | CRD | 1897 | 1530 | 28.4 | 0.0 | 0.8 | 3.1 | 5.3 | 0.0 | 0.1 | 2.1 | 0.2 | 2.6 | 5.2 | 0.1 | 0.1 | 0.0 | 40.2 | 12.1 | 229 | 222 | 19.3 | 3.1 |
| *Citrus unshiu* | *Spermatophyta* | CUC | 1719 | 1569 | 15.6 | 0.1 | 0.4 | 2.2 | 3.5 | 0.0 | 0.0 | 1.5 | 0.0 | 1.5 | 3.4 | 0.0 | 0.1 | 0.2 | 49.5 | 22.2 | 381 | 375 | 8.7 | 1.6 |
| *Cryptomeria japonica* | *Spermatophyta* | CJC | 4389 | 3695 | 15.6 | 0.0 | 0.9 | 2.0 | 4.4 | 0.0 | 0.0 | 1.8 | 0.1 | 2.3 | 5.3 | 0.0 | 0.1 | 0.2 | 37.9 | 29.3 | 1283 | 1240 | 15.8 | 3.4 |
| *Cucumis sativus* | *Spermatophyta* | CSC | 1384 | 1242 | 21.0 | 0.0 | 0.4 | 2.3 | 4.0 | 0.0 | 0.1 | 2.5 | 0.1 | 2.5 | 4.3 | 0.0 | 0.0 | 0.1 | 37.0 | 25.7 | 347 | 339 | 9.7 | 2.3 |
| *Cycas rumphii* | *Spermatophyta* | CRE | 2963 | 2459 | 18.5 | 0.0 | 0.8 | 2.3 | 3.8 | 0.0 | 0.0 | 1.7 | 0.2 | 1.8 | 4.2 | 0.0 | 0.0 | 0.3 | 32.3 | 34.2 | 1010 | 913 | 16.9 | 9.6 |
| *Descurainia sophia* | *Spermatophyta* | DSC | 803 | 686 | 22.9 | 0.0 | 0.5 | 4.1 | 3.9 | 0.0 | 0.0 | 4.1 | 0.0 | 2.9 | 5.7 | 0.2 | 0.1 | 0.2 | 39.9 | 15.4 | 124 | 118 | 14.6 | 4.8 |
| *Eschscholzia californica* | *Spermatophyta* | ECC | 5431 | 4359 | 19.2 | 0.1 | 0.6 | 1.9 | 4.4 | 0.0 | 0.0 | 2.3 | 0.1 | 2.4 | 5.3 | 0.0 | 0.0 | 0.1 | 37.7 | 26.0 | 1413 | 1361 | 19.7 | 3.7 |
| *Eucalyptus grandis* | *Spermatophyta* | EGD | 1485 | 1335 | 18.7 | 0.1 | 0.9 | 2.6 | 4.7 | 0.0 | 0.0 | 2.1 | 0.0 | 3.0 | 5.7 | 0.0 | 0.1 | 0.1 | 45.9 | 16.3 | 242 | 241 | 10.1 | 0.4 |
| *Eucalyptus tereticornis* | *Spermatophyta* | ETC | 1093 | 969 | 25.3 | 0.0 | 1.2 | 4.1 | 6.6 | 0.0 | 0.0 | 1.9 | 0.2 | 3.7 | 5.3 | 0.0 | 0.0 | 0.1 | 47.1 | 4.6 | 50 | 50 | 11.3 | 0.0 |
| *Euphorbia esula* | *Spermatophyta* | EEC | 1181 | 1032 | 28.5 | 0.1 | 1.1 | 2.5 | 5.8 | 0.0 | 0.1 | 1.7 | 0.1 | 2.5 | 5.5 | 0.0 | 0.0 | 0.2 | 40.0 | 11.9 | 141 | 132 | 12.6 | 6.4 |
| *Ginkgo biloba* | *Spermatophyta* | GBC | 1355 | 1236 | 11.7 | 0.0 | 0.3 | 1.3 | 2.4 | 0.0 | 0.1 | 1.5 | 0.0 | 1.5 | 3.2 | 0.0 | 0.0 | 0.0 | 28.8 | 49.1 | 661 | 634 | 8.5 | 4.1 |
| *Gnetum gnemon* | *Spermatophyta* | GGC | 1597 | 1472 | 13.2 | 0.0 | 0.6 | 2.7 | 2.8 | 0.0 | 0.0 | 2.1 | 0.0 | 2.1 | 4.3 | 0.1 | 0.1 | 0.1 | 31.6 | 40.5 | 646 | 634 | 7.8 | 1.9 |
| *Gossypium hirsutum* | *Spermatophyta* | GHC | 8359 | 5858 | 20.9 | 0.0 | 1.0 | 2.5 | 4.9 | 0.0 | 0.0 | 1.9 | 0.1 | 2.3 | 5.4 | 0.0 | 1.0 | 0.2 | 36.9 | 22.8 | 1908 | 1764 | 29.9 | 7.5 |
| *Hedyotis centranthoides* | *Spermatophyta* | HCD | 4054 | 3478 | 15.6 | 0.0 | 0.7 | 2.0 | 3.3 | 0.0 | 0.0 | 1.8 | 0.0 | 2.0 | 3.7 | 0.0 | 0.1 | 0.3 | 40.0 | 30.5 | 1224 | 1189 | 14.0 | 2.9 |
| *Hedyotis terminalis* | *Spermatophyta* | HTC | 3695 | 3048 | 16.9 | 0.0 | 0.8 | 1.8 | 3.5 | 0.0 | 0.0 | 1.7 | 0.1 | 1.9 | 4.2 | 0.0 | 0.1 | 0.4 | 41.9 | 26.8 | 983 | 957 | 17.4 | 2.6 |
| *Helianthus argophyllus* | *Spermatophyta* | HAC | 5991 | 3384 | 12.2 | 0.0 | 0.7 | 2.0 | 3.0 | 0.0 | 0.0 | 1.7 | 0.0 | 3.0 | 3.8 | 0.0 | 0.1 | 0.3 | 41.1 | 32.2 | 1930 | 1436 | 43.5 | 25.6 |
| *Helianthus paradoxus* | *Spermatophyta* | HPD | 4954 | 3138 | 14.4 | 0.0 | 0.8 | 2.4 | 3.2 | 0.0 | 0.0 | 2.9 | 0.0 | 2.2 | 4.3 | 0.0 | 0.0 | 0.1 | 42.8 | 26.9 | 1335 | 1122 | 36.7 | 16.0 |
| *Ipomoea batatas* | *Spermatophyta* | IBC | 1640 | 1299 | 29.5 | 0.0 | 1.0 | 3.0 | 3.5 | 0.0 | 0.0 | 2.7 | 0.1 | 2.2 | 4.9 | 0.0 | 0.1 | 0.1 | 36.3 | 16.6 | 268 | 248 | 20.6 | 7.5 |
| *Ipomoea trifida* | *Spermatophyta* | ITC | 813 | 730 | 9.2 | 0.0 | 0.2 | 0.9 | 3.9 | 0.0 | 0.0 | 1.2 | 0.2 | 2.5 | 2.8 | 0.0 | 0.0 | 0.0 | 35.9 | 43.1 | 350 | 331 | 10.2 | 5.4 |
| *Linum usitatissimum* | *Spermatophyta* | LUC | 1100 | 888 | 18.2 | 0.0 | 0.8 | 2.2 | 4.5 | 0.0 | 0.0 | 2.8 | 0.0 | 2.2 | 4.6 | 0.0 | 0.1 | 0.2 | 30.4 | 34.1 | 371 | 325 | 19.0 | 12.4 |
| *Liriodendron tulipifera* | *Spermatophyta* | LTC | 3342 | 3007 | 14.9 | 0.1 | 0.6 | 1.8 | 4.2 | 0.0 | 0.0 | 1.8 | 0.0 | 1.6 | 4.6 | 0.0 | 0.1 | 0.1 | 36.0 | 34.1 | 1140 | 1088 | 10.0 | 4.6 |
| *Lupinus albus* | *Spermatophyta* | LAC | 1551 | 1289 | 26.0 | 0.0 | 1.1 | 3.5 | 4.8 | 0.0 | 0.0 | 1.6 | 0.0 | 3.1 | 5.2 | 0.0 | 0.1 | 0.1 | 41.1 | 13.4 | 208 | 204 | 16.9 | 1.9 |
| *Lycopersicon hirsutum* | *Spermatophyta* | LHC | 1708 | 1541 | 16.7 | 0.0 | 0.5 | 2.7 | 4.7 | 0.0 | 0.0 | 2.2 | 0.0 | 2.3 | 5.8 | 0.1 | 0.1 | 0.2 | 47.4 | 17.3 | 294 | 290 | 9.7 | 1.4 |
| *Lycopersicon pennellii* | *Spermatophyta* | LPD | 3319 | 2727 | 16.4 | 0.0 | 0.8 | 2.4 | 4.3 | 0.0 | 0.0 | 2.0 | 0.0 | 2.1 | 4.8 | 0.0 | 0.0 | 0.2 | 44.4 | 22.4 | 745 | 708 | 17.9 | 5.0 |
| *Nicotiana benthamiana* | *Spermatophyta* | NBD | 2767 | 1723 | 25.4 | 0.0 | 1.4 | 4.4 | 4.0 | 0.0 | 0.0 | 4.4 | 0.0 | 3.1 | 5.7 | 0.0 | 0.0 | 0.0 | 38.4 | 13.0 | 361 | 329 | 37.7 | 8.9 |
| *Nicotiana tabacum* | *Spermatophyta* | NTC | 9197 | 8570 | 5.1 | 0.0 | 0.3 | 0.8 | 1.7 | 0.0 | 0.0 | 0.8 | 0.1 | 1.1 | 2.1 | 0.0 | 0.0 | 0.1 | 24.3 | 63.6 | 5851 | 5694 | 6.8 | 2.7 |
| *Nuphar advena* | *Spermatophyta* | NAD | 5139 | 4271 | 15.2 | 0.0 | 0.8 | 1.8 | 4.0 | 0.0 | 0.1 | 1.7 | 0.1 | 1.6 | 4.4 | 0.1 | 0.0 | 0.6 | 32.6 | 37.0 | 1902 | 1727 | 16.9 | 9.2 |
| *Oryza minuta* | *Spermatophyta* | OMC | 3347 | 2865 | 16.3 | 0.0 | 1.1 | 2.7 | 5.5 | 0.0 | 0.0 | 3.4 | 0.0 | 2.2 | 4.8 | 0.0 | 0.0 | 0.2 | 41.0 | 22.6 | 756 | 727 | 14.4 | 3.8 |
| *Pennisetum glaucum* | *Spermatophyta* | PGC | 1927 | 1749 | 12.1 | 0.0 | 0.6 | 1.1 | 2.4 | 0.0 | 0.0 | 1.4 | 0.2 | 1.5 | 3.2 | 0.0 | 0.0 | 0.4 | 25.5 | 51.7 | 997 | 944 | 9.2 | 5.3 |
| *Persea americana* | *Spermatophyta* | PAE | 3327 | 2904 | 17.9 | 0.0 | 0.8 | 2.6 | 4.3 | 0.0 | 0.0 | 2.0 | 0.1 | 1.9 | 5.1 | 0.0 | 0.1 | 0.2 | 35.0 | 30.1 | 1001 | 976 | 12.7 | 2.5 |
| *Phaseolus coccineus* | *Spermatophyta* | PCE | 7185 | 5518 | 21.0 | 0.0 | 0.6 | 2.0 | 4.2 | 0.0 | 0.1 | 2.0 | 0.1 | 2.1 | 5.0 | 0.0 | 0.0 | 0.2 | 35.5 | 27.2 | 1952 | 1834 | 23.2 | 6.0 |
| *Picea glauca* | *Spermatophyta* | PGD | 5260 | 4086 | 16.4 | 0.0 | 0.8 | 1.9 | 4.0 | 0.0 | 0.0 | 1.3 | 0.0 | 2.7 | 5.3 | 0.0 | 0.0 | 0.1 | 44.2 | 23.1 | 1216 | 1154 | 22.3 | 5.1 |
| *Pinus pinaster* | *Spermatophyta* | PPF | 8379 | 5903 | 16.8 | 0.0 | 0.8 | 1.8 | 4.2 | 0.0 | 0.0 | 1.7 | 0.1 | 2.2 | 4.4 | 0.0 | 0.4 | 0.2 | 39.4 | 27.8 | 2323 | 2151 | 29.5 | 7.4 |
| *Pisum sativum* | *Spermatophyta* | PSC | 1204 | 953 | 19.3 | 0.0 | 1.0 | 2.7 | 4.5 | 0.0 | 0.1 | 3.3 | 0.0 | 3.2 | 5.8 | 0.0 | 0.1 | 0.1 | 43.4 | 16.4 | 198 | 167 | 20.8 | 15.7 |
| *Plumbago zeylanica* | *Spermatophyta* | PZC | 919 | 801 | 9.5 | 0.0 | 0.0 | 0.5 | 1.6 | 0.0 | 0.0 | 0.7 | 0.1 | 1.0 | 2.7 | 0.1 | 0.0 | 0.1 | 22.2 | 61.5 | 565 | 527 | 12.9 | 6.7 |
| *Poncirus trifoliata* | *Spermatophyta* | PTE | 2880 | 2421 | 19.9 | 0.0 | 1.1 | 2.9 | 5.1 | 0.0 | 0.1 | 2.7 | 0.0 | 2.8 | 5.8 | 0.0 | 0.0 | 0.1 | 44.1 | 15.3 | 440 | 433 | 15.9 | 1.6 |
| *Populus tremuloides* | *Spermatophyta* | PTG | 4985 | 3513 | 23.0 | 0.0 | 1.0 | 2.9 | 5.0 | 0.0 | 0.0 | 2.8 | 0.0 | 2.9 | 5.1 | 0.0 | 0.0 | 0.1 | 40.3 | 16.8 | 837 | 769 | 29.5 | 8.1 |
| *Prunus armeniaca* | *Spermatophyta* | PAD | 2995 | 2527 | 18.5 | 0.0 | 1.1 | 2.2 | 4.6 | 0.1 | 0.0 | 1.6 | 0.1 | 2.5 | 4.8 | 0.0 | 0.0 | 0.0 | 45.0 | 19.4 | 580 | 570 | 15.6 | 1.7 |
| *Prunus dulcis* | *Spermatophyta* | PDC | 1554 | 1312 | 16.9 | 0.0 | 0.8 | 2.6 | 3.7 | 0.0 | 0.0 | 1.8 | 0.0 | 3.2 | 5.3 | 0.1 | 0.0 | 0.2 | 48.6 | 16.7 | 260 | 250 | 15.6 | 3.8 |
| *Prunus persica* | *Spermatophyta* | PPE | 5312 | 3988 | 20.8 | 0.0 | 1.1 | 2.8 | 5.6 | 0.0 | 0.0 | 2.5 | 0.0 | 2.3 | 6.2 | 0.0 | 0.0 | 0.1 | 45.4 | 13.1 | 695 | 659 | 24.9 | 5.2 |
| *Ricinus communis* | *Spermatophyta* | RCC | 648 | 599 | 24.5 | 0.0 | 0.0 | 2.5 | 3.4 | 0.0 | 0.0 | 1.7 | 0.0 | 2.8 | 4.8 | 0.0 | 0.0 | 0.2 | 39.4 | 20.8 | 134 | 128 | 7.4 | 4.5 |
| *Robinia pseudoacacia* | *Spermatophyta* | RPC | 2430 | 2146 | 11.3 | 0.0 | 0.2 | 1.1 | 2.3 | 0.0 | 0.0 | 1.3 | 0.0 | 0.9 | 2.6 | 0.0 | 0.4 | 0.3 | 23.3 | 56.1 | 1364 | 1269 | 11.7 | 7.0 |
| *Rosa chinensis* | *Spermatophyta* | RCD | 884 | 751 | 23.0 | 0.0 | 1.0 | 2.1 | 5.0 | 0.0 | 0.0 | 2.3 | 0.0 | 3.2 | 4.2 | 0.0 | 0.0 | 0.1 | 43.7 | 15.5 | 137 | 133 | 15.0 | 2.9 |
| *Rosa hybrid cultivar* | *Spermatophyta* | RHD | 2378 | 2039 | 19.3 | 0.0 | 1.0 | 2.4 | 4.9 | 0.0 | 0.0 | 2.2 | 0.1 | 2.8 | 5.5 | 0.0 | 0.0 | 0.2 | 43.4 | 18.3 | 434 | 421 | 14.3 | 3.0 |
| *Saccharum sp.* | *Spermatophyta* | SSD | 6293 | 5003 | 16.4 | 0.0 | 0.6 | 2.0 | 3.5 | 0.0 | 0.0 | 1.4 | 0.0 | 2.0 | 4.6 | 0.0 | 0.2 | 0.2 | 33.4 | 35.5 | 2224 | 2050 | 20.3 | 7.8 |
| *Schedonorus arundinaceus* | *Spermatophyta* | SAC | 1132 | 990 | 11.7 | 0.0 | 0.6 | 1.9 | 3.6 | 0.0 | 0.0 | 3.2 | 0.1 | 2.1 | 2.6 | 0.0 | 0.0 | 0.1 | 32.8 | 41.3 | 467 | 426 | 12.5 | 8.8 |
| *Secale cereale* | *Spermatophyta* | SCD | 5323 | 4093 | 18.3 | 0.0 | 0.7 | 2.7 | 4.1 | 0.0 | 0.0 | 2.0 | 0.0 | 2.4 | 5.1 | 0.0 | 0.1 | 0.1 | 42.4 | 22.1 | 1173 | 1134 | 23.1 | 3.3 |
| *Sesamum indicum* | *Spermatophyta* | SIC | 1664 | 1427 | 24.2 | 0.0 | 0.9 | 2.6 | 3.7 | 0.0 | 0.0 | 2.3 | 0.0 | 2.8 | 4.9 | 0.0 | 0.0 | 0.2 | 41.0 | 17.3 | 287 | 281 | 14.2 | 2.1 |
| *Sorghum halepense* | *Spermatophyta* | SHC | 1073 | 973 | 14.7 | 0.0 | 0.7 | 2.0 | 2.4 | 0.0 | 0.0 | 0.3 | 0.0 | 1.6 | 3.4 | 0.0 | 0.1 | 0.2 | 35.1 | 39.4 | 423 | 414 | 9.3 | 2.1 |
| *Stevia rebaudiana* | *Spermatophyta* | SRD | 3155 | 2582 | 22.2 | 0.1 | 1.3 | 3.3 | 5.6 | 0.0 | 0.0 | 3.7 | 0.0 | 2.2 | 5.9 | 0.0 | 0.0 | 0.2 | 39.7 | 15.8 | 498 | 487 | 18.1 | 2.2 |
| *Suaeda maritima subsp. salsa* | *Spermatophyta* | SME | 506 | 478 | 13.4 | 0.0 | 1.0 | 3.0 | 4.2 | 0.0 | 0.2 | 2.6 | 0.0 | 3.2 | 3.6 | 0.0 | 0.2 | 0.8 | 38.1 | 29.8 | 151 | 147 | 5.5 | 2.6 |
| *Tamarix androssowii* | *Spermatophyta* | TAC | 1971 | 1819 | 5.4 | 0.0 | 0.4 | 0.7 | 1.8 | 0.0 | 0.1 | 0.8 | 0.1 | 1.1 | 2.0 | 0.1 | 0.1 | 0.6 | 17.7 | 69.2 | 1363 | 1273 | 7.7 | 6.6 |
| *Thellungiella halophila* | *Spermatophyta* | THC | 840 | 745 | 18.5 | 0.0 | 1.2 | 3.0 | 4.2 | 0.0 | 0.0 | 2.6 | 0.0 | 1.8 | 5.5 | 0.0 | 0.0 | 0.0 | 42.3 | 21.1 | 177 | 173 | 11.3 | 2.3 |
| *Theobroma cacao* | *Spermatophyta* | TCH | 2462 | 2136 | 15.1 | 0.0 | 0.8 | 2.1 | 3.4 | 0.0 | 0.1 | 2.3 | 0.0 | 2.0 | 4.5 | 0.0 | 0.0 | 0.3 | 42.3 | 27.2 | 668 | 636 | 13.2 | 4.8 |
| *Triticum monococcum* | *Spermatophyta* | TMD | 5796 | 4584 | 19.2 | 0.0 | 1.0 | 2.4 | 4.3 | 0.0 | 0.0 | 1.5 | 0.1 | 2.1 | 4.9 | 0.0 | 0.2 | 0.2 | 40.5 | 23.6 | 1362 | 1336 | 20.9 | 1.9 |
| *Triticum turgidum* | *Spermatophyta* | TTD | 5702 | 4325 | 18.6 | 0.1 | 1.2 | 2.2 | 4.3 | 0.0 | 0.0 | 2.1 | 0.2 | 2.9 | 5.1 | 0.0 | 0.9 | 0.2 | 40.5 | 21.7 | 1236 | 1128 | 24.1 | 8.7 |
| *Vaccinium corymbosum* | *Spermatophyta* | VCC | 955 | 888 | 20.8 | 0.0 | 0.9 | 2.7 | 6.9 | 0.0 | 0.0 | 3.0 | 0.1 | 2.7 | 5.5 | 0.0 | 0.0 | 0.1 | 34.8 | 22.3 | 213 | 203 | 7.0 | 4.7 |
| *Vitis aestivalis* | *Spermatophyta* | VAC | 1298 | 1085 | 21.6 | 0.0 | 0.8 | 3.6 | 4.7 | 0.0 | 0.1 | 4.4 | 0.0 | 2.1 | 5.3 | 0.0 | 0.0 | 0.0 | 46.1 | 11.2 | 146 | 144 | 16.4 | 1.4 |
| *Vitis hybrid cultivar* | *Spermatophyta* | VHD | 2065 | 1626 | 23.7 | 0.0 | 1.2 | 3.5 | 4.4 | 0.0 | 0.1 | 2.4 | 0.0 | 2.3 | 4.4 | 0.0 | 0.1 | 0.2 | 38.5 | 19.1 | 394 | 380 | 21.2 | 3.6 |
| *Vitis riparia* | *Spermatophyta* | VRC | 1048 | 482 | 20.6 | 0.0 | 0.6 | 3.2 | 4.8 | 0.0 | 0.2 | 1.1 | 0.0 | 2.3 | 6.5 | 0.0 | 0.0 | 0.0 | 45.0 | 15.6 | 82 | 80 | 8.0 | 2.4 |
| *Welwitschia mirabilis* | *Spermatophyta* | WMC | 2523 | 2266 | 15.5 | 0.0 | 0.7 | 2.2 | 4.0 | 0.0 | 0.0 | 1.9 | 0.1 | 2.1 | 4.4 | 0.2 | 0.1 | 0.2 | 28.6 | 40.0 | 1009 | 965 | 10.2 | 4.4 |
|  |  |  |  |  |  |  |  |  |  |  |  |  |  |  |  |  |  |  |  |  |  |  |  |  |
| *Fungi Species* | Taxa | *Species Identifier* | *Genes* | *GF* | *PFMV* | *PFM* | *PFV* | *PMV* | *FMV* | *PF* | *PM* | *PV* | *FM* | *FV* | *MV* | *P* | *F* | *M* | *V* | *SS (%)* | *SS (#)* | *GF of SS (#)* | *All Dup (%)* | *SS Dup (%)* |
| *Aspergillus niger* | *Ascomycota* | ANC | 1542 | 1471 | 14.3 | 0.3 | 0.8 | 0.1 | 3.9 | 0.5 | 0.0 | 0.1 | 1.8 | 3.2 | 0.1 | 0.0 | 17.3 | 0.4 | 0.1 | 57.1 | 881 | 855 | 4.6 | 3.0 |
| *Blumeria graminis f. sp. hordei* | *Ascomycota* | BGE | 2833 | 2504 | 13.1 | 0.3 | 0.5 | 0.3 | 4.9 | 0.5 | 0.0 | 0.1 | 2.7 | 3.1 | 0.3 | 0.0 | 15.6 | 0.5 | 0.5 | 57.6 | 1632 | 1436 | 11.6 | 12.0 |
| *Chaetomium globosum* | *Ascomycota* | CGC | 1328 | 1263 | 16.8 | 0.2 | 0.9 | 0.0 | 4.0 | 0.2 | 0.0 | 0.0 | 2.3 | 5.0 | 0.2 | 0.2 | 22.1 | 0.1 | 0.4 | 47.6 | 632 | 624 | 4.9 | 1.3 |
| *Coccidioides immitis* | *Ascomycota* | CIC | 1610 | 1528 | 19.4 | 0.6 | 1.5 | 0.2 | 6.3 | 0.6 | 0.0 | 0.0 | 3.5 | 3.7 | 0.2 | 0.1 | 39.9 | 0.6 | 0.6 | 22.8 | 366 | 361 | 5.0 | 1.4 |
| *Coccidioides posadasii* | *Ascomycota* | CPG | 5556 | 4782 | 16.0 | 0.9 | 1.1 | 0.5 | 6.5 | 0.6 | 0.1 | 0.1 | 3.0 | 4.1 | 1.1 | 0.1 | 23.2 | 1.3 | 1.0 | 40.5 | 2248 | 2084 | 13.9 | 7.3 |
| *Colletotrichum trifolii* | *Ascomycota* | CTC | 563 | 539 | 16.3 | 1.1 | 1.2 | 0.2 | 5.3 | 0.7 | 0.2 | 0.0 | 2.7 | 4.6 | 0.4 | 0.0 | 28.4 | 0.5 | 1.6 | 36.8 | 207 | 206 | 4.3 | 0.5 |
| *Cryphonectria parasitica* | *Ascomycota* | CPE | 2246 | 2034 | 21.4 | 0.4 | 1.3 | 0.2 | 6.7 | 0.5 | 0.1 | 0.0 | 3.7 | 5.5 | 0.3 | 0.0 | 22.8 | 0.4 | 0.5 | 36.1 | 811 | 759 | 9.4 | 6.4 |
| *Emericella nidulans* | *Ascomycota* | ENC | 7064 | 5828 | 14.1 | 0.6 | 1.2 | 0.3 | 4.8 | 0.6 | 0.1 | 0.0 | 2.8 | 3.8 | 0.4 | 0.1 | 20.6 | 0.4 | 0.7 | 49.5 | 3495 | 3230 | 17.5 | 7.6 |
| *Fusarium sporotrichioides* | *Ascomycota* | FSC | 3452 | 3278 | 11.8 | 0.5 | 0.6 | 0.1 | 3.8 | 0.7 | 0.0 | 0.1 | 3.0 | 4.9 | 0.4 | 0.0 | 35.1 | 0.6 | 0.7 | 37.7 | 1302 | 1291 | 5.0 | 0.8 |
| *Gibberella zeae* | *Ascomycota* | GZC | 4705 | 3746 | 19.0 | 0.7 | 1.0 | 0.2 | 5.2 | 0.5 | 0.0 | 0.0 | 3.0 | 6.6 | 0.5 | 0.0 | 26.1 | 0.5 | 0.9 | 35.8 | 1675 | 1527 | 20.2 | 8.8 |
| *Hypocrea lixii* | *Ascomycota* | HLC | 1744 | 1557 | 14.0 | 0.4 | 0.7 | 0.2 | 5.4 | 0.5 | 0.0 | 0.0 | 2.8 | 4.1 | 0.3 | 0.1 | 26.5 | 0.3 | 0.8 | 43.7 | 762 | 714 | 10.7 | 6.3 |
| *Metarhizium anisopliae* | *Ascomycota* | MAD | 1851 | 1712 | 16.2 | 0.8 | 0.9 | 0.4 | 4.8 | 0.6 | 0.0 | 0.1 | 2.8 | 5.3 | 0.3 | 0.1 | 26.6 | 0.7 | 0.5 | 39.8 | 737 | 719 | 7.5 | 2.4 |
| *Mycosphaerella graminicola* | *Ascomycota* | MGC | 2997 | 2821 | 18.5 | 0.7 | 1.6 | 0.2 | 6.7 | 0.7 | 0.1 | 0.1 | 3.0 | 4.3 | 0.7 | 0.1 | 20.4 | 0.7 | 2.1 | 40.2 | 1205 | 1164 | 5.9 | 3.4 |
| *Pneumocystis carinii f. sp. carinii* | *Ascomycota* | PCF | 1669 | 1406 | 19.1 | 0.8 | 0.8 | 1.0 | 7.1 | 0.5 | 0.1 | 0.1 | 3.9 | 1.8 | 1.6 | 0.0 | 8.6 | 4.4 | 1.0 | 49.3 | 821 | 711 | 15.8 | 13.4 |
| *Saccharomyces cerevisiae* | *Ascomycota* | SCC | 1757 | 1668 | 16.5 | 0.8 | 0.7 | 0.2 | 4.8 | 0.3 | 0.2 | 0.1 | 3.4 | 2.3 | 0.6 | 0.1 | 8.3 | 0.7 | 0.7 | 60.2 | 1057 | 1043 | 5.1 | 1.3 |
| *Schizosaccharomyces pombe* | *Ascomycota* | SPC | 2123 | 1861 | 20.6 | 0.5 | 0.5 | 0.2 | 3.3 | 0.2 | 0.1 | 0.0 | 1.9 | 2.1 | 0.3 | 0.0 | 5.8 | 0.6 | 0.4 | 63.3 | 1343 | 1285 | 12.3 | 4.3 |
| *Verticillium dahliae* | *Ascomycota* | VDC | 1315 | 1277 | 21.0 | 0.8 | 1.7 | 0.1 | 5.2 | 0.8 | 0.0 | 0.0 | 3.7 | 5.1 | 0.5 | 0.1 | 25.2 | 0.4 | 0.5 | 35.0 | 460 | 454 | 2.9 | 1.3 |
| *Cryptococcus neoformans var. neoformans* | *Basidiomycota* | CND | 3567 | 3040 | 21.7 | 0.8 | 1.1 | 0.4 | 5.9 | 0.3 | 0.1 | 0.1 | 3.3 | 4.0 | 0.7 | 0.1 | 12.2 | 1.1 | 0.9 | 47.3 | 1684 | 1569 | 14.7 | 6.8 |
| *Hebeloma cylindrosporum* | *Basidiomycota* | HCE | 2468 | 2249 | 20.6 | 0.6 | 1.3 | 0.3 | 5.1 | 0.5 | 0.0 | 0.0 | 3.2 | 4.0 | 0.7 | 0.8 | 12.8 | 1.2 | 0.9 | 47.7 | 1176 | 1112 | 8.8 | 5.4 |
| *Heterobasidion annosum* | *Basidiomycota* | HAE | 752 | 695 | 24.2 | 0.8 | 0.8 | 0.1 | 5.5 | 0.8 | 0.3 | 0.0 | 1.1 | 4.4 | 0.4 | 0.0 | 10.5 | 0.7 | 0.7 | 49.9 | 375 | 364 | 7.6 | 2.9 |
| *Laccaria bicolor* | *Basidiomycota* | LBC | 1106 | 1011 | 16.3 | 0.5 | 0.5 | 0.4 | 4.2 | 0.5 | 0.2 | 0.1 | 2.0 | 3.0 | 0.7 | 0.1 | 13.3 | 0.7 | 2.1 | 55.6 | 615 | 569 | 8.6 | 7.5 |
| *Paxillus involutus* | *Basidiomycota* | PID | 936 | 824 | 24.4 | 0.7 | 1.0 | 0.3 | 5.3 | 0.9 | 0.0 | 0.1 | 2.8 | 7.6 | 0.9 | 0.1 | 9.4 | 0.6 | 10.8 | 35.1 | 329 | 318 | 12.0 | 3.3 |
| *Pleurotus ostreatus* | *Basidiomycota* | POC | 1326 | 1243 | 20.8 | 0.5 | 0.9 | 0.3 | 6.4 | 0.3 | 0.1 | 0.1 | 3.3 | 3.8 | 0.8 | 0.0 | 16.5 | 1.0 | 1.1 | 44.0 | 584 | 554 | 6.3 | 5.1 |
| *Ustilago maydis* | *Basidiomycota* | UMC | 4621 | 3374 | 25.3 | 1.3 | 1.1 | 1.0 | 7.2 | 0.9 | 1.7 | 0.2 | 5.4 | 2.6 | 2.7 | 1.7 | 7.6 | 13.5 | 1.3 | 26.5 | 1223 | 1045 | 27.1 | 14.6 |
| *Glomus intraradices* | *Glomeromycota* | GIC | 2104 | 1828 | 13.5 | 0.2 | 0.7 | 0.9 | 3.5 | 0.2 | 0.6 | 0.1 | 1.5 | 1.4 | 1.1 | 0.5 | 4.5 | 1.9 | 1.2 | 68.1 | 1430 | 1297 | 13.0 | 9.3 |
| *Glomus versiforme* | *Glomeromycota* | GVC | 328 | 307 | 22.3 | 1.2 | 0.6 | 1.2 | 4.3 | 0.6 | 0.3 | 0.0 | 1.8 | 4.0 | 1.8 | 0.0 | 8.2 | 1.8 | 0.0 | 51.8 | 170 | 163 | 6.4 | 4.1 |
| *Conidiobolus coronatus* | *Zygomycota* | CCD | 965 | 919 | 19.3 | 0.9 | 0.9 | 0.7 | 5.5 | 0.6 | 0.3 | 0.1 | 3.4 | 2.8 | 1.2 | 0.1 | 8.1 | 1.7 | 1.0 | 53.3 | 514 | 497 | 4.9 | 3.3 |
|  |  |  |  |  |  |  |  |  |  |  |  |  |  |  |  |  |  |  |  |  |  |  |  |  |
| *Nematode Species* | *Taxa* | *Species Identifier* | *Genes* | *GF* | *PFMV* | *PFM* | *PFV* | *PMV* | *FMV* | *PF* | *PM* | *PV* | *FM* | *FV* | *MV* | *P* | *F* | *M* | *V* | *SS (%)* | *SS (#)* | *GF of SS (#)* | *All Dup (%)* | *SS Dup (%)* |
| *Trichuris muris* | *Dorylaimia* | TMC | 1593 | 1363 | 18.3 | 0.7 | 0.1 | 2.4 | 2.8 | 0.0 | 1.1 | 0.1 | 1.9 | 0.1 | 4.0 | 0.1 | 0.0 | 21.3 | 0.3 | 46.8 | 744 | 638 | 14.4 | 14.2 |
| *Trichinella spiralis* | *Dorylaimia* | TSC | 3716 | 3082 | 16.3 | 0.6 | 0.0 | 1.8 | 3.8 | 0.1 | 1.2 | 0.0 | 2.2 | 0.1 | 3.8 | 0.1 | 0.2 | 18.8 | 0.4 | 50.5 | 1877 | 1689 | 17.1 | 10.0 |
| *Ascaris lumbricoides* | *Spirurina* | ALC | 879 | 642 | 12.5 | 0.2 | 0.0 | 1.4 | 2.5 | 0.0 | 0.3 | 0.1 | 0.6 | 0.0 | 2.6 | 0.0 | 0.0 | 54.0 | 0.0 | 25.7 | 226 | 211 | 27.0 | 6.6 |
| *Ascaris suum* | *Spirurina* | ASC | 8922 | 5410 | 15.2 | 0.5 | 0.0 | 1.5 | 4.1 | 0.0 | 0.7 | 0.0 | 1.2 | 0.0 | 2.6 | 0.0 | 0.0 | 31.7 | 0.0 | 42.3 | 3766 | 3409 | 39.3 | 9.5 |
| *Brugia malayi* | *Spirurina* | BMC | 9650 | 7311 | 11.4 | 0.4 | 0.0 | 1.3 | 2.2 | 0.0 | 0.7 | 0.0 | 1.5 | 0.1 | 2.7 | 0.1 | 0.1 | 28.5 | 0.5 | 50.7 | 4865 | 4486 | 24.0 | 7.8 |
| *Dirofilaria immitis* | *Spirurina* | DIC | 1759 | 1529 | 14.6 | 0.7 | 0.0 | 1.3 | 3.9 | 0.0 | 0.6 | 0.0 | 1.3 | 0.0 | 3.1 | 0.0 | 0.0 | 32.9 | 0.3 | 41.3 | 723 | 686 | 12.9 | 5.1 |
| *Onchocerca volvulus* | *Spirurina* | OVC | 5152 | 4060 | 12.7 | 0.5 | 0.1 | 1.7 | 2.0 | 0.0 | 0.7 | 0.0 | 1.2 | 0.1 | 3.1 | 0.1 | 0.1 | 28.4 | 0.6 | 48.7 | 2461 | 2294 | 20.5 | 6.8 |
| *Toxocara canis* | *Spirurina* | TCC | 1500 | 943 | 19.5 | 0.4 | 0.0 | 1.9 | 2.9 | 0.0 | 0.9 | 0.0 | 0.8 | 0.0 | 3.3 | 0.1 | 0.0 | 38.8 | 0.1 | 31.3 | 464 | 410 | 36.9 | 11.6 |
| *Wuchereria bancrofti* | *Spirurina* | WBC | 1804 | 1528 | 21.5 | 0.7 | 0.0 | 3.5 | 3.3 | 0.0 | 1.1 | 0.0 | 1.2 | 0.0 | 4.5 | 0.1 | 0.1 | 33.3 | 0.6 | 30.3 | 539 | 530 | 15.0 | 1.7 |
| *Globodera pallida* | *Tylenchida* | GPC | 984 | 721 | 16.2 | 0.1 | 0.0 | 1.3 | 2.5 | 0.0 | 0.8 | 0.0 | 0.9 | 0.0 | 4.0 | 0.0 | 0.1 | 27.4 | 0.6 | 46.0 | 442 | 360 | 25.9 | 18.6 |
| *Globodera rostochiensis* | *Tylenchida* | GRC | 2873 | 2482 | 18.7 | 0.5 | 0.0 | 2.2 | 4.3 | 0.0 | 1.4 | 0.0 | 2.3 | 0.1 | 4.1 | 0.0 | 0.1 | 38.5 | 0.1 | 27.7 | 797 | 754 | 13.6 | 5.4 |
| *Heterodera glycines* | *Tylenchida* | HGC | 7318 | 5644 | 17.1 | 0.7 | 0.0 | 1.9 | 4.4 | 0.0 | 1.2 | 0.0 | 1.8 | 0.2 | 3.8 | 0.1 | 0.2 | 34.1 | 0.4 | 34.1 | 2495 | 2290 | 22.9 | 8.2 |
| *Heterodera schachtii* | *Tylenchida* | HSC | 1364 | 1147 | 16.3 | 0.7 | 0.1 | 1.9 | 4.4 | 0.0 | 1.6 | 0.0 | 2.4 | 0.1 | 4.0 | 0.0 | 0.0 | 42.9 | 0.4 | 25.1 | 343 | 322 | 15.9 | 6.1 |
| *Meloidogyne arenaria* | *Tylenchida* | MAC | 1951 | 1667 | 17.9 | 0.7 | 0.0 | 2.1 | 3.5 | 0.0 | 1.4 | 0.0 | 1.7 | 0.1 | 4.0 | 0.0 | 0.1 | 49.8 | 0.0 | 18.9 | 363 | 356 | 14.3 | 1.9 |
| *Meloidogyne chitwoodi* | *Tylenchida* | MCC | 2440 | 2042 | 14.5 | 0.6 | 0.0 | 1.7 | 4.1 | 0.0 | 0.7 | 0.0 | 1.8 | 0.0 | 3.2 | 0.0 | 0.0 | 40.9 | 0.1 | 32.3 | 786 | 734 | 16.3 | 6.6 |
| *Meloidogyne hapla* | *Tylenchida* | MHC | 4522 | 3566 | 15.3 | 0.7 | 0.0 | 1.7 | 3.6 | 0.0 | 1.2 | 0.0 | 1.7 | 0.0 | 3.7 | 0.0 | 0.1 | 43.6 | 0.1 | 28.2 | 1272 | 1196 | 21.1 | 6.0 |
| *Meloidogyne incognita* | *Tylenchida* | MIC | 4589 | 3585 | 14.4 | 0.7 | 0.0 | 1.8 | 3.6 | 0.0 | 1.3 | 0.0 | 2.0 | 0.1 | 3.6 | 0.0 | 0.0 | 47.0 | 0.0 | 25.4 | 1162 | 1114 | 21.8 | 4.1 |
| *Meloidogyne javanica* | *Tylenchida* | MJC | 2743 | 2233 | 14.4 | 0.4 | 0.0 | 1.9 | 2.9 | 0.0 | 1.2 | 0.0 | 1.6 | 0.0 | 3.7 | 0.0 | 0.1 | 48.0 | 0.1 | 25.7 | 702 | 662 | 18.5 | 5.7 |
| *Meloidogyne paranaensis* | *Tylenchida* | MPE | 1142 | 1046 | 20.5 | 0.3 | 0.0 | 1.4 | 3.8 | 0.0 | 0.8 | 0.0 | 1.7 | 0.0 | 4.5 | 0.0 | 0.1 | 46.1 | 0.0 | 21.0 | 240 | 234 | 8.4 | 2.5 |
| *Parastrongyloides trichosuri* | *Tylenchida* | PTC | 3140 | 2363 | 22.3 | 0.5 | 0.0 | 1.9 | 3.3 | 0.0 | 1.3 | 0.0 | 2.2 | 0.1 | 2.8 | 0.0 | 0.1 | 32.6 | 0.0 | 32.8 | 1030 | 946 | 24.7 | 8.2 |
| *Pratylenchus penetrans* | *Tylenchida* | PEC | 417 | 354 | 28.1 | 0.0 | 0.0 | 1.4 | 4.8 | 0.0 | 0.7 | 0.0 | 2.2 | 0.0 | 4.1 | 0.0 | 0.0 | 34.1 | 0.5 | 24.2 | 101 | 97 | 15.1 | 4.0 |
| *Strongyloides ratti* | *Tylenchida* | SRC | 3298 | 2760 | 20.8 | 0.7 | 0.0 | 2.1 | 3.4 | 0.0 | 1.5 | 0.0 | 1.8 | 0.1 | 4.1 | 0.0 | 0.2 | 33.3 | 0.2 | 32.0 | 1052 | 1015 | 16.3 | 3.5 |
| *Strongyloides stercoralis* | *Tylenchida* | SSC | 3686 | 2902 | 22.0 | 1.2 | 0.0 | 2.2 | 5.3 | 0.0 | 1.3 | 0.0 | 2.6 | 0.0 | 5.8 | 0.1 | 0.2 | 33.4 | 0.3 | 25.6 | 944 | 883 | 21.3 | 6.5 |
| *Zeldia punctata* | *Tylenchida* | ZPC | 209 | 181 | 48.8 | 0.0 | 0.5 | 4.3 | 1.9 | 0.5 | 1.4 | 0.0 | 2.9 | 0.0 | 1.9 | 0.0 | 0.0 | 25.8 | 0.0 | 12.0 | 25 | 25 | 13.4 | 0.0 |
| *Ancylostoma ceylanicum* | *Rhabditina* | AYC | 3711 | 2797 | 20.5 | 0.8 | 0.0 | 2.1 | 3.7 | 0.0 | 1.1 | 0.0 | 1.8 | 0.0 | 3.7 | 0.0 | 0.1 | 39.3 | 0.2 | 26.8 | 996 | 883 | 24.6 | 11.3 |
| *Haemonchus contortus* | *Rhabditina* | HCC | 4709 | 2935 | 21.2 | 0.6 | 0.0 | 3.3 | 4.5 | 0.0 | 1.1 | 0.0 | 1.9 | 0.0 | 4.2 | 0.0 | 0.0 | 37.7 | 0.1 | 25.4 | 1183 | 955 | 37.5 | 19.3 |
| *Necator americanus* | *Rhabditina* | NAC | 2328 | 1868 | 13.8 | 0.3 | 0.0 | 1.2 | 2.7 | 0.0 | 0.6 | 0.0 | 1.2 | 0.0 | 3.0 | 0.0 | 0.0 | 37.4 | 0.0 | 39.5 | 917 | 817 | 19.7 | 10.9 |
| *Nippostrongylus brasiliensis* | *Rhabditina* | NBC | 775 | 629 | 21.7 | 0.1 | 0.0 | 2.6 | 3.5 | 0.0 | 0.9 | 0.0 | 1.5 | 0.0 | 3.4 | 0.0 | 0.1 | 33.9 | 0.0 | 32.3 | 250 | 209 | 18.8 | 16.4 |
| *Ostertagia ostertagi* | *Rhabditina* | OOC | 2564 | 1932 | 17.0 | 1.2 | 0.0 | 1.8 | 3.8 | 0.1 | 1.5 | 0.0 | 6.0 | 0.1 | 4.1 | 0.2 | 1.3 | 43.3 | 0.3 | 19.3 | 489 | 453 | 24.5 | 7.4 |
| *Pristionchus pacificus* | *Rhabditina* | PPC | 3724 | 3045 | 21.0 | 0.6 | 0.1 | 2.1 | 4.9 | 0.0 | 0.8 | 0.0 | 1.9 | 0.1 | 3.5 | 0.1 | 0.1 | 23.4 | 0.2 | 41.3 | 1534 | 1486 | 18.2 | 3.1 |
| *Teladorsagia circumcincta* | *Rhabditina* | TDC | 1879 | 1329 | 15.9 | 0.2 | 0.0 | 1.5 | 2.1 | 0.0 | 1.2 | 0.0 | 1.3 | 0.0 | 3.3 | 0.0 | 0.1 | 47.5 | 0.1 | 26.7 | 502 | 401 | 29.3 | 20.1 |
|  |  |  |  |  |  |  |  |  |  |  |  |  |  |  |  |  |  |  |  |  |  |  |  |  |
| *Arthropod / Tardigrade Species* | *Taxon* | *Species Identifier* | *Genes* | *GF* | *PFMV* | *PFM* | *PFV* | *PMV* | *FMV* | *PF* | *PM* | *PV* | *FM* | *FV* | *MV* | *P* | *F* | *M* | *V* | *SS (%)* | *SS (#)* | *GF of SS (#)* | *All Dup (%)* | *SS Dup (%)* |
| *Amblyomma americanum* | *Chelicerata* | AAC | 1440 | 1379 | 13.6 | 0.5 | 0.1 | 1.0 | 2.7 | 0.0 | 1.0 | 0.1 | 1.3 | 0.0 | 2.3 | 0.1 | 0.0 | 16.0 | 0.1 | 61.4 | 884 | 864 | 4.2 | 2.3 |
| *Amblyomma variegatum* | *Chelicerata* | AVC | 2158 | 1884 | 17.8 | 0.6 | 0.1 | 2.2 | 4.1 | 0.0 | 1.5 | 0.0 | 2.3 | 0.1 | 4.9 | 0.0 | 0.3 | 20.5 | 0.5 | 45.1 | 974 | 894 | 12.7 | 8.2 |
| *Ornithodoros porcinus porcinus* | *Chelicerata* | OPD | 759 | 608 | 13.0 | 1.3 | 0.0 | 1.3 | 3.7 | 0.1 | 1.2 | 0.0 | 1.4 | 0.0 | 1.8 | 0.0 | 0.0 | 13.4 | 0.8 | 61.8 | 445 | 370 | 17.3 | 16.9 |
| *Sarcoptes scabiei* | *Chelicerata* | SSE | 766 | 692 | 16.7 | 0.8 | 0.0 | 1.2 | 2.7 | 0.0 | 1.2 | 0.1 | 2.3 | 0.1 | 3.5 | 0.0 | 0.1 | 14.2 | 0.7 | 56.3 | 427 | 383 | 9.2 | 10.3 |
| *Litopenaeus setiferus* | *Crustacea* | LSE | 518 | 446 | 24.5 | 1.2 | 0.0 | 1.4 | 1.5 | 0.0 | 1.7 | 0.0 | 1.2 | 0.0 | 1.9 | 0.2 | 0.0 | 36.3 | 0.0 | 30.1 | 156 | 151 | 13.9 | 3.2 |
| *Litopenaeus vannamei* | *Crustacea* | LVC | 2978 | 2694 | 12.9 | 0.4 | 0.0 | 1.4 | 1.8 | 0.0 | 1.2 | 0.0 | 1.3 | 0.0 | 1.9 | 0.1 | 0.0 | 15.0 | 0.1 | 63.7 | 1896 | 1824 | 9.5 | 3.8 |
| *Marsupenaeus japonicus* | *Crustacea* | MJD | 545 | 440 | 16.0 | 0.6 | 0.0 | 2.2 | 1.3 | 0.0 | 1.1 | 0.0 | 0.2 | 0.0 | 0.7 | 0.0 | 0.0 | 18.0 | 0.0 | 60.0 | 325 | 302 | 19.0 | 7.1 |
| *Acyrthosiphon pisum* | *Insecta* | APC | 743 | 679 | 36.6 | 0.5 | 0.1 | 2.7 | 4.3 | 0.0 | 1.1 | 0.3 | 1.7 | 0.1 | 5.0 | 0.1 | 0.1 | 20.9 | 1.1 | 25.3 | 188 | 184 | 8.6 | 2.1 |
| *Aedes aegypti* | *Insecta* | AAD | 2380 | 2126 | 17.5 | 1.4 | 0.1 | 1.3 | 3.7 | 0.0 | 2.2 | 0.0 | 2.2 | 0.1 | 3.1 | 0.6 | 0.2 | 12.7 | 0.3 | 54.5 | 1298 | 1246 | 10.7 | 4.0 |
| *Ctenocephalides felis* | *Insecta* | CFC | 1386 | 1172 | 19.6 | 2.0 | 0.1 | 1.9 | 3.7 | 0.0 | 2.0 | 0.1 | 2.3 | 0.1 | 3.9 | 0.2 | 0.1 | 16.8 | 0.4 | 46.8 | 647 | 576 | 15.3 | 11.0 |
| *Culicoides sonorensis* | *Insecta* | CSD | 771 | 634 | 25.8 | 5.6 | 0.0 | 3.5 | 3.4 | 0.0 | 2.6 | 0.0 | 2.3 | 0.0 | 3.1 | 1.3 | 0.0 | 15.4 | 0.3 | 36.7 | 283 | 258 | 17.8 | 8.8 |
| *Ips pini* | *Insecta* | IPC | 592 | 435 | 29.7 | 2.0 | 0.2 | 4.4 | 4.7 | 0.0 | 2.7 | 0.0 | 1.9 | 0.3 | 4.1 | 0.0 | 0.3 | 14.7 | 1.0 | 34.0 | 198 | 132 | 26.1 | 33.3 |
| *Manduca sexta* | *Insecta* | MSC | 1370 | 1244 | 19.5 | 1.2 | 0.0 | 2.2 | 2.1 | 0.0 | 1.1 | 0.0 | 1.6 | 0.0 | 2.6 | 0.1 | 0.2 | 14.8 | 0.4 | 54.1 | 741 | 691 | 9.2 | 6.7 |
| *Pediculus humanus* | *Insecta* | PHC | 457 | 397 | 28.9 | 2.2 | 0.0 | 2.4 | 4.4 | 0.0 | 3.9 | 0.2 | 1.1 | 0.0 | 5.0 | 0.0 | 0.0 | 14.9 | 0.0 | 37.0 | 168 | 136 | 12.9 | 19.0 |
| *Toxoptera citricida* | *Insecta* | TCE | 2134 | 1993 | 19.5 | 0.7 | 0.0 | 1.5 | 3.0 | 0.0 | 1.5 | 0.0 | 2.2 | 0.1 | 4.2 | 0.1 | 0.1 | 19.4 | 0.3 | 47.2 | 1008 | 991 | 6.6 | 1.7 |
| *Tribolium castaneum* | *Insecta* | TCF | 1767 | 1477 | 22.1 | 0.8 | 0.0 | 1.8 | 2.8 | 0.0 | 1.2 | 0.0 | 1.5 | 0.1 | 3.2 | 0.2 | 0.2 | 14.2 | 0.3 | 51.7 | 913 | 849 | 16.4 | 7.0 |
| *Hypsibius dujardini* | *Tardigrada* | HDC | 1764 | 1521 | 19.6 | 0.7 | 0.0 | 2.0 | 3.7 | 0.0 | 1.0 | 0.1 | 1.9 | 0.6 | 4.6 | 0.1 | 0.4 | 12.9 | 6.9 | 45.5 | 802 | 729 | 13.8 | 9.1 |
|  |  |  |  |  |  |  |  |  |  |  |  |  |  |  |  |  |  |  |  |  |  |  |  |  |
| *Lophotrochozoan Species* | *Taxa* | *Species Identifier* | *Genes* | *GF* | *PFMV* | *PFM* | *PFV* | *PMV* | *FMV* | *PF* | *PM* | *PV* | *FM* | *FV* | *MV* | *P* | *F* | *M* | *V* | *SS (%)* | *SS (#)* | *GF of SS (#)* | *All Dup (%)* | *SS Dup (%)* |
| *Biomphalaria glabrata* | *Mollusca* | BGC | 2984 | 2555 | 8.4 | 0.1 | 0.1 | 1.1 | 1.6 | 0.1 | 1.4 | 0.0 | 0.7 | 0.2 | 1.3 | 0.4 | 0.1 | 8.9 | 0.2 | 75.4 | 2249 | 1966 | 14.3 | 12.6 |
| *Crassostrea gigas* | *Mollusca* | CGD | 1282 | 1182 | 17.2 | 0.4 | 0.0 | 1.7 | 2.3 | 0.0 | 1.2 | 0.0 | 1.3 | 0.1 | 2.7 | 0.2 | 0.1 | 22.2 | 0.5 | 50.1 | 641 | 623 | 7.7 | 2.8 |
| *Crassostrea virginica* | *Mollusca* | CVC | 2707 | 2420 | 13.5 | 0.4 | 0.0 | 1.6 | 2.4 | 0.0 | 1.2 | 0.0 | 1.3 | 0.1 | 2.1 | 0.1 | 0.3 | 17.1 | 0.3 | 59.6 | 1612 | 1533 | 10.6 | 4.9 |
| *Dugesia japonica* | *Platyhelminth* | DJC | 3392 | 2814 | 19.9 | 0.6 | 0.1 | 2.2 | 3.9 | 0.0 | 1.4 | 0.1 | 2.2 | 0.1 | 3.7 | 0.1 | 0.2 | 17.0 | 0.7 | 47.8 | 1617 | 1483 | 16.9 | 8.3 |
|  |  |  |  |  |  |  |  |  |  |  |  |  |  |  |  |  |  |  |  |  |  |  |  |  |
| *Deuterostome Species* | *Taxa* | *Species Identifier* | *Genes* | *GF* | *PFMV* | *PFM* | *PFV* | *PMV* | *FMV* | *PF* | *PM* | *PV* | *FM* | *FV* | *MV* | *P* | *F* | *M* | *V* | *SS* | *SS (#)* | *GF of SS (#)* | *All Dup (%)* | *SS Dup (%)* |
| *Ambystoma mexicanum* | *Tetrapoda* | AMF | 5422 | 4226 | 19.2 | 0.5 | 0.0 | 1.8 | 3.3 | 0.0 | 2.3 | 0.0 | 1.6 | 0.1 | 2.9 | 0.1 | 0.1 | 35.1 | 0.1 | 32.9 | 1784 | 1735 | 22.1 | 2.7 |
| *Ambystoma tigrinum tigrinum* | *Tetrapoda* | ATE | 10299 | 7832 | 14.5 | 0.5 | 0.0 | 1.7 | 3.0 | 0.0 | 1.5 | 0.0 | 1.6 | 0.1 | 3.5 | 0.1 | 0.1 | 30.5 | 0.2 | 42.7 | 4400 | 4097 | 23.9 | 6.9 |
| *Anolis sagrei* | *Tetrapoda* | ASD | 2124 | 1790 | 5.1 | 0.3 | 0.0 | 0.4 | 1.5 | 0.0 | 0.8 | 0.0 | 0.9 | 0.0 | 1.0 | 0.0 | 0.1 | 9.0 | 0.0 | 80.7 | 1714 | 1513 | 15.7 | 11.7 |
| *Bos indicus* | *Tetrapoda* | BIC | 1618 | 1380 | 6.9 | 0.4 | 0.3 | 2.0 | 2.0 | 0.3 | 1.5 | 0.1 | 0.9 | 0.4 | 1.2 | 0.4 | 0.4 | 21.3 | 1.3 | 60.6 | 980 | 919 | 14.7 | 6.2 |
| *Equus caballus* | *Tetrapoda* | ECD | 5990 | 4561 | 12.0 | 0.5 | 0.0 | 1.4 | 2.2 | 0.0 | 1.3 | 0.0 | 1.6 | 0.1 | 3.2 | 0.4 | 0.2 | 27.2 | 0.2 | 49.9 | 2984 | 2576 | 23.8 | 13.7 |
| *Oryctolagus cuniculus* | *Tetrapoda* | OCC | 1417 | 706 | 36.5 | 0.7 | 0.0 | 1.7 | 2.0 | 0.0 | 0.5 | 0.0 | 0.9 | 0.0 | 4.8 | 0.0 | 0.1 | 18.5 | 0.2 | 34.2 | 483 | 415 | 50.1 | 14.1 |
| *Ovis aries* | *Tetrapoda* | OAC | 4348 | 3823 | 10.9 | 0.3 | 0.0 | 1.1 | 1.7 | 0.0 | 2.2 | 0.0 | 1.1 | 0.0 | 2.4 | 0.3 | 0.1 | 25.8 | 0.1 | 53.8 | 2337 | 2258 | 12.0 | 3.4 |
| *Pan troglodytes* | *Tetrapoda* | PTJ | 3108 | 2540 | 15.7 | 0.5 | 0.0 | 5.3 | 2.7 | 0.0 | 1.8 | 0.0 | 1.5 | 0.0 | 4.0 | 0.1 | 0.2 | 26.1 | 0.3 | 41.7 | 1294 | 1252 | 18.2 | 3.2 |
| *Taeniopygia guttata* | *Tetrapoda* | TGD | 10647 | 9300 | 10.8 | 0.4 | 0.0 | 1.2 | 2.8 | 0.0 | 1.1 | 0.1 | 1.5 | 0.1 | 3.2 | 0.1 | 0.1 | 17.9 | 0.4 | 60.3 | 6422 | 6090 | 12.6 | 5.2 |
| *Tupaia belangeri* | *Tetrapoda* | TBE | 1447 | 1365 | 5.3 | 1.0 | 0.0 | 1.4 | 1.5 | 0.0 | 1.3 | 0.0 | 0.8 | 0.0 | 1.3 | 0.1 | 0.1 | 19.8 | 0.3 | 67.2 | 972 | 944 | 5.7 | 2.9 |
| *Coregonus clupeaformis* | *Teleost* | CCC | 913 | 828 | 10.0 | 0.2 | 0.0 | 0.9 | 1.1 | 0.0 | 1.2 | 0.0 | 0.9 | 0.1 | 0.2 | 0.1 | 0.0 | 20.2 | 0.1 | 65.1 | 593 | 572 | 9.2 | 3.5 |
| *Cyprinus carpio* | *Teleost* | CCE | 7922 | 5609 | 17.0 | 0.8 | 0.1 | 1.8 | 3.0 | 0.0 | 2.2 | 0.0 | 2.0 | 0.0 | 3.2 | 0.0 | 0.1 | 28.4 | 0.2 | 41.4 | 3278 | 2945 | 29.2 | 10.2 |
| *Gasterosteus aculeatus* | *Teleost* | GAC | 9205 | 5925 | 21.0 | 1.1 | 0.0 | 2.2 | 3.9 | 0.0 | 2.0 | 0.0 | 2.4 | 0.1 | 4.1 | 0.1 | 0.1 | 26.5 | 0.3 | 36.0 | 3317 | 2891 | 35.6 | 12.8 |
| *Ictalurus furcatus* | *Teleost* | IFC | 7254 | 4976 | 10.1 | 1.0 | 0.0 | 0.8 | 1.6 | 0.0 | 1.4 | 0.0 | 1.4 | 0.0 | 1.4 | 0.2 | 0.0 | 19.5 | 0.3 | 62.4 | 4522 | 3702 | 31.4 | 18.1 |
| *Oncorhynchus tshawytscha* | *Teleost* | OTC | 881 | 825 | 9.4 | 0.5 | 0.0 | 0.3 | 0.7 | 0.0 | 1.0 | 0.0 | 0.9 | 0.0 | 1.5 | 0.0 | 0.0 | 20.2 | 0.0 | 65.5 | 577 | 567 | 6.4 | 1.7 |
| *Paralichthys olivaceus* | *Teleost* | POD | 1907 | 1698 | 16.7 | 0.6 | 0.1 | 0.9 | 2.1 | 0.0 | 2.1 | 0.0 | 1.5 | 0.0 | 2.1 | 0.1 | 0.1 | 27.5 | 0.2 | 46.0 | 878 | 851 | 11.0 | 3.1 |
| *Pseudopleuronectes americanus* | *Teleost* | PAC | 889 | 831 | 16.6 | 0.4 | 0.0 | 1.3 | 1.9 | 0.0 | 1.3 | 0.0 | 1.2 | 0.1 | 2.9 | 0.0 | 0.0 | 34.6 | 0.1 | 39.3 | 349 | 344 | 6.5 | 1.4 |
| *Branchiostoma floridae* | *Cephalochordata* | BFC | 8243 | 6160 | 9.6 | 0.7 | 0.0 | 1.0 | 2.0 | 0.0 | 1.0 | 0.0 | 1.1 | 0.1 | 1.7 | 0.1 | 0.1 | 9.2 | 0.3 | 73.0 | 6019 | 4761 | 25.3 | 20.9 |
| *Halocynthia roretzi* | *Urochordata* | HRC | 2825 | 2696 | 10.4 | 0.5 | 0.1 | 1.0 | 3.6 | 0.0 | 0.9 | 0.1 | 2.1 | 0.3 | 3.3 | 0.0 | 0.3 | 15.7 | 0.4 | 61.3 | 1730 | 1688 | 4.5 | 2.4 |
| *Molgula tectiformis* | *Urochordata* | MTC | 2472 | 2240 | 11.7 | 1.0 | 0.0 | 1.1 | 2.4 | 0.0 | 0.9 | 0.0 | 1.5 | 0.1 | 2.5 | 0.0 | 0.2 | 15.9 | 0.4 | 62.2 | 1537 | 1437 | 9.3 | 6.5 |
| *Hemicentrotus pulcherrimus* | *Echinodermata* | HPC | 1312 | 1210 | 12.3 | 0.3 | 0.2 | 0.4 | 1.0 | 0.0 | 0.6 | 0.0 | 0.5 | 0.1 | 1.0 | 0.0 | 0.1 | 5.2 | 0.2 | 78.2 | 1026 | 966 | 7.8 | 5.8 |
