# Supplementary material for: The global landscape of sequence diversity
Source: Genome Biol. 2007 Nov 8;8(11):R238. doi: 10.1186/gb-2007-8-11-r238 (PMC2258180; doi:10.1186/gb-2007-8-11-r238)
Supplement: Additional data file 4 — Comparison of sequence similarity relationships between members of 16 highly conserved gene families for eukaryotes and bacteria. [file gb-2007-8-11-r238-S4.pdf]

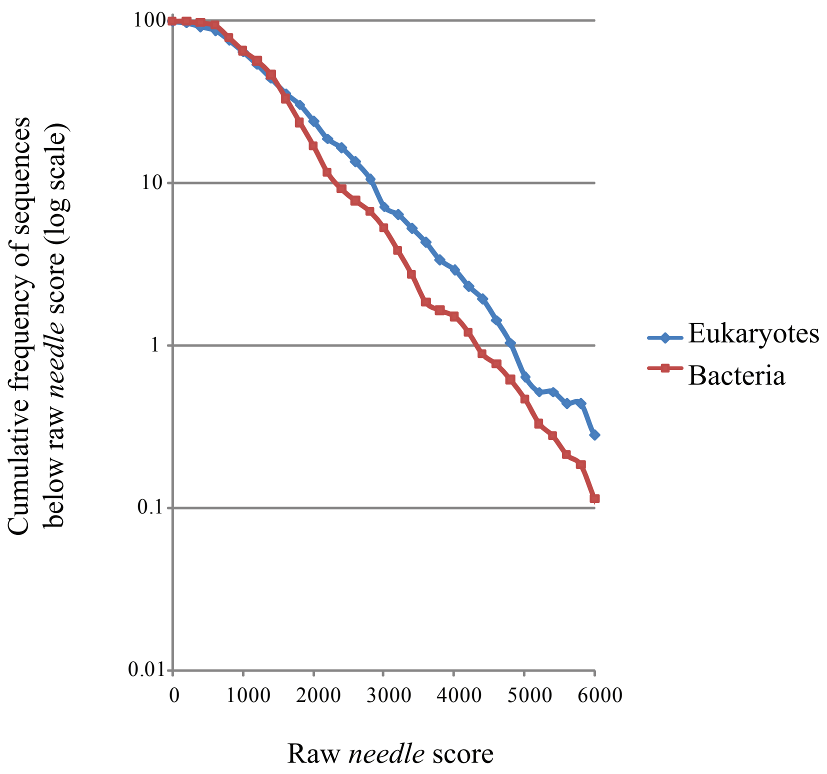

Figure S2. Distribution of sequence similarity scores for 20 highly conserved gene families across sequences drived from complete genomes

As a simple test to examine the relative diversity of sequences from the bacterial and eukaryotic complete genomes, we determined the distribution of sequence similarity scores across 16 highly conserved gene families as defined by COGENT and discussed in the manuscript (TR000038; TR000050; TR000077; TR000095; TR000100; TR000139; TR000155; TR000178; TR000213; TR000223; TR000266; TR000296; TR000339; TR000352; TR000443; TR000575). For each of the 19 eukaryotic genomes every member of the 16 selected gene families was compared to all other gene family members from every other eukaryotic genome using the EMBOSS package - *needle*. For bacteria, similar searches were performed for the same 16 gene families using 400 random samples of 19 bacterial genomes. For each genome comparison, the highest scoring pair of sequences was used to derive a single *needle* score for that genome comparison (this results in a maximum of 2736 comparisons for the 16 gene familes associated with each set of 19 genomes). The graph shows the cumulative frequency of sequence comparison scores below the indicated *needle* score. It should be obvious is that there is a higher frequency of sequence matches with higher scores for eukaryotes than for the bacterial datasets. For example 18.9% of eukaryote comparisons have a needle score greater than 2200 compared with only 11.7% of bacterial sequences. We suggest that these observed differences indicate the closer evolutionary relationships of the eukaryotic genomes compared with the randomly selected samples of bacterial genomes.
