# Supplementary material for: The global landscape of sequence diversity
Source: Genome Biol. 2007 Nov 8;8(11):R238. doi: 10.1186/gb-2007-8-11-r238 (PMC2258180; doi:10.1186/gb-2007-8-11-r238)
Supplement: Additional data file 5 — Rates of sequence and gene family discovery in different sets of complete and partial genomes. [file gb-2007-8-11-r238-S5.pdf]

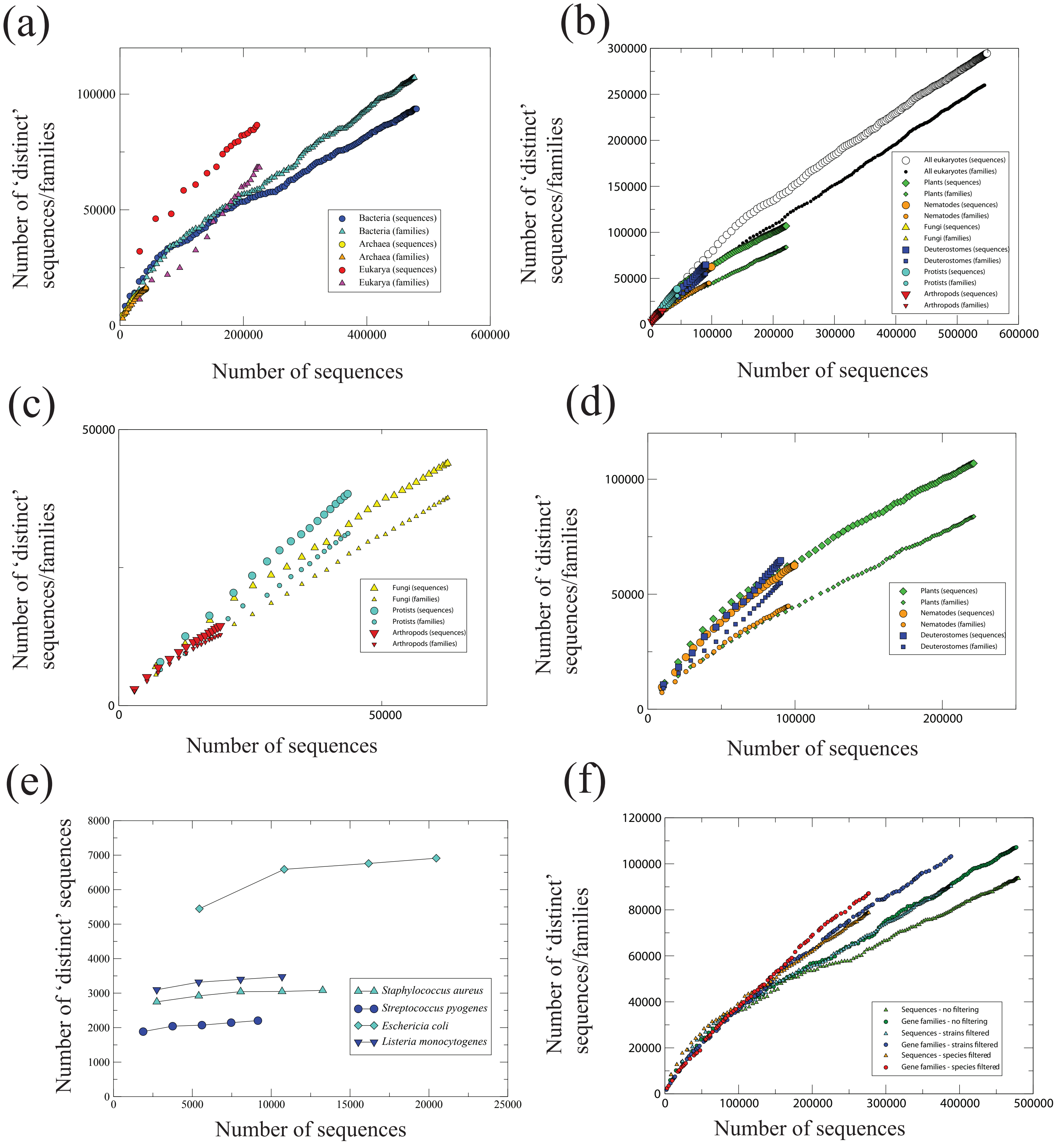

Figure S3. Analysis of sequence and gene family discovery rates

(a) Discovery of distinct sequences and gene families in all complete genomes ordered by the number of sequences / gene families (largest first). (b-d) Discovery of distinct sequences and gene families in partial genomes ordered by the number of sequences (largest first) and organized into distinct taxonomic groups. (e) Sequence discovery in strains associated with four bacterial species. (f) Discovery of distinct sequences and gene families in bacterial genomes ordered by the number of sequences (largest first). Three sets are presented: all bacterial genomes; bacterial genomes from which strains from identical species have been removed (keeping the strain with the largest number of sequences); and bacterial genomes from which species from identical genera have been removed (again keeping the species with the largest number of sequences).
