# Supplementary material for: The global landscape of sequence diversity
Source: Genome Biol. 2007 Nov 8;8(11):R238. doi: 10.1186/gb-2007-8-11-r238 (PMC2258180; doi:10.1186/gb-2007-8-11-r238)
Supplement: Additional data file 6 — Relationship between genome size and number of species-specific sequences. [file gb-2007-8-11-r238-S6.pdf]

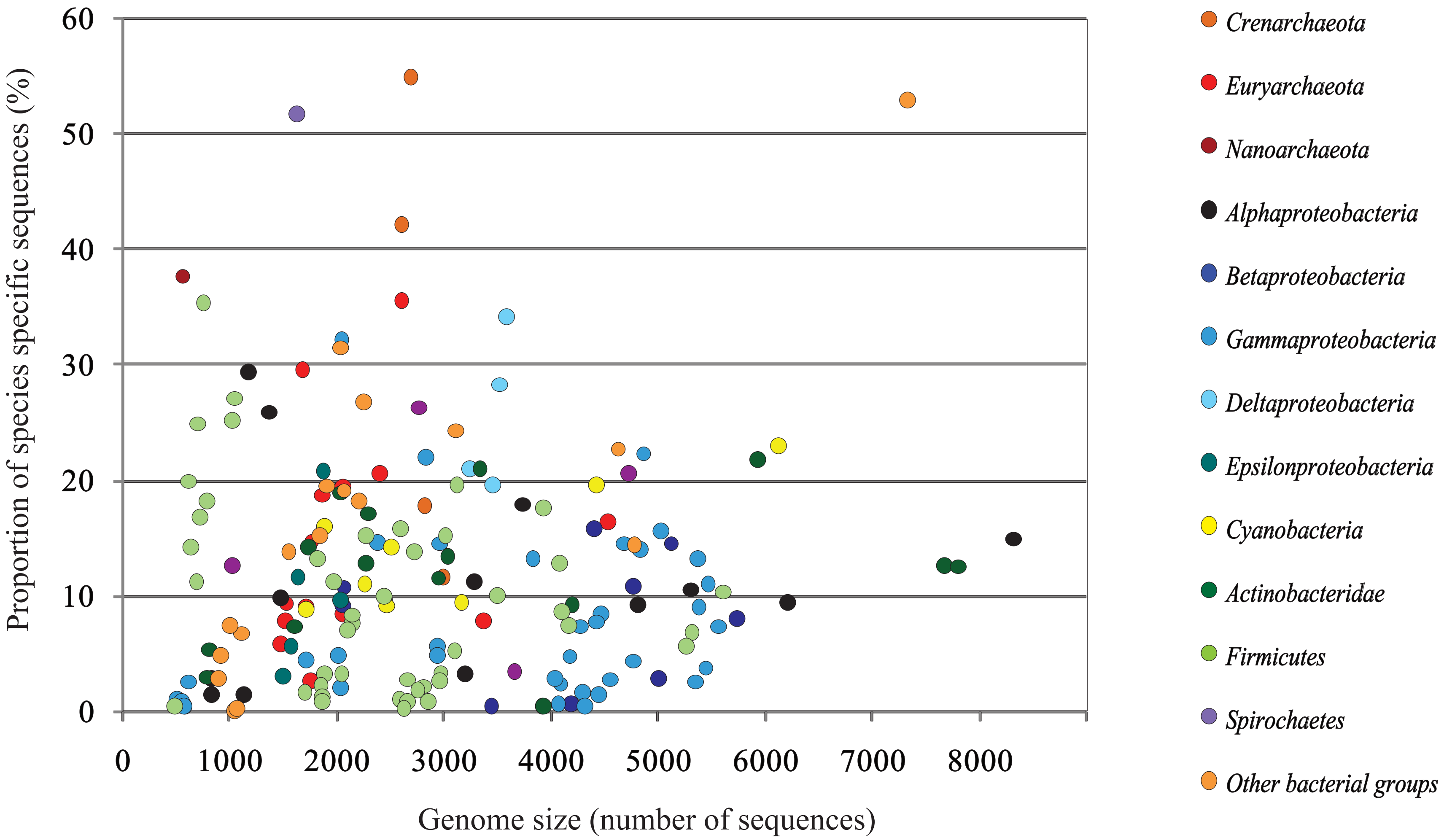

Figure S4. Proportion of species specific sequences as a function of genome size.
Each point indicates an individual bacterial genome colored by its parent taxonomic group (see legend on left). No statistically significant correlation was observed for the complete dataset of genomes. Amongst the individual groups only 'Other bacterial groups' ($r = 0.79$, $p<0.001$, $N=17$) and Cyanobacteria ($r=0.80$, $p<0.01$, $N=8$) showed any significant correlation between genome size and proportion of species specific genes.
