# Supplementary material for: The global landscape of sequence diversity
Source: Genome Biol. 2007 Nov 8;8(11):R238. doi: 10.1186/gb-2007-8-11-r238 (PMC2258180; doi:10.1186/gb-2007-8-11-r238)
Supplement: Additional data file 7 — Relationship between sequence conservation and length. [file gb-2007-8-11-r238-S7.pdf]

(a)

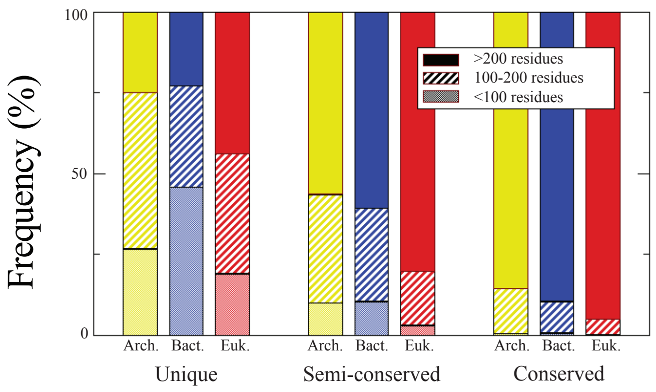

(b)

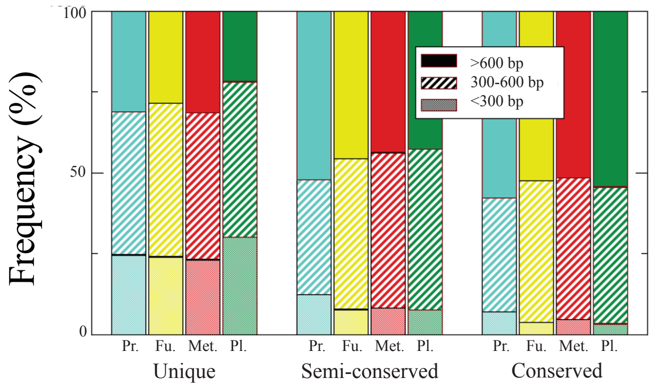

Figure S5. Relationship between gene conservation and sequence length

(a) Frequency of genes with particular length distributions for proteins derived from the complete genome datasets. The three major categories represented are: unique (found only in one species); conserved (genes sharing sequence similarity with genes from the other domains of life; and semi-conserved (the rest). Three datasets are shown: Arch. = proteins derived from Archaea; Bact. = Bacteria and Euk. = Eukaryotes. (b) Equivalent analysis for partial eukaryotic genome datasets. Again three categories are represented: unique (found only in one species); conserved (sequences sharing sequence similarity with sequences from plants, metazoa, fungi and protists); and semi-conserved (the rest). Four datasets are presented representing sequences derived from: Pr. - Protists; Fu. - Fungi; Met. - Metazoa; and Pl. - Plants. Since these are based on nucleotides, the sequence lengths are increased three fold to allow a more direct comparison with the analysis of protein sequences in (A).
